# Genetic Analysis of Platform-Phenotyped Root System Architecture of Bread and Durum Wheat in Relation to Agronomic Traits

**DOI:** 10.3389/fpls.2022.853601

**Published:** 2022-03-25

**Authors:** Michel Colombo, Pierre Roumet, Christophe Salon, Christian Jeudy, Mickael Lamboeuf, Stéphane Lafarge, Anne-Valérie Dumas, Pierre Dubreuil, Wa Ngo, Brice Derepas, Katia Beauchêne, Vincent Allard, Jacques Le Gouis, Renaud Rincent

**Affiliations:** ^1^AGAP, Univ Montpellier, CIRAD, INRAE, Institut Agro, Montpellier, France; ^2^CEFE, Univ Montpellier, CNRS, EPHE, IRD, Montpellier, France; ^3^Univ. Bourgogne, Agroecol Lab, Univ. Bourgogne Franche Comte, AgroSup Dijon, INRAE, Dijon, France; ^4^BIOGEMMA LIMAGRAIN Site Garenne, Chappes, France; ^5^INRAE-Universite Clermont-Auvergne, UMR 1095, GDEC, Clermont-Ferrand, France; ^6^Arvalis – Institut du Végétal, Beauce La Romaine, France; ^7^GQE-Le Moulon, INRAE, Univ. Paris-Sud, CNRS, AgroParisTech, Universite Paris-Saclay, Gif-sur-Yvette, France

**Keywords:** bread wheat, durum wheat, root, association mapping, QTL, breeding, high-throughput phenotyping, genotype x environment interactions

## Abstract

Roots are essential for water and nutrient uptake but are rarely the direct target of breeding efforts. To characterize the genetic variability of wheat root architecture, the root and shoot traits of 200 durum and 715 bread wheat varieties were measured at a young stage on a high-throughput phenotyping platform. Heritability of platform traits ranged from 0.40 for root biomass in durum wheat to 0.82 for the number of tillers. Field phenotyping data for yield components and SNP genotyping were already available for all the genotypes. Taking differences in earliness into account, several significant correlations between root traits and field agronomic performances were found, suggesting that plants investing more resources in roots in some stressed environments favored water and nutrient uptake, with improved wheat yield. We identified 100 quantitative trait locus (QTLs) of root traits in the bread wheat panels and 34 in the durum wheat panel. Most colocalized with QTLs of traits measured in field conditions, including yield components and earliness for bread wheat, but only in a few environments. Stress and climatic indicators explained the differential effect of some platform QTLs on yield, which was positive, null, or negative depending on the environmental conditions. Modern breeding has led to deeper rooting but fewer seminal roots in bread wheat. The number of tillers has been increased in bread wheat, but decreased in durum wheat, and while the root-shoot ratio for bread wheat has remained stable, for durum wheat it has been increased. Breeding for root traits or designing ideotypes might help to maintain current yield while adapting to specific drought scenarios.

## Introduction

Wheat is the largest primary commodity in the world with a production of 730 million tons in 2018. It is estimated that between 2009 and 2011 wheat provided humans with about 18% of their daily intake of calories and 20% of their proteins.^1^ During the second half of the twentieth century, wheat yields in Europe increased tremendously (Calderini and Slafer, 1998) but since the 1990s, they have stagnated in many European countries (Peltonen-Sainio et al., 2009; Brisson et al., 2010). Surprisingly, according to analyses of a variety of trials, there has been no corresponding inflection in genetic progress during this period (Mackay et al., 2011; Oury et al., 2012). One of the main factors that explain this stagnation is abiotic stress, mainly caused by drought and high temperature during grain filling (Brisson et al., 2010). For example, in France, during the 2003 and 2008 droughts, wheat yields dropped by 0.5–1.5 tons per hectare compared to an average year (Agreste Statistic). According to the fifth Intergovernmental Panel on Climate Change assessment report, extreme drought events are very likely to become more frequent in the future due to the lack of rainfall and increased evapotranspiration during grain filling (IPCC, Fifth Assessment Report, 2014). In addition to adapting agronomic practices, one option to mitigate this issue is to develop plants that are more adapted to dry conditions.

Two complementary strategies may help in developing drought-adapted plants. The first is to increase water use efficiency (WUE), for instance, by optimizing evapotranspiration or photosynthesis. The second is to optimize water uptake from the soil (Lynch, 2007). According to modeling studies, each additional millimeter of water extracted from the soil after anthesis would increase wheat grain yield by up to 55 kg ha^–1^ (Manschadi et al., 2006; Christopher et al., 2013). Atta et al. (2013) found that crop yield, water extraction, and WUE were influenced by root distribution (root length, root diameter, or root length density at different depths) in water deficit conditions, where root traits explained 45% of grain yield variance. Similarly, Postic et al. (2019) found a significant correlation (*r*^2^ > 0.40) between wheat root length surface density at the anthesis stage and grain yield under various conditions. As roots are responsible for water and nutrient uptake, they are a key aspect to consider when developing varieties with increased tolerance to abiotic stress.

In the past, breeding mostly focused on above-ground traits so any effects of breeding on root traits are probably derived from an indirect selection. Some authors found evidence of indirect selection for root traits (Siddique et al., 1990; Zhu et al., 2019) whereas others did not (Lupton et al., 1974; Cholick et al., 1977; Friedli et al., 2019). In any case, no clear functional link between root traits and adaptation to a particular environment has been found so far (Hurd, 1974; Chloupek et al., 2006; Waines and Ehdaie, 2007; Wojciechowski et al., 2009; Bai et al., 2013; Elazab et al., 2016; Aziz et al., 2017). A clear understanding of the adaptive value of root traits could be relevant to speed up breeding for adaptation to a specific environment.

Studying root traits is highly complex because field root phenotyping is difficult, expensive, and labor-intensive. Dating back to Weaver in 1926 who excavated the whole root system architecture (RSA) and until recently, phenotyping mainly focused on easily measurable traits (e.g., insertion angle of nodal roots, root lengths) that only roughly describe the RSA (Palta et al., 2011) with methods that tended to be destructive. Since then, soil cores, mesh bags, and shovelomics techniques have been developed (Sharma et al., 2011; Trachsel et al., 2011; Lou et al., 2015). A more ambitious non-destructive solution to overcome field phenotyping difficulties is provided by high-throughput phenotyping platform (HTPP) technology explicitly dedicated to the root system characterization (Svane et al., 2019).

In an HTPP, the root traits of a large number of individual plants can be observed in controlled conditions at early developmental stages (Kuijken et al., 2015). The growth methods used in HTPP vary widely, e.g., hydroponics (Ayalew et al., 2015), pots filled with soil (Cao et al., 2014), germination bags (Robertson et al., 1979), gel-filled chambers with transparent walls (Bengough et al., 2004; Manschadi et al., 2006; Christopher et al., 2013), paper-based “cigar roll” system (Zhu et al., 2006; Li et al., 2011), germination paper with growth pouch for wheat and maize (Hund et al., 2009; Atkinson et al., 2015), and compressed columns of soil with X-rays to detect roots (Doussan et al., 2006; Gregory et al., 2009). A recent review (Paez-Garcia et al., 2015) highlighted the strengths and the weaknesses of most of these methods. The main advantages common to these different methods are to enable high-throughput and cheap phenotyping of large genetic panels to facilitate QTL detection, association mapping, or the calibration of genomic prediction models for RSA traits. However, HTPP does not record phenotypes that are robustly relevant for later stages of the crop cycle because the explored soil volume becomes restricted after a few days to a few weeks in pots or root growth boxes (Palta et al., 2011).

Until now, HTPP has shown contrasting results in detecting the relation between root traits measured in controlled conditions and field performances. Some wheat traits affecting grain yield in specific environments have been identified. These include higher root density at depth to improve deep water capture (O’Brien, 1979), deeper roots (Hurd, 1974), faster elongation rates to extract water from deeper soil layers (O’Brien, 1979), a narrower diameter of the xylem vessel in the seminal roots to conserve soil water (Richards and Passioura, 1989), reduced insertion angle of seminal roots to access water from deeper soil layers (Nakamoto and Oyanagi, 1994; Manschadi et al., 2006), and more integrative traits such as higher root-shoot ratio to improve water capture across the soil profile (Siddique et al., 1990; Reynolds et al., 2007). However, these traits observed in controlled conditions, reviewed by Palta et al. (2011), never passed field validation trials and to our knowledge, have not been used in breeding programs. While the assumption when using an HTPP is that genotypes differing at an early developmental stage will also differ later under field conditions, the relationship between early RSA traits and mature RSA traits might depend on environmental conditions and might not be verified in all environments.

The lack of field validation may be due to genotype × environment (G × E) interactions. Indeed, RSA ideotypes to optimize agronomic performances are different from one environment to another. As a result, relationships between root traits measured in controlled environments and agronomic performances are inconsistent across locations and/or years (Canè et al., 2014; Xie et al., 2017; Roselló et al., 2019). For instance in water-rich environments, a large RSA enables the efficient uptake of water and nutrients (concentrated in topsoil) especially during spring rainfall, increasing grain filling (Waisel et al., 2002). In a water-scarce environment, large or deep RSA systems might be metabolically too costly for a plant if it does not result in additional water uptake [for instance by accessing the deeper soil layer, Palta et al. (2011)] and might cause yield loss. Thus, when water is scarce, investing fewer resources in root development could spare more assimilates for above-ground organ growth (Elazab et al., 2016). Thus, finding the most adapted RSA to a specific environment will require detailed environmental characterization to design the corresponding root ideotype and understand the variability in the relationship between RSA and agronomic performances.

The HTPP, nevertheless, provides large amounts of heritable root phenotypic data, some of which have led to the discovery of many QTLs for RSA in controlled conditions (Hund et al., 2011; Soriano and Alvaro, 2019). QTL mapping is a powerful tool to understand how plants function and to establish relationships between traits through co-localization. Understanding the link between early-stage RSA features and in-field performance in specific environments requires better characterization of environmental variability. In this study, our main objectives were: (i) to analyze the genetic variability and the underlying genetic architecture of a range of RSA traits measured in an HTPP for one durum wheat and two bread wheat panels (Jeudy et al., 2016), (ii) to evaluate the indirect effect of breeding in the last 80 years on RSA traits, and (iii) to identify relationships between RSA traits measured in HTPP and field productivity traits at the trait or QTL level with environmental characteristics.

## Materials and Methods

### Genetic Materials and Genotyping

For bread wheat (*Triticum aestivum*), two previously assembled panels were studied. The first panel, BW_div, is composed of 450 varieties sampled from 4,506 varieties in a worldwide collection (Balfourier et al., 2019) by minimizing linkage disequilibrium and by imposing agronomic constraints (phenology and plant height). The second panel, BW_elite, is composed of 265 varieties most of which are registered in Europe and were grown in France between 1980 and 2010 (Ly et al., 2017; Rincent et al., 2018, 2019; Touzy et al., 2019; Beral et al., 2020; Robert et al., 2020). The complete list of varieties is indicated in Supplementary Table 1.

The durum wheat (*Triticum durum*) panel includes 100 elite European lines (DW_EPO_elit), as well as 100 pre-breeding lines (DW_EPO_div). The latter evolutionary pre-breeding population (EPO) lines are derived from an open-pollinated population based on the intercrossing of about 650 accessions from wild subspecies and elite lines. To promote the allogamy rate, male sterile plants have been included and maintained in this population at a frequency of 20% over successive generations (David et al., 2014). At the twelfth cycle, 500 plants were randomly sampled leading to 480 EPO single-seed descent derived lines, fixed after five self-pollinated generations. The 100 EPO lines observed here are a random subsample of the total EPO set. The complete list of varieties is indicated in Supplementary Table 1.

The wheat accessions were genotyped on an improved Axiom array based on the TaBW280K SNP chip (Rimbert et al., 2018) and composed of 409,695 SNP markers. We extracted the SNP and presence/absence variants (off-target variants). Missing values were imputed with the observed allele frequency in the panel for the corresponding marker. Redundant markers and markers with a minor allele count under 20 were filtered out. After applying these filters, we retained 222,467 markers for bread wheat and 79,910 for durum wheat. Alleles with the highest frequency were chosen as reference alleles.

### Growth Conditions and Experimental Design in the 4PMI High-Throughput Phenotyping Platform

Plants were phenotyped in three experiments in the Plant Phenotyping Platform for Plant and Microorganism Interactions (4PMI) HTPP at INRAE-Agroecology (Jeudy et al., 2016). Seeds were germinated at room temperature for 2 days and then homogenous seedlings were selected and inserted into RhizoTubes. Each plant, considered as a replicate, was grown in controlled conditions for three weeks. The growth media was a 25:75 mixture of sand (Biot B4, Silices et Refractaires de la Méditerranée) and perlite. Nutrition was provided in a solution containing: 1 mM K_2_HPO_4_, 5 mM KNO_3_, 2.5 mM Ca(NO_3_)_2_, 2 mM MgSO_4_, 2 mM CaCl_2_, 50 μM Fe EFTA, 10 μM H_3_BO_3_, 4.5 μM MnCl_2_, 0.2 μM Mo(Na_2_O_4_), 0.4 μM CuCl_2_, and 0.7 μM ZnCl_2_. A RhizoTube has a diameter of 17 cm and a depth of 49.5 cm (Jeudy et al., 2016).

Each of the three experiments (September 2017, July 2018, September 2019) consisted of 1,125 RhizoTubes, each containing two plants of the same variety which were divided between two experimental units, one with 475 RhizoTubes, the other with 650 RhizoTubes. Plants were watered in the RhizoTubes with 250 ml of the same nutrient solution from day 1 to 4. The water content was maintained at the field capacity from day 5 to day 26 (harvest day) to ensure that no hydric stress occurred. The available water capacity was 1,542 mm. The temperature was on average 21.8°C during the day and 18.4°C during the night. Rhizotubes with varieties from the same wheat panel were grouped together. The RhizoTubes were grouped into 23 blocks in the two experimental units, with six check varieties (four bread wheats and two durum wheats) grown in each block to take any spatial effect into account. The 1,125 RhizoTubes were as follows:

- Nine hundred and fifteen rhizotubes for 915 cultivars (200 durum wheats, 450 bread wheats from the diversity panel, and 265 bread wheats from the elite panel).
- Seventy two rhizotubes for image calibration (18 cultivars, four replicates).
- One hundred and thirty eight rhizotubes for the six check genotypes (two from the BW_div panel, two from the BW_elit panel, and two from the BD_EPO panel). Each check genotype was present in one rhizotube (two plants) in each block.

### Measurements in the Phenotyping Platform

Temperatures were measured in 66 locations spread over the experimental units and incident solar radiation was measured in 12 locations to correct for any spatial heterogeneity in the experimental units. Kriging was performed with the krige function of the gstat R package (Pebesma, 2004) to extrapolate temperatures and radiation values for each rhizotube of the experimental unit every 15 min. The parametrization of the kriging model was optimized using leave-one-sensor-out cross-validations.

Two types of traits were measured in control conditions. First, RSA-related traits were extracted from image analysis early during plant development. Five days after implantation, we measured the seminal root angle [angle between the leftmost and the rightmost seminal roots at 3 cm from the seed (Richard et al., 2015)] and the number of seminal roots. Every 4 days, we measured the root width, root depth, maximum rooting depth and depth_80 (defined as the depth above which 80% of root image pixels are located), the gravity center of the RSA (CGY), and convex hull which represents the volume explored by the roots. These traits are all descriptors of the spatial distribution of the roots in the soil. The following root and shoot traits were also measured (destructively) at the end of the experiment, 21–24 days after sowing between the four and five leaves stage: above-ground biomass, number of leaves on the main stem, number of tillers, root biomass and root-shoot biomass ratio (root-shoot ratio). These traits are good indicators of biomass allocation between organs.

### Growth Conditions and Phenotyping in the Field

The BW_elite panel was grown in fields from 2012 to 2016 in 42 environments, partly representing the diversity of growing conditions in France (Ly et al., 2017; Rincent et al., 2019; Touzy et al., 2019; Robert et al., 2020). These 42 environments correspond to 26 year-location combinations, including two treatments for 16 of them: irrigated (WW) vs. rainfed (WD) treatments, well-watered (WW) vs. rainout shelter (RO), and high (HN) vs. low (LN) nitrogen fertilizer treatment. Environmental variables were computed in Rincent et al. (2019) to best characterize the growth conditions of each year × location combination. This dataset was previously used in Touzy et al. (2019) to cluster environments in four groups of water stress patterns to find drought scenario-specific QTLs. The BW_div panel was grown in 12 environments split in different locations in France and with contrasting levels of abiotic stress (Supplementary Table 2).

The DW_EPO panel was grown in 10 environments in 2018 or 2019 located in three main durum production areas in France (Paris basin, Southeast France and Southwest France) and in Italy (Po Valley) under both non-limiting (NL) and limiting (L) conditions (water and nitrogen, Supplementary Table 3). In total, this panel was tested in a network of 10 year-location-treatment combinations using two fully replicated designs except for two trials where only 50% of the genotypes were replicated. A water stress index based on the available water capacity of the soil was computed between the key growth stages to analyze the influence of environments on the relationships observed. Based on soil water balance at each stage of the plant cycle (emergence to tillering, tillering to heading, heading to flowering, and flowering to maturity), the water stress index varied from 0 indicating a severe water-stressed environment to 1 for a water-rich environment. Hereafter the term “environment” will refer to a site × year × treatment combination.

In each environment, grain yield at 0% moisture content (GY, kg ha^–1^), grain protein content (GPC,% of total dry weight), grain protein deviation (GPD), thousand kernel weight (TKW, g), grain number per m^2^ (GN), and date of earing (DOE, 50% of ears emerged, expressed in Julian days) were measured.

### Correction for Spatial Effects and Estimation of Heritability for Platform-Phenotyped Traits

The width, depth, convex hull, CGY, and depth_80 at 10 days were extracted from corrected trajectories for each plant root system. Corrected trajectories were obtained by smoothing each plant trajectory with the function locfit of the locfit package in R using 5-time points for smoothing (Loader, 1999).

A spatial model using the R package SpATs (Rodríguez-Álvarez et al., 2018) was fitted for the five traits mentioned above and for the seven traits measured at the end of the three 4PMI HTPP experiments. In the model, the sum of temperatures (from baseline of 0°C) at the RhizoTube level was used as a covariate and the block effect was declared as random. This model uses a two-dimensional P-spline surface to model spatial heterogeneity in the greenhouse, thus correcting for spatial trends with smoothing splines. Plants with root or aerial biomasses below 0.5 g were removed from the analysis and considered as outliers. In addition, for each trait, plants with residuals above a threshold (defined by visual examination) were also removed from the analysis (Supplementary Table 4).

Finally, for each trait, genotypes falling outside the distribution were removed by visual examination. This included one genotype for convex hull (value > 40,000), one genotype for depth (value > 400), one genotype for width (value > 200 mm) and one genotype for CGY (value > 155 mm).

Marginal means (best linear unbiased estimation, BLUE) per genotype were extracted from the SpATS model to perform further analysis. At this stage, a single genotype from the BW_div panel with trait values clearly outside the distribution was removed.

The generalized heritability of the corresponding adjusted means was computed for each trait as in Rodríguez-Álvarez et al. (2018) using the SpATS function “getHeritability,” after defining genotype as a random effect.

Adjusted means and heritabilities of the agronomic traits (field trials) had already been computed in previous studies for BW_elit (Rincent et al., 2019; Touzy et al., 2019). For the BW_div and DW_EPO panels, adjusted means were extracted using SpATs (Rodríguez-Álvarez et al., 2018).

### Correlations Between High-Throughput Phenotyping Platform Root System Architecture Traits and Agronomic Traits Measured in Fields

The linear correlations between traits measured in the HTPP and agronomic traits measured in fields were computed. As DOE was significantly correlated to yield and yield components in some environments (Supplementary Figures 1–3), residuals from the linear regression of yield on DOE were computed to correct yield variables GY, GN, and TKW for the DOE effect per environment. The corrected variables are named GYC, GNC, and TKC respectively. This avoided spurious correlation between yield and RSA traits purely due to phenology (Canè et al., 2014; Xie et al., 2017).

The square root of the determination coefficients, that is, the value of R for each model, was computed. To account for multiple testing, we computed critical R with a Bonferroni correction based on the number of independent HTPP traits × the number of independent environments. The numbers of independent HTPP traits and independent environments were estimated as the number of principal component analysis (PCA) axes accounting for at least 90% of the variability of respectively the HTPP traits dataset and the GY dataset. We identified six independent variables for the HTPP traits in all panels and respectively 30, 21, and 21 independent variables in BW_elite, BW_div, and DW_EPO multi-environment trials, which resulted in 180, 126, and 126 independent tests for the different panels. PCA was computed using the PCA function of the FactomineR package (Lê et al., 2008).

Multiple linear models were also used to relate agronomic variables to several root traits measured in the HTPP. Again, the R-value (Pearson correlation between the value predicted by the model and the observed value) of the models was analyzed for each environment independently.

### Quantitative Trait Locus Detection

The mixed model used for association mapping (Yu et al., 2006) was as follows:

*Y* = *X*β + *Zu* + ε with Var(*u*) = Kσ^2^_G_ and Var(ε) = Iσ^2^_e_, where *u* is the random polygenic effect, β represents the vector of fixed effects (intercept, population structure for durum wheat panel, and dosage of the tested SNP), K represents a matrix of genetic relatedness between individuals (see below), I is the identity matrix, X and Z are incidence matrices for fixed and random effects, respectively, and σ^2^_G_ and σ^2^_e_ are the respective polygenic and error variances. K was computed following the VanRaden (2008) equation:


$$K=V\,V^{\prime }/2\,\sum p_{i}\,\left(1-p_{i}\right)$$

with V being the centered marker matrix and p_i_ the allele frequency at marker i.

Each SNP was tested successively with the function GWAS from the R package statgenGWAS which is based on the method presented in Kang et al. (2010). The EMMA algorithm was used to test the effects as the Fisher exact test was computationally too demanding. In the efficient mixed-model association (EMMA) algorithm, the variance of the residual and polygenic effects is estimated only once by fitting a model with no SNP. The first principal component of the PCA (% of variability explained by this axis was above 5%) on genotypic data was considered as a fixed effect in the G + Q model in DW_EPO to correct for population structure. QQ-Plots were systematically inspected to check that the false positives were correctly controlled.

#### Selection of Significant SNPs

We computed a false discovery rate (FDR) threshold of 30% as described by Benjamini (2010). This chosen risk of false positives is high, but we expect colocalizations between multiple traits/environments to highlight the importance of some QTLs.

#### Computation of Quantitative Trait Locus Boundaries

To define QTL boundaries, we used a method inspired by Cormier et al. (2014). For each chromosome and each trait, linkage disequilibrium (LD) was computed between significant markers. Then markers were clustered by LD blocks. QTL boundaries were defined as the minimum and maximum map position of significant markers belonging to the same LD block. QTLs of different traits were considered to overlap when they had at least one common significant marker and were located at a physical distance less than one-tenth of the total physical length of the chromosome as presented in Cormier et al. (2014).

The R^2^ estimator from Hill and Robertson (1968) was used to assess LD. These LDs were square roots transformed to approximate a normally distributed random variable as in Breseghello and Sorrells (2006).

Clustering was performed with the UPGMA method using a cutoff of 1–“critical R^2^”. Critical R^2^ (R^2^c) was defined as the 99.9^th^ percentile of the distribution of unlinked R^2^ computed between 10,000 pairs of markers randomly sampled from different chromosomes. This threshold accounts for the risk of 0.1% of markers being in LD by chance.

For durum wheat, the panel contains both EPO and elite alleles. Therefore, portions of the genome corresponding to elite materials create an artificial correlation between markers on different chromosomes. We accounted for this effect by using corrected R^2^c for relatedness using the package LdcorSV (Mangin et al., 2012). For bread wheat, R^2^c was 0.23 and for durum wheat, R^2^c corrected for relatedness was 0.38 rather than 0.92 without correction.

#### Colocalization and Effect of High-Throughput Phenotyping Platform Root Quantitative Trait Loci On-Field Agronomic Traits Explained by Environmental Variables

Quantitative trait loci (QTLs) were considered to co-localize when they had at least one marker in common in their confidence interval. Note that because of the high LD extend of the durum wheat panel, co-localization might be due to both genetic linkage or pleiotropic effects of the loci. For each HTPP QTL co-localizing with a field agronomic trait QTL, for each agronomic productivity trait (GN, GY, TKW, GNC, GYC, and TKC), we regressed environmental variable values onto the QTL effect computed in each environment. Thus, for each HTPP QTL, we computed six regressions. Significant regressions were detected with a Bonferroni threshold accounting for the number of significant SNP tested × the number of independent variables (estimated as the number of independent axes of a PCA accounting for 90% of the variability of the dataset; n_Test_ = 66 SNP × 13 environments for BW_elite and n_Test_ = 44 SNP × 2 environments for the durum wheat panel). Correlations were tested only in BW_elite and in DW_EPO as environmental variables were only available for these two panels. Multi-trait models were tested using multiple regression with the lm and ANOVA function of base R.

### Identifying Past Selection on Root Traits

To test the hypothesis of past indirect selection of root traits, linear regression between adjusted mean values and dates of cultivar release was performed for 81 elite cultivars of durum wheat and on all cultivars registered in France for bread wheat. Bonferroni correction was performed to account for multiple-trait testing.

## Results

### Genetic Variability and Correlation Between High-Throughput Phenotyping Platform Traits

Even though the number of replicates in this experiment was low, heritability values were moderate to high (0.42 < H^2^ < 0.82) which reflects the good quality of the phenotyping (Table 1). In general, traits related to biomass and aerial morphology were more heritable than root architecture traits.

**TABLE 1:** Heritability of platform traits in the different panels.

	Durum wheat	Bread wheat
Above-ground biomass	0.76	0.67
Number of Leaves	0.82	0.8
Number of tillers	0.76	0.72
Root biomass	0.64	0.59
Root-to-shoot ratio	0.81	0.74
Root number	0.63	0.66
Root angle	0.52	0.64
Depth	0.45	0.69
Width	0.62	0.65
Depth_80%	0.42	0.6
Convex hull	0.44	0.64
CGY	0.5	0.63

Considerable variability was observed in all panels for all traits. Standard deviations computed on marginal means of HTPP traits ranged from 4.5% of the mean trait value for the number of leaves to 26.0% for root angle. Traits with the least variability were leaf number, root depth, and root depth_80 (Table 2 and Supplementary Figure 4). Root biomass was relatively less variable than above-ground biomass, which could partly explain the high correlation between the root-shoot ratio and above-ground biomass. Root angle was the trait with the highest variance. In the elite bread wheat panel, phenotypic variances were relatively smaller for root-shoot ratio, root biomass, number of leaves, number of tillers, depth, depth_80, convex hull, and above-ground biomass than the corresponding variances in the other panels.

**TABLE 2:** Coefficient of genetic variation of platform traits in the different panels.

	Bread wheat div	Bread wheat elit	Durum wheat
Above-ground biomass	0.111	0.101	0.149
Number of leaves	0.045	0.037	0.047
Number of tillers	0.174	0.146	0.197
Root biomass	0.087	0.086	0.102
Root-to-shoot ratio	0.071	0.066	0.084
Root number	0.070	0.076	0.059
Root angle	0.169	0.178	0.125
Depth	0.057	0.039	0.028
Width	0.188	0.189	0.180
Depth_80%	0.053	0.048	0.031
Convex hull	0.245	0.222	0.138
CGY	0.052	0.044	0.035

**FIGURE 1:**
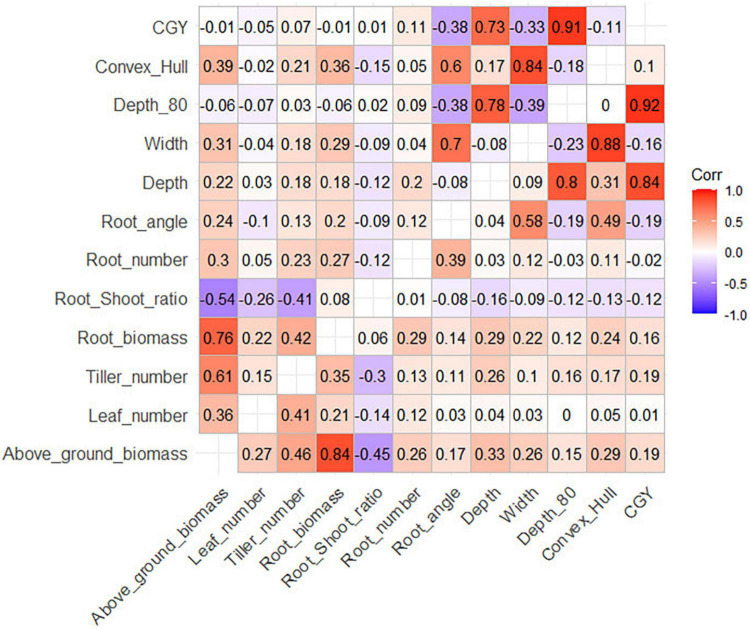
Correlation matrix between root traits (upper panel: durum wheat, lower panel: bread wheat). Critical R for the durum wheat panels are R = 0.139 (α = 5%), R = 0.182 (α = 1%) and for the bread wheat panel R = 0.073 (α = 5%), R = 0.096 (α = 1%).

Significant correlations between root traits were similar in the three panels (Figure 1 and Supplementary Figures 5, 6). All above-ground traits were correlated with each other, and root biomass and shoot biomass was strongly correlated (*r* = 0.77 for durum wheat, *r* = 0.84 for bread wheat). As a result, the root biomass also significantly correlated with most other above-ground traits, for example, with the number of leaves (*r* = 0.21 for durum wheat, *r* = 0.22 for bread wheat), and the number of tillers (*r* = 0.35 for durum wheat, *r* = 0.43 for bread wheat). Interestingly, the root-shoot ratio was negatively correlated with the number of leaves (*r* = −0.26 for durum wheat, *r* = −0.14 for bread wheat). This indicates that at a given date at the beginning of the season, late cultivars that have fewer leaves (due to the longer phyllochron) tended to allocate more resources to their roots than early cultivars that have more leaves at an equivalent stage.

We also observed differences between the elite and diverse bread wheat panels (Supplementary Figures 5, 6). Root angle was generally a better proxy of the depth-related measurements Depth, Depth_80, and CGY in BW_elite (respectively, *r* = −0.02, −0.23, −0.24) than in BW_div. The correlations between biomass measurements (root and above-ground) and geometric measurements (Depth, Width, CGY, convex_hull) were relatively stronger in BW_div than in BW_elite. Similar phenomena were observed for durum wheat between the elite and EPO sub-panels.

### Phenotypic Correlations Between High-Throughput Phenotyping Platform and Agronomic Field Traits

#### Single Trait Approach

**FIGURE 2:**
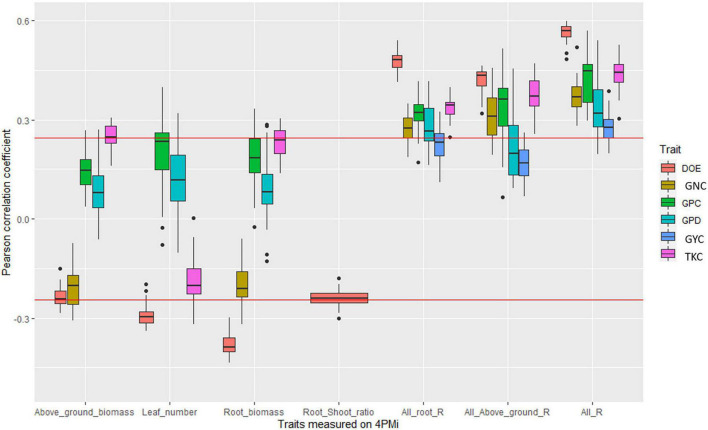
Boxplot of correlation coefficients between platform traits and agronomic values measured in fields in the bread wheat elite panel. Each point of a boxplot represents one of the 42 environments. Only boxplots with a significant correlation in at least one environment are plotted. The three last series of boxplots correspond to the coefficient of determination of the linear regression between agronomic trait and all root traits measured on the platform (All_root_R), all traits measured on the platform (All_R), and all above-ground traits measured on the platform (all_above_ground_R). The red line corresponds to significance levels at the 5% thresholds.

In BW_elite, significant correlations were found between grain yield and root traits but these correlations were not significant after correcting for phenology (DOE measured under field conditions) indicating that differences in earliness among the lines partly explain root trait variability (Figure 2, Supplementary Table 5, and Supplementary Figure 7). Among yield components, TKC was positively correlated in almost half of the environments with root and shoot biomass measured in HTPP.

**FIGURE 3:**
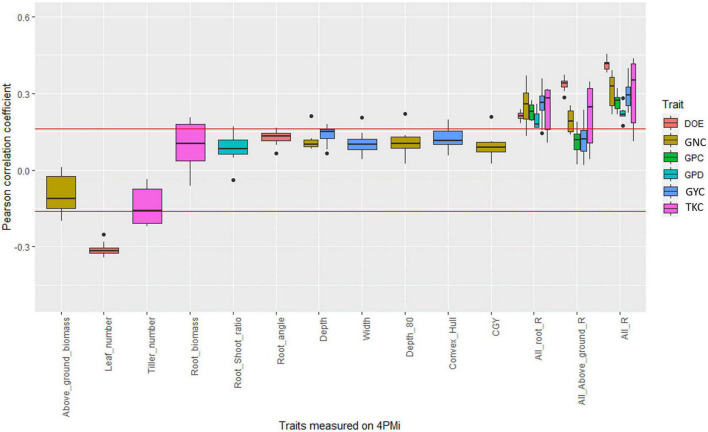
Boxplot of the correlation coefficient between platform traits and agronomic values measured in fields in the bread wheat diversity panel. Each point of a boxplot represents one of the 17 environments. Only boxplots with a significant correlation in at least one environment are plotted. The three last series of boxplots correspond to the coefficient of determination of the linear regression between agronomic trait and all root traits measured on the platform (All_root_R), all traits measured on the platform (All_R), and all above-ground traits measured on the platform (all_above_ground_R). The red line corresponds to significance levels at the 5% thresholds.

For BW_div, most identified correlations disappeared after correction for earliness. However, significant positive correlations remained between root depth and GYC in three environments (17INRmon_NUE_HN, 17SYNmoi_NUE_HN and 17SYNmoi_NUE_LN, Figure 3, Supplementary Table 6, and Supplementary Figure 8). In another environment (17LIMcas_WUE_SEC), root depth was significantly correlated with GNC (*r* = 0.21, *p*-value < 0.05). Convex hull which represents the soil exploration capacity of the roots in the HTPP, was positively and significantly correlated with GYC in three environments (17BAYmil_WUE_IRR, *r* = 0.16; 17INRcle_WUE_IRR, *r* = 0.20; 17INRmon_NUE_LN, *r* = 0.17, *p*-value < 0.05). The strongest correlation involving convex hull was for the environment 17INRcle_WUE_IRR. That was also the environment where root width was the most significantly correlated with GYC (*r* = 0.21, *p*-value < 0.05), but root depth was not (*r* = 0.13, NS).

In one non-irrigated environment (17BAYmil_WUE_SEC), the root-shoot ratio was significantly correlated to grain protein deviation (*r* = 0.17, *p*-value < 0.05) but root biomass and above-ground biomass were not (*r* = −0.02, *r* = −0.08, Figure 3 and Supplementary Figure 8).

**FIGURE 4:**
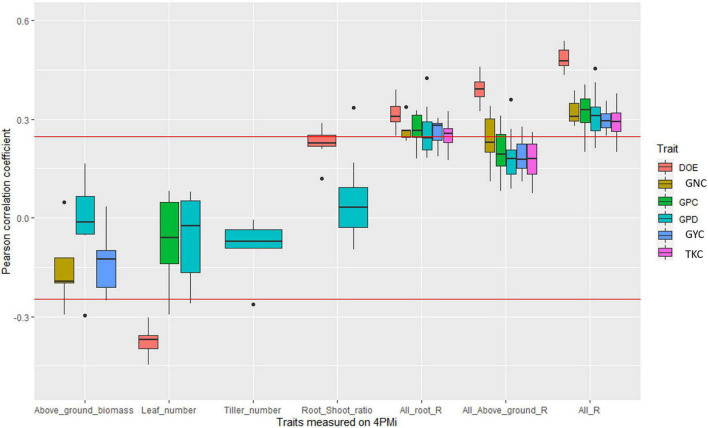
Boxplot of correlation coefficients between platform traits and agronomic values measured in fields in the durum wheat panel. Each point of a boxplot represents one of the 10 environments. Only boxplots with a significant correlation in at least one environment are plotted. The three last series of boxplots correspond to the coefficient of determination of the linear regression between agronomic trait and all root traits measured on the platform (All_root_R), all traits measured on the platform (All_R), and all above-ground traits measured on the platform (all_above_ground_R). Red line corresponds to significance levels at the 5% thresholds.

In DW_EPO_Elit, no significant correlation was observed between root traits and field traits. However, in two water-scarce environments the root-shoot ratio was positively though not significantly correlated with GYC (INRAE_2019_opt, r = 0.22; QUALPREST_2019, *r* = 0.19). In addition, in the environment QUALPREST_2019, GPD was also significantly correlated with root-shoot ratio (*r* = 0.33, *p*-value < 0.05) and with above-ground biomass (*r* = −0.30, *p*-value < 0.05, Figure 4, Supplementary Table 7, and Supplementary Figure 9).

#### Multi-Trait Approach

All the root traits measured in the 4PMI HTPP, when taken together, rarely explained more than 10% of the variability of agronomic traits whatever the panel (Figures 2–4, Supplementary Tables 5–7, and Supplementary Figures 7–9). The proportion of variance (R of the linear model) explained by multi-trait linear regression between GYC measured in the different agronomic environments and all root traits were negatively correlated with most water-stress indexes in the durum wheat panel (Supplementary Table 8). These correlations ranged from −0.51 to −0.79 depending on the timing of the water-stress index considered (ranging from the emergence stage to the maturity stage). This indicates that root traits measured under controlled conditions are more efficient at predicting agronomic performance in water-stressed environments than in other environments. This was not observed in the bread wheat panels.

### Quantitative Trait Locus Detection and Colocalization Between High-Throughput Phenotyping Platform and Field Traits

#### High-Throughput Phenotyping Platform and Agronomic Traits Quantitative Trait Locus Colocalization and Relationship With Environmental Variables in Bread Wheat

**FIGURE 5:**
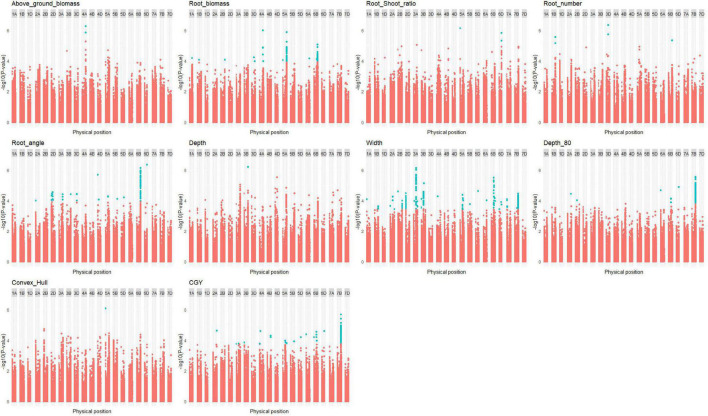
Manhattan plot of root traits in the bread wheat panels (elite + diversity). Only Manhattan plots with at least one quantitative trait locus (QTL) are plotted. Significant SNPs with false discovery rate (FDR) thresholds at 10% are colored in blue.

In the bread wheat panels, 100 QTLs were found (Supplementary Table 9) for the HTPP traits (Figure 5 and Supplementary Figures 13, 14). The co-localization of these QTLs with QTLs found in the multi-environment trial for agronomic traits is presented in Supplementary Figure 15.

**FIGURE 6:**
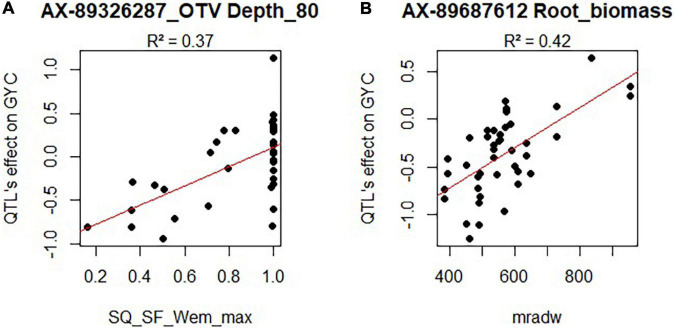
Allelic effect of two root traits QTLs (**A**: Depth_80, **B**: Root_Biomass) on GYC in several environments as a function of the environmental variable measured in these same environments. SQ_SF_Wem_max represents a water stress indicator from elongation to the meiosis period (Rincent et al., 2019). Values closed to one represent non-stressed environments. mradw represents the average radiation from sowing to the beginning of stem elongation.

For the HTPP QTLs that colocalized with agronomic QTLs, we tried to identify an environmental variable that correlated significantly with the environment-specific agronomic QTL effect. This was possible for 6 QTLs (*r*^2^ > 0.34; Bonferroni correction for 66 SNP tested ×13 independent variables at a risk of 5%). Among these six root QTLs, one was related to both root and above-ground biomass, and another was related only to agronomic variable non-corrected by DOE. Thus, we did not consider them in the following lines. Significant relationships between agronomic QTL allelic effects and environmental variables are presented in Figure 6 and in Supplementary Table 10 and Supplementary Figure 16.

First, a QTA with a positive influence on Depth_80 was beneficial for GYC in a water-stressed environment (during the period of elongation to meiosis) and had a negative impact in non-stressed environments (*r*^2^ = 0.37; Figure 6A).

Second, a quantitative trait allele (QTA) altering root and above-ground biomass in the HTPP also had a differential effect on GYC depending on the amount of incident radiation during winter. This QTA had a negative impact on GYC in low radiation conditions and a positive effect in high radiation conditions (*r*^2^ = 0.42; Figure 6B).

Third, one QTA altering root biomass had a contrasting effect on GYC depending on the number of days with temperatures below 0°C between the beginning of stem elongation and flowering. The effect was negative or null in warm environments and positive in cold environments (*r*^2^ = 0.46).

Finally, another QTA associated with high root angle had a significant negative effect on TKC only in the most stressed environment (AVRgre2014sec) during the period from elongation to meiosis (combined water, temperature, and nitrogen stress) of the multi-environment trial.

#### High-Throughput Phenotyping Platform and Agronomic Traits Quantitative Trait Locus Colocalization and Relationship With Environmental Variables in Bread Wheat in Durum Wheat

**FIGURE 7:**
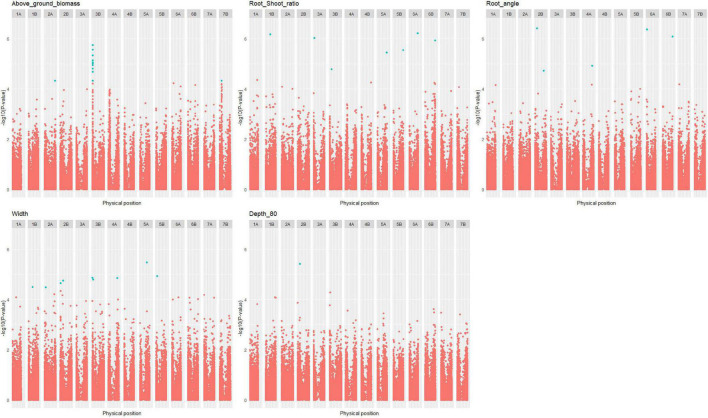
Manhattan plot of root traits in the durum wheat panel. Only the Manhattan plot with at least one quantitative trait locus (QTL) is plotted. Significant SNPs with false discovery rate (FDR) thresholds at 10% are colored in blue.

Thirty-four QTLs related to root traits measured under 4PMI HTPP conditions were found in the durum wheat panel (Supplementary Table 11): one for above-ground biomass, one for depth_80, seven for root angle, seven for root-shoot ratio, three for convex hull, and fifteen for width (Figure 7 and Supplementary Figures 17, 18). Most of these QTLs co-localize with QTLs of agronomic traits measured in fields (Supplementary Figure 19). Because some regions of the genome have a high LD extent, some QTL had wide confidence intervals, which may generate colocalizations involving numerous genes. We could not explain the environment-specific allelic effect of the HTPP QTLs on agronomic traits with environmental variables (highest R = 0.74 < R_critical_ = 0.91). In addition, the highest correlations were obtained with agronomic variables that were not corrected for earliness, indicating that earliness plays an important role in this correlation.

### Root Trait Variation in Varieties From Different Registration Periods

**FIGURE 8:**
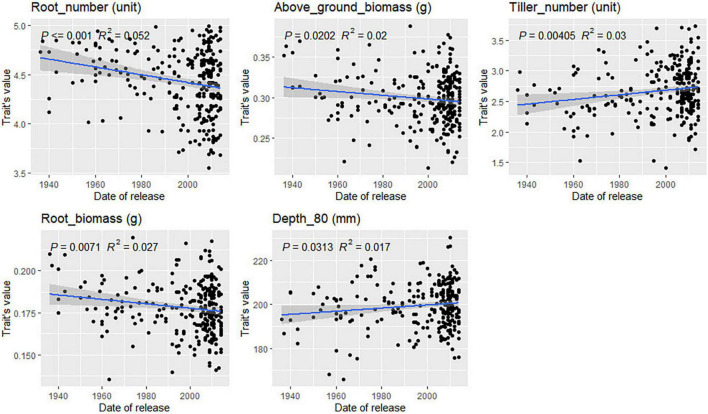
Evolution of bread wheat platform traits during the twentieth century. Only significant relations are reported based on the Pearson correlation coefficient.

**FIGURE 9:**
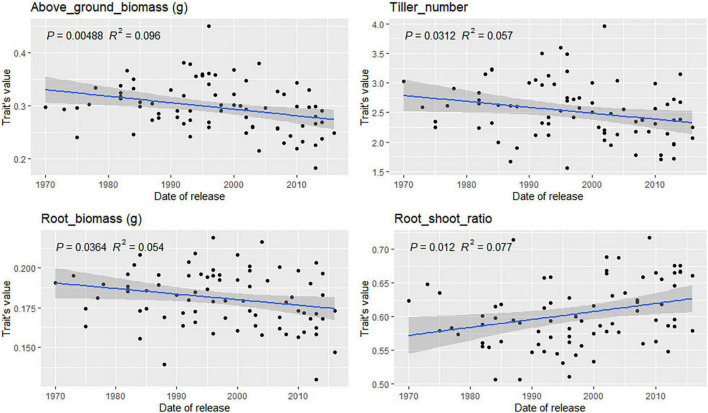
Evolution of durum wheat platform traits during the twentieth century. Only significant relations are reported based on the Pearson correlation coefficient.

For most traits, no correlation between variety registration date and trait values was found (Supplementary Figures 20, 21). However, some trends were observed for traits related to root depth in bread wheat (Figure 8). Root angle, depth, and CGY values increased steadily in varieties registered between 1940 and 2010 (*p*-value = 0.07 and 0.08, respectively). This was confirmed by the significant increase observed for Depth_80 (*p*-value = 0.03) over time. Such trends were not observed in durum wheat. In bread wheat, the mean number of seminal roots has decreased significantly (*p*-value = 0.0002) from 4.5 in varieties registered before 1940 to 4.2 for the newest cultivars. In durum wheat, the same phenomenon occurred but not to a significant extent (*p*-value = 0.09). From 1940 to 2015, in bread wheat, newly registered cultivars had more tillers going from 2.4 tillers before 1960 to 2.75 for the most recent cultivars (*p*-value = 0.004). The opposite was observed in durum wheat (*p*-value = 0.03, Figure 9) with a decrease from 2.75 tillers for varieties registered in 1970 to 2.25 in 2015. A slight decrease in root and above-ground biomass was also detected in both species (*p* < 0.05). Finally, in durum wheat, a significant increase in the root-shoot ratio was observed (*p* = 0.012) over the registration period studied.

## Discussion

In this study, we characterized root traits on two bread wheat panels and one durum wheat panel grown in controlled conditions in three successive experiments, to investigate whether root traits measured in platforms can be related to field performances and to what extent any relationship at the trait or at the QTL level is environment-specific.

### Genetic Variability and Correlation Between Root System Architecture Traits

Heritabilities of the RSA traits were intermediate to high (Table 1) and similar to heritabilities observed in comparable studies, e.g., H^2^ = 0.73 for root angle and H^2^ = 0.67 for the number of roots in Canè et al. (2014). Higher heritability of root biomass (H^2^ = 0.75) and seminal root angle (H^2^ = 0.85) has been reported in an experiment with six independent replicates (Beyer et al., 2019). In our study, each non-check variety was repeated six times, but only in three independent rhizotubes and this was apparently not sufficient to reach such high heritabilities. In addition, heritabilities are highly dependent on the genetic variance of the population studied, and some studies with higher values for heritability were based on inter-specific populations, which resulted in higher phenotypic variability (Xie et al., 2017). Phenotypic variability was intentionally maximized when defining the durum wheat panel, which is partly composed of lines derived from crosses with wild relatives. This translated to higher genetic variances and heritabilities for several traits (Table 1), despite the panel being much smaller than the bread wheat panel. In summary, our experimental setup enables us to scan a large part of the genetic and phenotypic variability associated with studied traits in wheat species.

The range of RSA trait values measured in our study (Table 2 and Supplementary Figure 1) are also coherent with the literature for seminal root angle, the number of roots (Canè et al., 2014; Adeleke et al., 2020), and root biomass (Beyer et al., 2019). RSA traits were correlated in our case, which is in line with reports of high correlations between wheat root seedling traits (Bai et al., 2013; Atkinson et al., 2015; Kabir et al., 2015; Beyer et al., 2019; Adeleke et al., 2020). This might be attributed to high phenotypic variability in inter-species or inter-population comparisons (Kabir et al., 2015; Adeleke et al., 2020). Comparison of the less diverse population might yield lower correlations. Indeed, if we merge the elite and the diversity panels for both species, the correlation between traits increases. Thus, some trait correlations can be seen at a larger scale but not at the intraspecific scale. The weakness of the correlations indicates that the traits we measured might capture different independent features of RSA. This is confirmed by the detection of distinct genetic determinism (QTLs) for each trait.

Consistent correlations were observed between aerial biomass and root biomass (*r* > 0.75 in all panels in both species) and between aerial biomass and the root-shoot ratio. This might reflect developmental constraints and the trade-off in carbon allocation to the different organs of the plants. This interpretation is borne out by the narrower genetic variability of the observed root-shoot ratio compared to other traits.

### Trends in Root Traits in Cultivars Registered Between 1940 and 2010

Two groups of traits have been incorporated into wheat developed during the period from 1940 to 2010. First, the number of tillers increased in both species (Figures 8, 9, and Supplementary Figures 20, 21). In bread wheat, this was accompanied by a decrease in the number of seminal roots (Figure 8). During seedling development, seminal roots develop first, then tillers develop from axillary buds either from the coleoptile or leaves (Klepper et al., 1982). As we did not observe a significant increase in the number of leaves with respect to the cultivar registration year, we may assume that either slightly more buds develop in more recent cultivars or that buds develop more rapidly. In our experiment, plants had three, five, or seven seminal roots. More rapid bud development associated with an increase in tiller number might explain why there are fewer seminal roots if faster tiller bud growth prevents the development of the last pair of seminal roots. Not all tillers go on to carry an ear at harvest, but the data may reflect the genetic progress reported for the number of ears m^–2^ in several studies (Austin et al., 1989; Green et al., 2012; Sanchez-Garcia et al., 2013).

A significant reduction in shoot and root biomass has been bred into cultivars of both species between 1940 and 2010 (Figures 8, 9). This was accompanied by a deepening of the root system during the same period in bread wheat only (Figure 8). Zhu et al. (2019) also observed that very recent cultivars (registered after 2008) have deeper roots than old cultivars registered before 1900. Aziz et al. (2017) reported that in Australian wheat root and above-ground biomass at the booting stage has been reduced. This can be interpreted in light of the group selection theory (Denison et al., 2003). With less competition between plants due to reduced vegetative biomass, narrow root angle, and a deep root system (Nakhforoosh et al., 2021) the stand performance is maximized (Donald, 1968). Much of modern breeding can indeed be interpreted as an endeavor to reduce the size of organs promoting competition (roots, plant heights) to maximize the performance in the fields rather than the performance of individual plants. This type of evolution has been reported during the domestication process over a longer time scale (Nakhforoosh et al., 2021).

In general, there is no consensus on whether root traits were indirectly selected or not (Hurd, 1974; Lupton et al., 1974; Cholick et al., 1977; Chloupek et al., 2006; Aziz et al., 2017). One reason may be due to the size of sample errors as most of the studies are based on only a few genotypes (<20), insufficient to represent large periods of history (between 50 and more than 100 years). Another reason may be the high genotype × environment × management (G × E × M) interactions resulting in different adaptive values of root traits depending on the breeding environments.

In addition to these historic trends, it would be interesting to look at geographic patterns related to root traits. We can indeed expect that breeding has contributed to adapting root traits to local pedoclimatic constraints. This could for instance be used to determine local ideotype adapted to future climatic conditions.

### Correlation Between High-Throughput Phenotyping Platform and Agronomic Field Performances

In several papers, inconsistent relationships between root traits and agronomic performances across years and environments were reported (Canè et al., 2014; Xie et al., 2017; Roselló et al., 2019; Rich et al., 2020). Our results are in line with this observation, especially in the most phenotypically diverse panel (BW_div, Figures 2–4). This suggests that the adaptive value of RSA varies from one environment to another. Indeed, environments where root depth was adaptive (high correlation between root depth and yield or agronomic value) were distinct from those where root width was adaptive (high correlation between root width and yield or agronomic value). All of this suggests that the relation between RSA and yield depends on the characteristics of the environment.

The adaptive value of specific root traits is known in Australian environments with specific stress patterns. For instance, in the north-eastern wheat belt of Australia, where crops rely on stored soil moisture to complete the whole cycle and with a high risk of water shortage during grain filling, the optimal strategy was to manage the available water in the soil reserve during the crop cycle by capturing less water at early stages of development, keeping resources in reserve for the grain-filling stage (Passioura, 1972; Dreccer et al., 2000; Manschadi et al., 2006). In Mediterranean-like environments of the south western part of Australia, where crops rely mainly on seasonal rainfall, increasing root biomass, root length density, and root volume might increase early vigor, pre-anthesis water use, and crop performances (Rebetzke and Richards, 1999; Manschadi et al., 2006). Contrasted performances between the wheat of these two regions are mainly driven by the adaptation of cultivars to the water stress pattern whereas trials of our multi-environment trials are mostly located in France with restricted climatic variations. Thus, many stresses each explaining a small proportion of performance variability and the adaptive value of a particular RSA might be less visible in our experiment. Understanding the adaptive value of a particular trait regarding stress requires a fine characterization of the resources of the environment and thus the measurement of many environmental variables. Here we evaluate whether, according to the results of the multi-environment trials, environmental variables describing a stress pattern can explain the inconsistency of the relationships between root traits and agronomic performances measured in the fields, as we hypothesize.

#### At the Trait Level

First, in the BW_div panel, despite the many significant correlations observed between root traits and agronomic variables, we could not test our hypothesis as we did not have information on the environmental variables. Thus we can only speculate that the relationship between root depth and GYC in three environments (17INRmon_NUE_HN, 17SYNmoi_NUE_HN, and 17SYNmoi_NUE_LN) might be due to cultivars with deep roots gaining extra access to nutrients and water, resulting in higher yield, especially in water-scarce environments and nutrient-poor environments (Palta et al., 2011; Robinson et al., 2018; Nakhforoosh et al., 2021). In the same vein, we speculate that observed relationships between root biomass and TKW (and TKC) and between root-shoot ratio and GPD in the 17 BAY_mil_SEC environments might be explained by access to extra resources during the grain-filling period. Indeed, GPD and TKW are both determined after flowering and are dependent on water and nitrogen uptake in the soil. It has indeed specifically been shown that GPD is correlated to post-flowering nitrogen absorption in a trial network set up in Northern France (Bogard et al., 2010).

Second, in BD_EPO, some environmental characterizations were available, especially regarding water stress. We found two elements confirming our hypothesis. First, we observed better performances in durum varieties with high root-shoot ratios in QUALPREST_2019 and INRAE_2019_opt environments. The common features of these two environments were deep soil and the strongest water stress of the multi-environment trial. Specifically, the water stress index dropped early at tillering down to 0.72 compared to 0.9 for the other environments. Thus, for equal above-ground biomass, plants with higher root biomass were able to capture more water at depth and thus avoid yield loss due to stress.

Third, using multi-trait approaches to better describe RSA, we found that the relationship between root traits and GYC was more important in water-deficient environments where roots are likely to play a major role in water uptake, especially in the most stressed environments (e.g., INRA_2019_sec, INRA_2019_opt).

Lastly, in the BW_elit panel, environmental characterization was precise (Rincent et al., 2019) but no correlation was found between agronomic performances and root traits, likely due to the narrow range of phenotypic variation compared with the other panels.

#### At the Allele Level

We found numerous RSA QTLs that co-localize with agronomic performance QTLs. Assuming that the SNPs responsible for the variation in the RSA QTLs and the variation in agronomic performances are the same, some RSA QTLs have contrasting effects depending on the level of stress according to environmental covariates. If relationship patterns can therefore be explained at both the phenotypic and allelic level, it follows that alleles conferring specific RSA might be suboptimal in some environments and optimal in others. If so, it would be advisable for future breeding programs on root traits to carefully consider the characteristics of the target environments.

Two types of cases were identified regarding the relation between RSA QTL effects on agronomic variables and environmental variables.

First, we found some quantitative trait alleles (QTA) whose effects vary linearly with environmental variables. For instance, the QTA increasing root depth increases yield in stressed environments. Deeper roots might enable plants to access water from deeper layers and have been associated with high yield in some environments (Bai et al., 2019). The QTA allele decreasing both root and above-ground biomass (Supplementary Table 5, marker AX-89687612) was detrimental to yield only in a radiation-poor environment, and indeed low aerial biomass is expected to be detrimental in such an environment.

Second, some QTA had a significant effect on an agronomic variable only when the environmental variable reached a certain threshold. For instance, the QTA allele increasing root angle (Supplementary Table 5, marker AX-89517948) had a negative effect on TKC only in the environment AVRgre2014sec, by far the most stressed environment of the multi-environment trial. No effect was observed in less stressed environments. Wide RSA might diminish access to deep soil layers and increase competition between plants for resources and in particular water. This may have a particularly detrimental effect for TKW in water-stressed environments when the stress occurs around meiosis, for example, by inducing early senescence of the flag leaves, which is normally photosynthetically active, and so limiting the resources for grain-filling.

Thus, environmental variables describing a stress pattern can be used to decipher the complex relationships between root traits and agronomic performance. This was demonstrated especially for traits related to root depth, root-shoot ratio, and root biomass. Given that the same root allele or root trait can have a beneficial effect on agronomic performance in one environment and a detrimental effect in another, root ideotyping is an essential step for any breeding program before performing any selection.

### Future Application of Platform-Phenotyping

The use of HTPP, such as the 4PMI platform, is still in its infancy. The limitations of HTPP are acknowledged, such as the lack of correlation between RSA in HTPP and RSA in the field (Watt et al., 2013; Bai et al., 2019), the lack of correlation between early RSA and mature RSA even in the same environment (Lynch and Brown, 2012; Watt et al., 2013), and the high influence of genotype × environment interactions on root variability (Botwright Acuña and Wade, 2012). We have nevertheless demonstrated that it is still possible to identify interesting links between traits measured in HTPP and field performances at both the trait and allelic level. Distinct QTLs and genetic determinism were found for both bread and durum wheat and for all traits, indicating that the HTPP measurements captured independent features of the RSA. With continued developments in image analysis, the 4PMI platform will be able to scan many other root traits that might be useful to breeders. Particularly, dynamic traits measured over time and derived traits from response curves to major environmental factors would be meaningful for studying RSA and detecting genotypes that are more adapted to global change. The first dynamic wheat mapping studies were conducted on aerial traits using sampled time points either in controlled conditions (Camargo et al., 2018) or in the field (Lyra et al., 2020), leading to the identification of QTLs that were persistent over the growth period and transient QTLs, both types being potential new targets for breeding. In addition, dynamic phenotyping would make it easier to compare RSA traits of cultivars with very different phenology. For instance, there is a general trend for the root-shoot ratio to decrease over time, as was shown for 17 eudicot species by Mašková and Herben (2018). When properly calibrated, high-throughput imaging can estimate biomasses non-destructively (Tracy et al., 2020). at the same developmental stage (based on the number of leaves of the main stem) or at the same shoot biomass, thus allowing comparison of root traits on very different accessions with less bias linked to phenology.

## Conclusion

Our study has shown that root traits measured in a platform are moderately heritable in the three studied panels of bread and durum wheat. We confirmed that the relationship between the root traits measured in controlled conditions at an early stage and traits measured in fields is highly dependent on the environmental conditions experienced in the fields. We used environmental variables to explain the variability of the relationship between root traits and agronomic performances. This relation holds true at both the trait and the allelic level.

At the trait level, the geometry of RSA (wide or deep) enables adaptation to distinct environments in bread wheat. In durum wheat, the relationships between root traits (particularly root-shoot ratio) and agronomic variables (particularly GYC) were stronger in water stress.

At the allelic level, we found numerous root QTLs in both species. Some QTLs had an effect on agronomic performances varying linearly with environmental variables whereas others had an effect on agronomic performances only when the environmental variable reached a specific threshold.

This highlights that multi-environment trials are required to evaluate the agronomic value of root phenotypes measured in HTPP and that breeders should prioritize root ideotyping for target environments.

## Data Availability Statement

The original contributions presented in the study are included in the article/Supplementary Material, further inquiries can be directed to the corresponding author.

## Author Contributions

MC, RR, and PR analyzed the data. PR, CS, CJ, ML, WN, BD, VA, JL, and RR participated in the 4PMI experiments. CS, CJ, and RR designed the 4PMI experiments. ML analyzed the 4PMI images. TBWC provided the genotyping and field phenotyping data. PR, CS, CJ, ML, SL, KB, JL, and RR initiated the project. RR supervised the project and designed the experiments. MC wrote the manuscript under the supervision of RR, JL, VA, and PR. All authors reviewed the manuscript.

## Conflict of Interest

SL, PD, and A-VD were employed by Biogemma Limagrain. The remaining authors declare that the research was conducted in the absence of any commercial or financial relationships that could be construed as a potential conflict of interest.

## Publisher’s Note

All claims expressed in this article are solely those of the authors and do not necessarily represent those of their affiliated organizations, or those of the publisher, the editors and the reviewers. Any product that may be evaluated in this article, or claim that may be made by its manufacturer, is not guaranteed or endorsed by the publisher.

## Footnotes

1
http://faostat.fao.org/
