# Supplementary material for: Genetic Analysis of Platform-Phenotyped Root System Architecture of Bread and Durum Wheat in Relation to Agronomic Traits
Source: Front Plant Sci. 2022 Mar 25;13:853601. doi: 10.3389/fpls.2022.853601 (PMC8992431; doi:10.3389/fpls.2022.853601)
Supplement: Supplementary file 1 [file Data_Sheet_1.PDF]

Table of Contents

List of figures annexe	2
Annex Figure 1	3
Annex Figure 2	4
Annex Figure 3	5
Annex Figure 4	6
Annex Figure 5	7
Annex Figure 6	8
Annex Figure 7	9
Annex Figure 8	10
Annex Figure 9	11
Annex Figure 10	12
Annex Figure 11	13
Annex Figure 12	14
Annex Figure 13	15
Annex Figure 14	16
Annex Figure 15	17
Annex Figure 16	18
Annex Figure 17	58
Annex Figure 18	59
Annex Figure 19	60
Annex Figure 20	61
Annex Figure 21	62

Annex Figure 1: Relation between Grain Yield (GY) and heading date (DOE) in the bread wheat diversity panel

Annex Figure 2: Relation between Grain Yield (GY) and heading date (DOE) in the bread wheat elite panel

Annex Figure 3: Relation between Grain Yield (GY) and heading date (DOE) in the durum wheat panel

Annex Figure 4: Histogram of root traits in the different panels

Annex Figure 5: Correlation between platform traits in bread wheat panels (upper triangle: diversity panel, lower triangle Elite panel).

Annex Figure 6: Correlation between platform traits in bread wheat panels (upper triangle: EPO panel, lower triangle Elite panel).

Annex Figure 7: Boxplot of correlation coefficients between platform traits and agronomic values measured in fields in the bread wheat elite panel. Each point of a boxplot represents one of the 42 environments. DOE: Heading date, GYC, GNC, TKC: residuals of the linear regression of DOE on grain yield (GY), grain number (GN), thousand kernel weight (TKW).

Annex Figure 8: Boxplot of correlation coefficients between platform traits and agronomic values measured in fields in the bread wheat diversity panel. Each point of a boxplot represents one of the 17 environments. DOE: Heading date, GYC, GNC, TKC: residuals of the linear regression of DOE on grain yield (GY), grain number (GN), thousand kernel weight (TKW).

Annex Figure 9: Boxplot of correlation coefficients between platform traits and agronomic values measured in fields in the durum wheat panel. Each point of a boxplot represents one of the 10 environments. DOE: Heading date, GYC, GNC, TKC: residuals of the linear regression of DOE on grain yield (GY), grain number (GN), thousand kernel weight (TKW).

Annex Figure 10: Optimal number of clusters for durum wheat using the Elbow method

Annex Figure 11: Optimal number of clusters for bread wheat using the Elbow method

Annex Figure 12: Results of a PCA analysis on root traits. Aerial traits are not included in the computation of cultivar coordinates. Groups were computed based on the kmeans method using the elbow method to choose the optimal number of groups.

Annex Figure 13: QQplot of GWAS of root traits for bread wheat

Annex Figure 14: Manhattan plot of root traits for bread wheat. Significant SNP with FDR thresholds at 10% are colored in blue. Red lines indicate the Bonferonni threshold.

Annex Figure 15: QTL plot of bread wheat. Each point represents the position of a QTL. QTL of agronomic traits are plotted only when they co-localize with a platform QTL. For agronomic QTL, each line represents an environment. RLR2 represents the proportion of variance explained by the QTL.

Annex Figure 16: Allelic effect of root traits QTLs detected on agronomic variable in several environments as a function of environmental variable measured in these same environments. Acronym of environmental variable are reported in (Rincent et al. 2019).

Annex Figure 17: QQplot of GWAS of root traits for durum wheat

Annex Figure 18: Manhattan plot of root traits for durum wheat. Significant SNP with FDR thresholds at 10% are colored in blue. Red lines indicate the Bonferonni threshold.

Annex Figure 19: QTL plot of durum wheat. Each point represents the position of a QTL. QTL of agronomic traits are plotted only when they co-localize with a platform QTL. For agronomic QTL each line represents an environment. RLR2 represents the proportion of variance explained by the QTL.

Annex Figure 20: Evolution of bread wheat platform traits during the XXth century. Pearson correlation coefficient and associated p-value are reported.

Annex Figure 21: Evolution of durum wheat platform traits during the XXth century. Pearson correlation coefficient and associated p-value are reported.

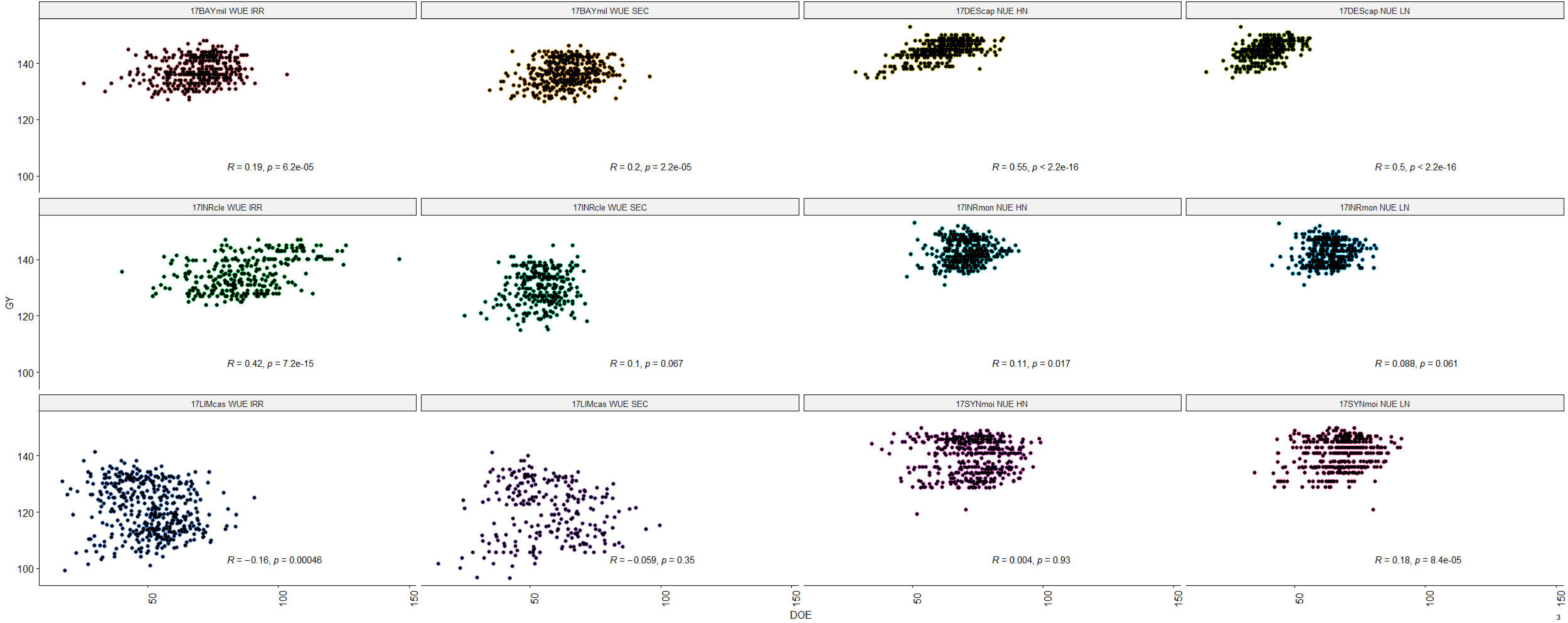

17BAYmil WUE IRR
17BAYmil WUE SEC
17DEScap NUE HN
17DEScap NUE LN
R = 0.19, p = 6.2e-05
R = 0.2, p = 2.2e-05
R = 0.55, p < 2.2e-16
R = 0.5, p < 2.2e-16
17INRcle WUE IRR
17INRcle WUE SEC
17INRmon NUE HN
17INRmon NUE LN
R = 0.42, p = 7.2e-15
R = 0.1, p = 0.067
R = 0.11, p = 0.017
R = 0.088, p = 0.061
17LIMcas WUE IRR
17LIMcas WUE SEC
17SYNmoi NUE HN
17SYNmoi NUE LN
R = −0.16, p = 0.00046
R = −0.059, p = 0.35
R = 0.004, p = 0.93
R = 0.18, p = 8.4e-05
GY
DOE

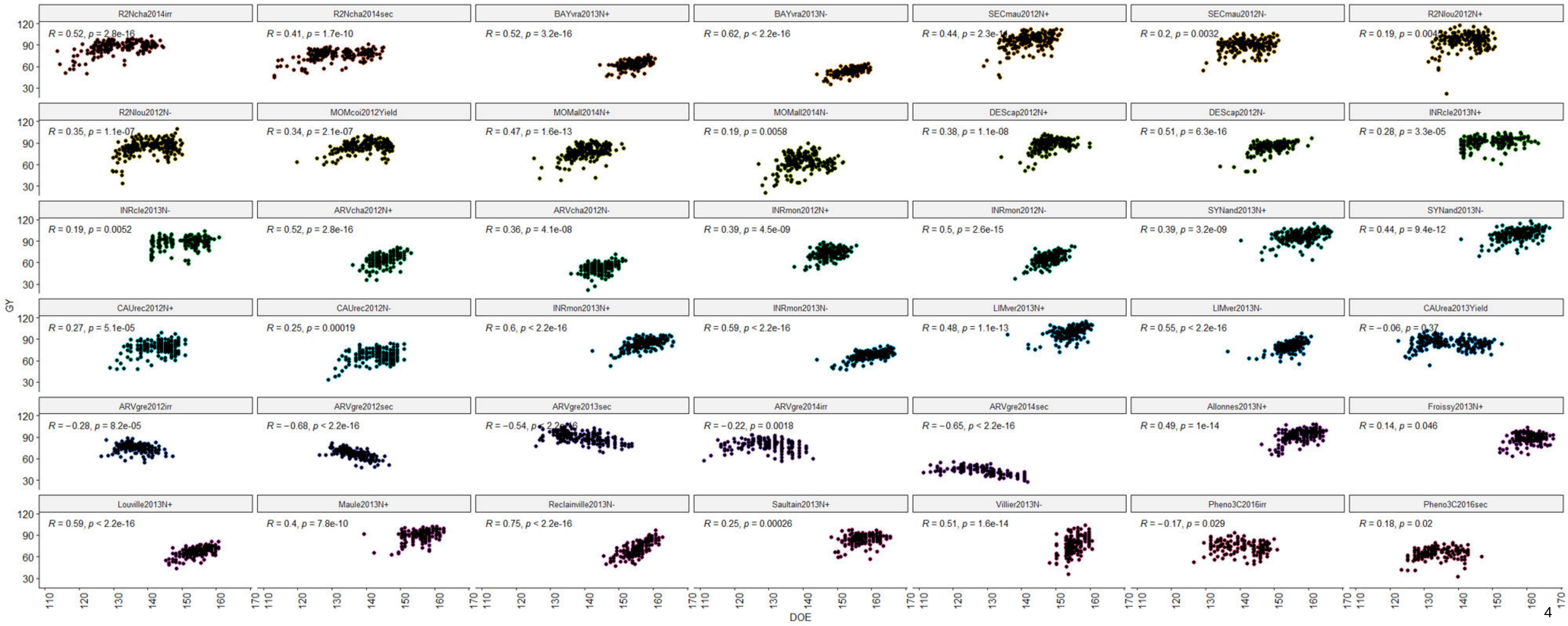
GY
DOE
R2Ncha2014irr
R = 0.52, p = 2.8e-16
R2Ncha2014sec
R = 0.41, p = 1.7e-10
BAYvra2013N+
R = 0.52, p = 3.2e-16
BAYvra2013N-
R = 0.62, p < 2.2e-16
SECmau2012N+
SECmau2012N-
R = 0.2, p = 0.0032
R2Nlou2012N+
R2Nlou2012N-
R = 0.35, p = 1.1e-07
MOMcoi2012Yield
R = 0.34, p = 2.1e-07
MOMall2014N+
R = 0.47, p = 1.6e-13
MOMall2014N-
R = 0.19, p = 0.0058
DEScap2012N+
R = 0.38, p = 1.1e-08
DEScap2012N-
R = 0.51, p = 6.3e-16
INRcle2013N+
R = 0.28, p = 3.3e-05
INRcle2013N-
R = 0.19, p = 0.0052
ARVcha2012N+
R = 0.52, p = 2.8e-16
ARVcha2012N-
R = 0.36, p = 4.1e-08
INRmon2012N+
R = 0.39, p = 4.5e-09
INRmon2012N-
R = 0.5, p = 2.6e-15
SYNand2013N+
R = 0.39, p = 3.2e-09
SYNand2013N-
R = 0.44, p = 9.4e-12
CAUrec2012N+
R = 0.27, p = 5.1e-05
CAUrec2012N-
R = 0.25, p = 0.00019
INRmon2013N+
R = 0.6, p < 2.2e-16
INRmon2013N-
R = 0.59, p < 2.2e-16
LIMver2013N+
R = 0.48, p = 1.1e-13
LIMver2013N-
R = 0.55, p < 2.2e-16
CAUrea2013Yield
R = −0.06, p = 0.37
ARVgre2012irr
R = −0.28, p = 8.2e-05
ARVgre2012sec
R = −0.68, p < 2.2e-16
ARVgre2013sec
ARVgre2014irr
R = −0.22, p = 0.0018
ARVgre2014sec
R = −0.65, p < 2.2e-16
Allonnes2013N+
R = 0.49, p = 1e-14
Froissy2013N+
R = 0.14, p = 0.046
Louville2013N+
R = 0.59, p < 2.2e-16
Maule2013N+
R = 0.4, p = 7.8e-10
Reclainville2013N-
R = 0.75, p < 2.2e-16
Saultain2013N+
R = 0.25, p = 0.00026
Villier2013N-
R = 0.51, p = 1.6e-14
Pheno3C2016irr
R = −0.17, p = 0.029
Pheno3C2016sec
R = 0.18, p = 0.02
30
60
90
120
110
120
130
140
150
160
170

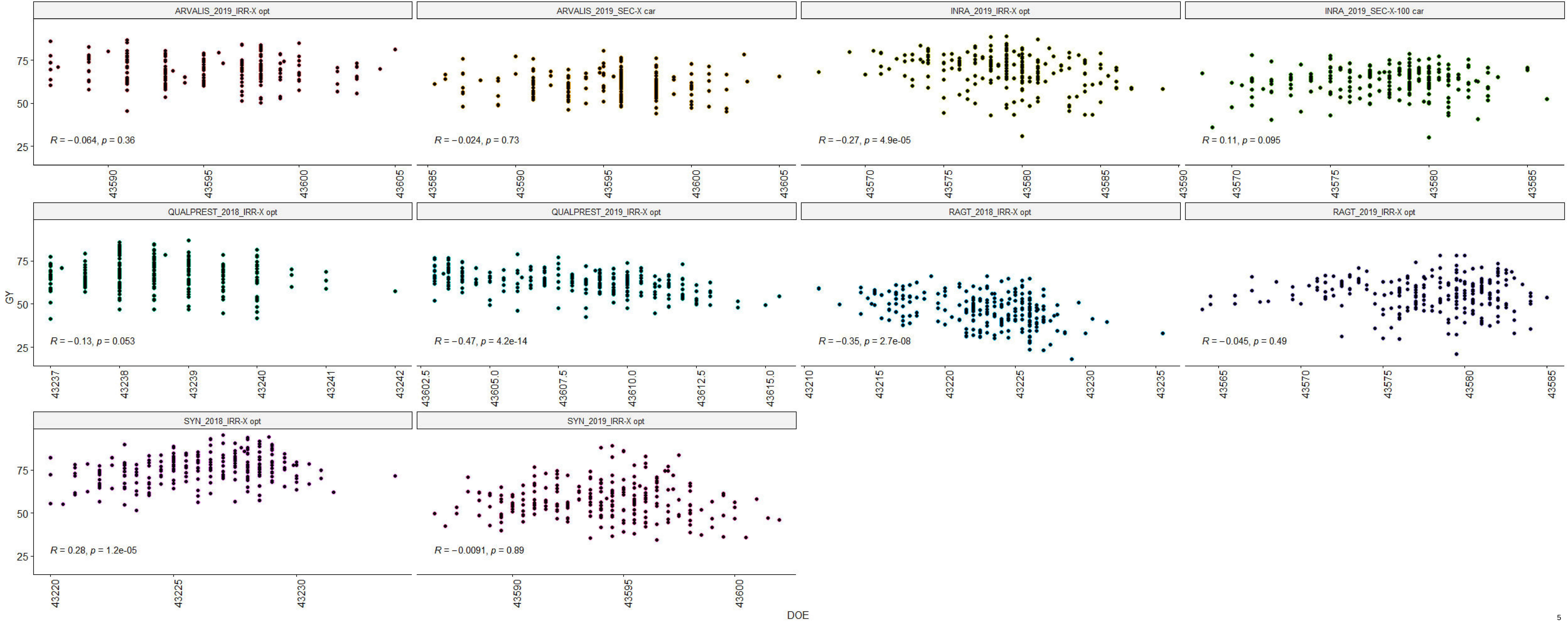
ARVALIS_2019_IRR-X opt
R = −0.064, p = 0.36
ARVALIS_2019_SEC-X car
R = −0.024, p = 0.73
INRA_2019_IRR-X opt
R = −0.27, p = 4.9e-05
INRA_2019_SEC-X-100 car
R = 0.11, p = 0.095
QUALPREST_2018_IRR-X opt
R = −0.13, p = 0.053
QUALPREST_2019_IRR-X opt
R = −0.47, p = 4.2e-14
RAGT_2018_IRR-X opt
R = −0.35, p = 2.7e-08
RAGT_2019_IRR-X opt
R = −0.045, p = 0.49
SYN_2018_IRR-X opt
R = 0.28, p = 1.2e-05
SYN_2019_IRR-X opt
R = −0.0091, p = 0.89
GY
DOE

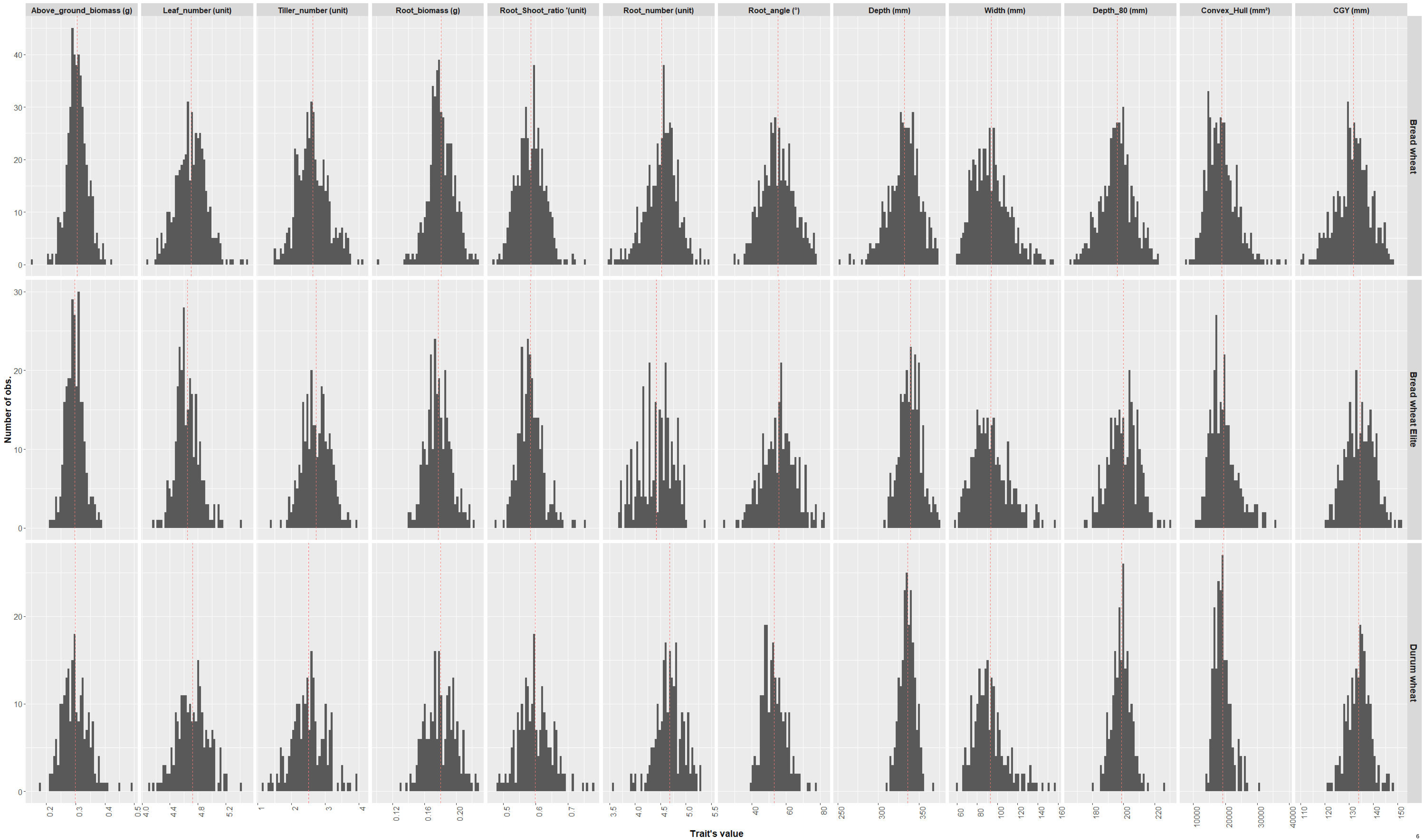
Above_ground_biomass (g)
Leaf_number (unit)
Tiller_number (unit)
Root_biomass (g)
Root_Shoot_ratio '(unit)
Root_number (unit)
Root_angle (°)
Depth (mm)
Width (mm)
Depth_80 (mm)
Convex_Hull (mm²)
CGY (mm)
Bread wheat
Bread wheat Elite
Durum wheat
Number of obs.
Trait's value

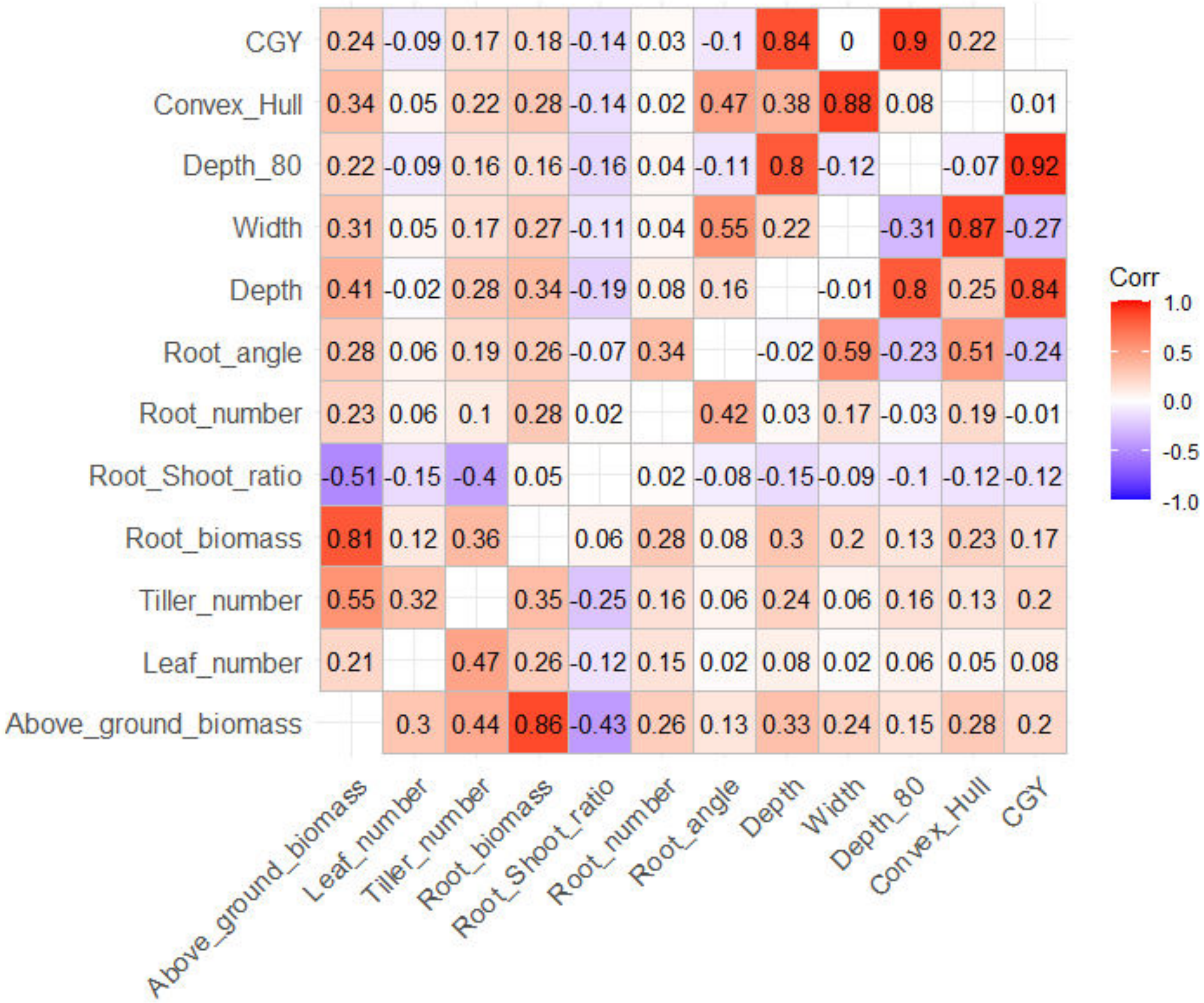
CGY
Convex_Hull
Depth_80
Width
Depth
Root_angle
Root_number
Root_Shoot_ratio
Root_biomass
Tiller_number
Leaf_number
Above_ground_biomass
0.24 -0.09 0.17 0.18 -0.14 0.03 -0.1 0.84 0 0.9 0.22
0.34 0.05 0.22 0.28 -0.14 0.02 0.47 0.38 0.88 0.08 0.01
0.22 -0.09 0.16 0.16 -0.16 0.04 -0.11 0.8 -0.12 -0.07 0.92
0.31 0.05 0.17 0.27 -0.11 0.04 0.55 0.22 -0.31 0.87 -0.27
0.41 -0.02 0.28 0.34 -0.19 0.08 0.16 -0.01 0.8 0.25 0.84
0.28 0.06 0.19 0.26 -0.07 0.34 -0.02 0.59 -0.23 0.51 -0.24
0.23 0.06 0.1 0.28 0.02 0.42 0.03 0.17 -0.03 0.19 -0.01
-0.51 -0.15 -0.4 0.05 0.02 -0.08 -0.15 -0.09 -0.1 -0.12 -0.12
0.81 0.12 0.36 0.06 0.28 0.08 0.3 0.2 0.13 0.23 0.17
0.55 0.32 0.35 -0.25 0.16 0.06 0.24 0.06 0.16 0.13 0.2
0.21 0.47 0.26 -0.12 0.15 0.02 0.08 0.02 0.06 0.05 0.08
0.3 0.44 0.86 -0.43 0.26 0.13 0.33 0.24 0.15 0.28 0.2
Above_ground_biomass
Leaf_number
Tiller_number
Root_biomass
Root_Shoot_ratio
Root_number
Root_angle
Depth
Width
Depth_80
Convex_Hull
CGY
Corr
1.0
0.5
0.0
-0.5
-1.0

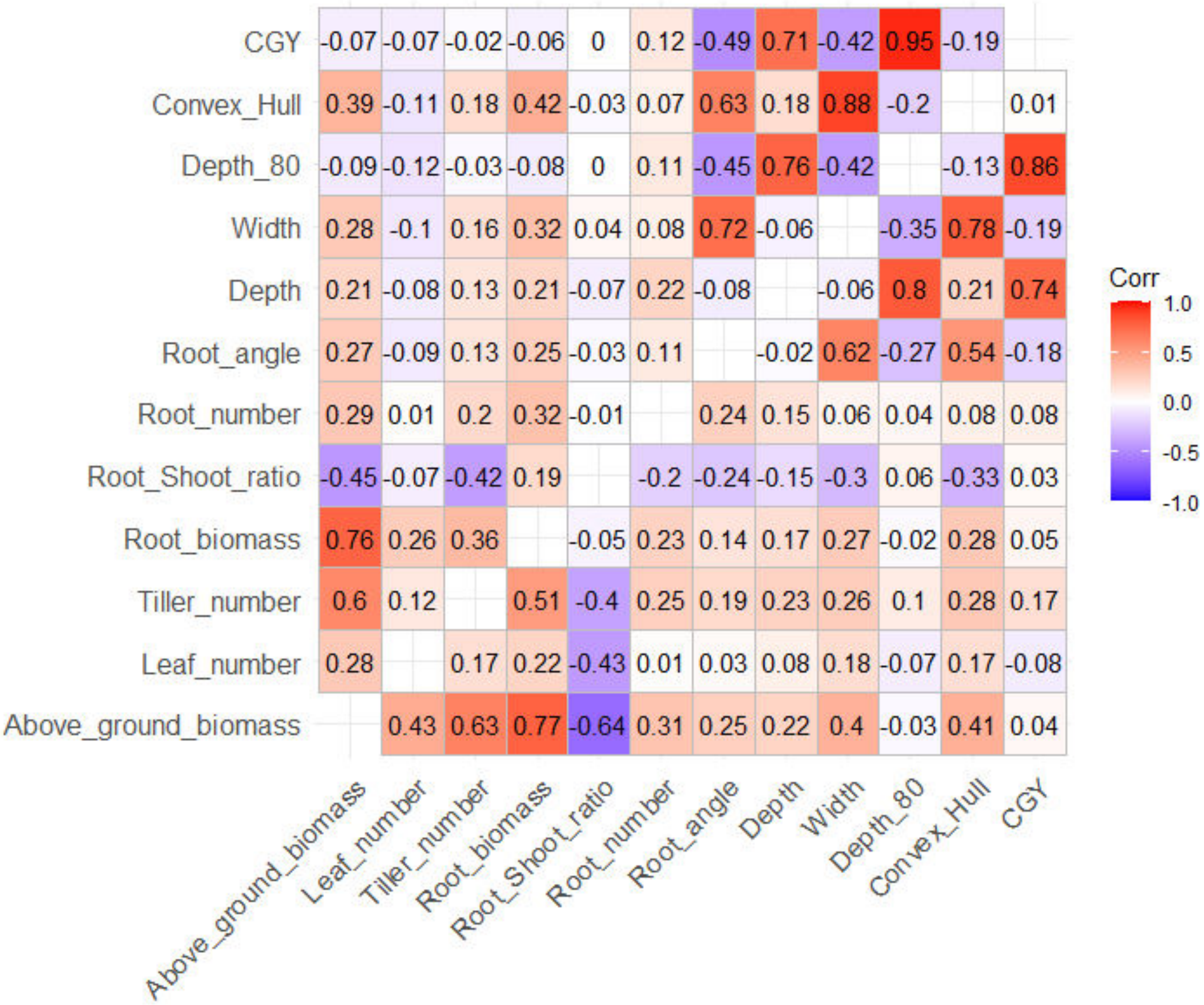
CGY
Convex_Hull
Depth_80
Width
Depth
Root_angle
Root_number
Root_Shoot_ratio
Root_biomass
Tiller_number
Leaf_number
Above_ground_biomass
Corr
1.0
0.5
0.0
-0.5
-1.0

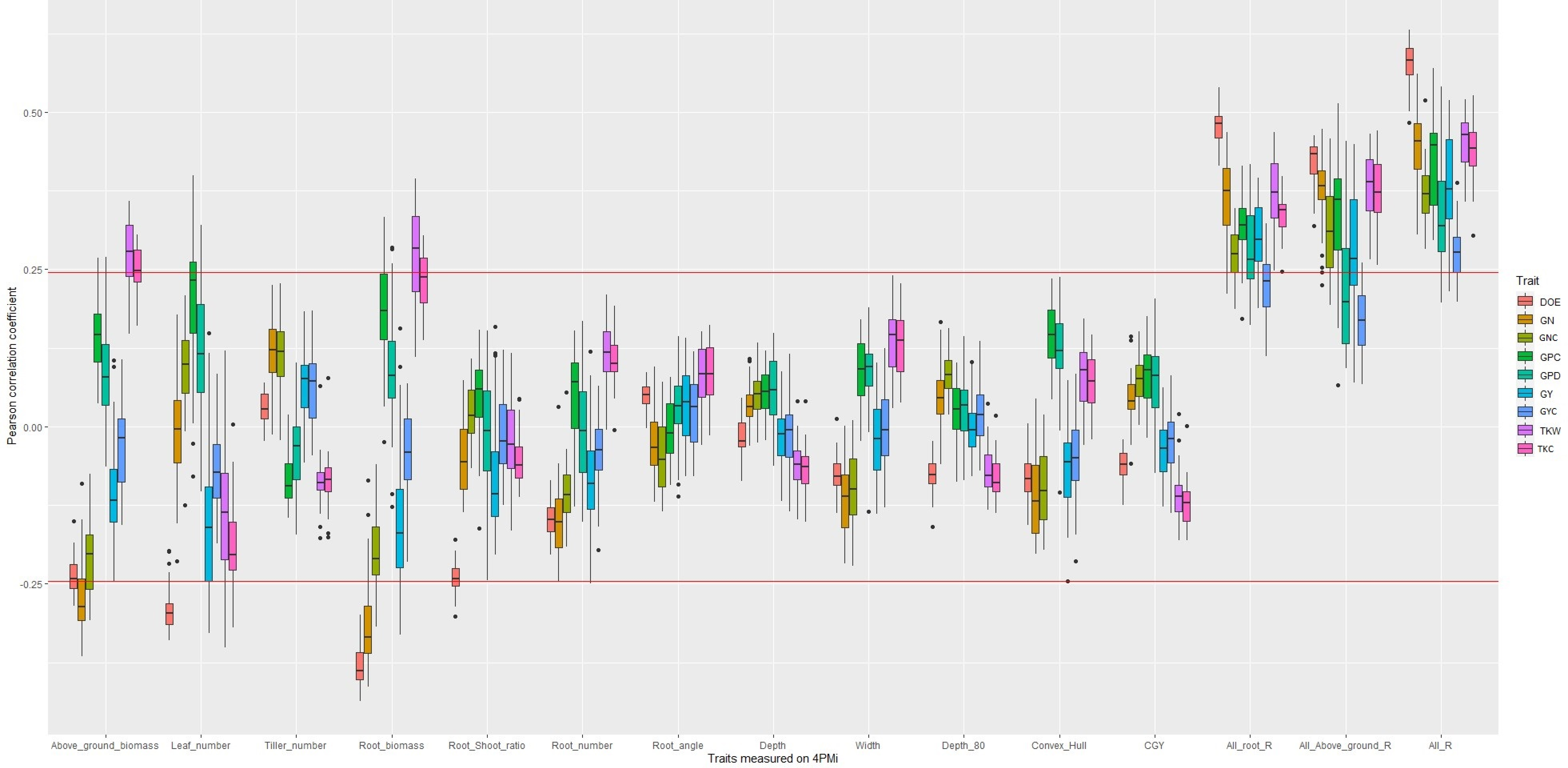

Pearson correlation coefficient
0.50
0.25
0.00
-0.25
Above_ground_biomass
Leaf_number
Tiller_number
Root_biomass
Root_Shoot_ratio
Root_number
Root_angle
Depth
Width
Depth_80
Convex_Hull
CGY
All_root_R
All_Above_ground_R
All_R
Traits measured on 4PMi
Trait
DOE
GN
GNC
GPC
GPD
GY
GYC
TKW
TKC

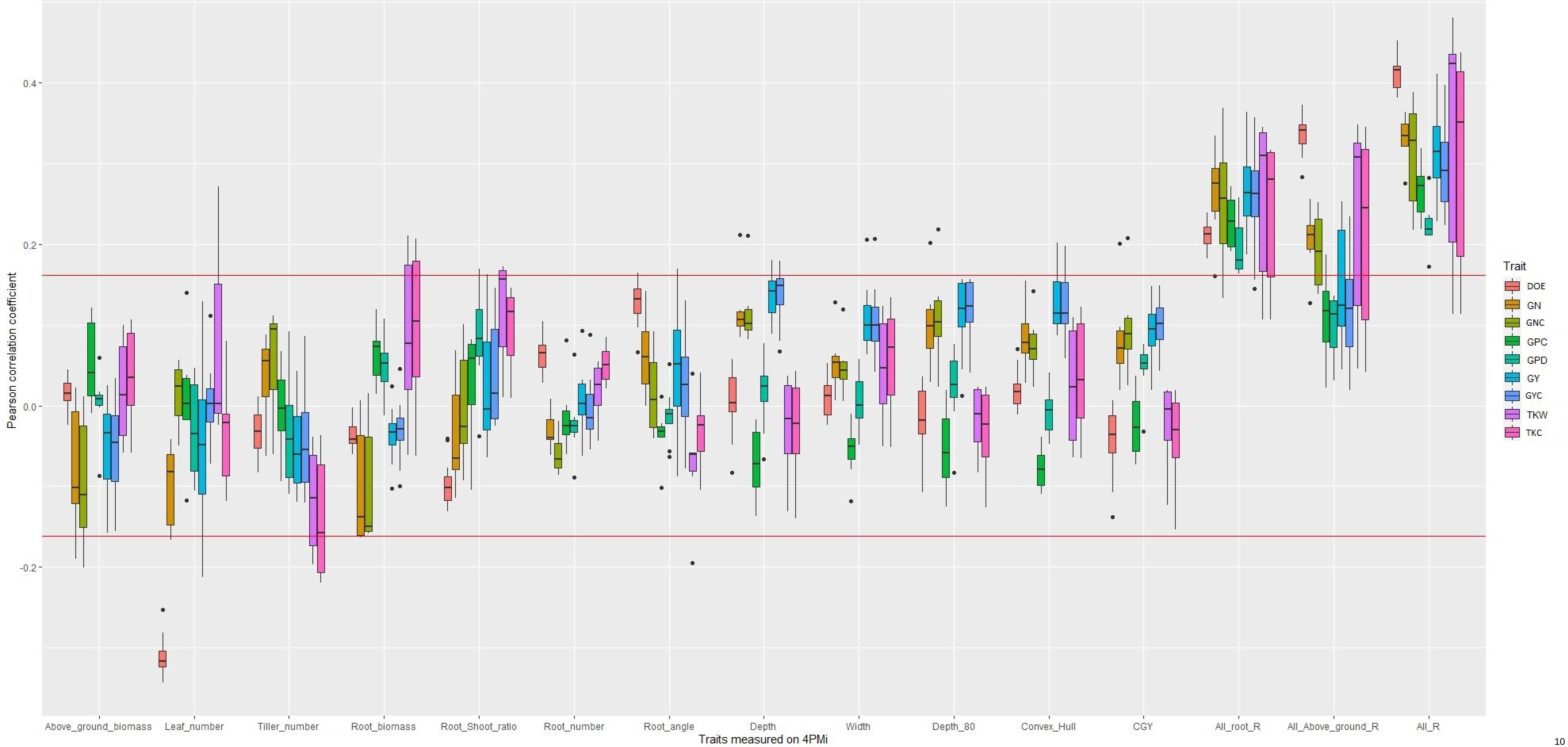

Pearson correlation coefficient
0.4
0.2
0.0
-0.2
Above_ground_biomass
Leaf_number
Tiller_number
Root_biomass
Root_Shoot_ratio
Root_number
Root_angle
Depth
Width
Depth_80
Convex_Hull
CGY
All_root_R
All_Above_ground_R
All_R
Traits measured on 4PMi
Trait
DOE
GN
GNC
GPC
GPD
GY
GYC
TKW
TKC

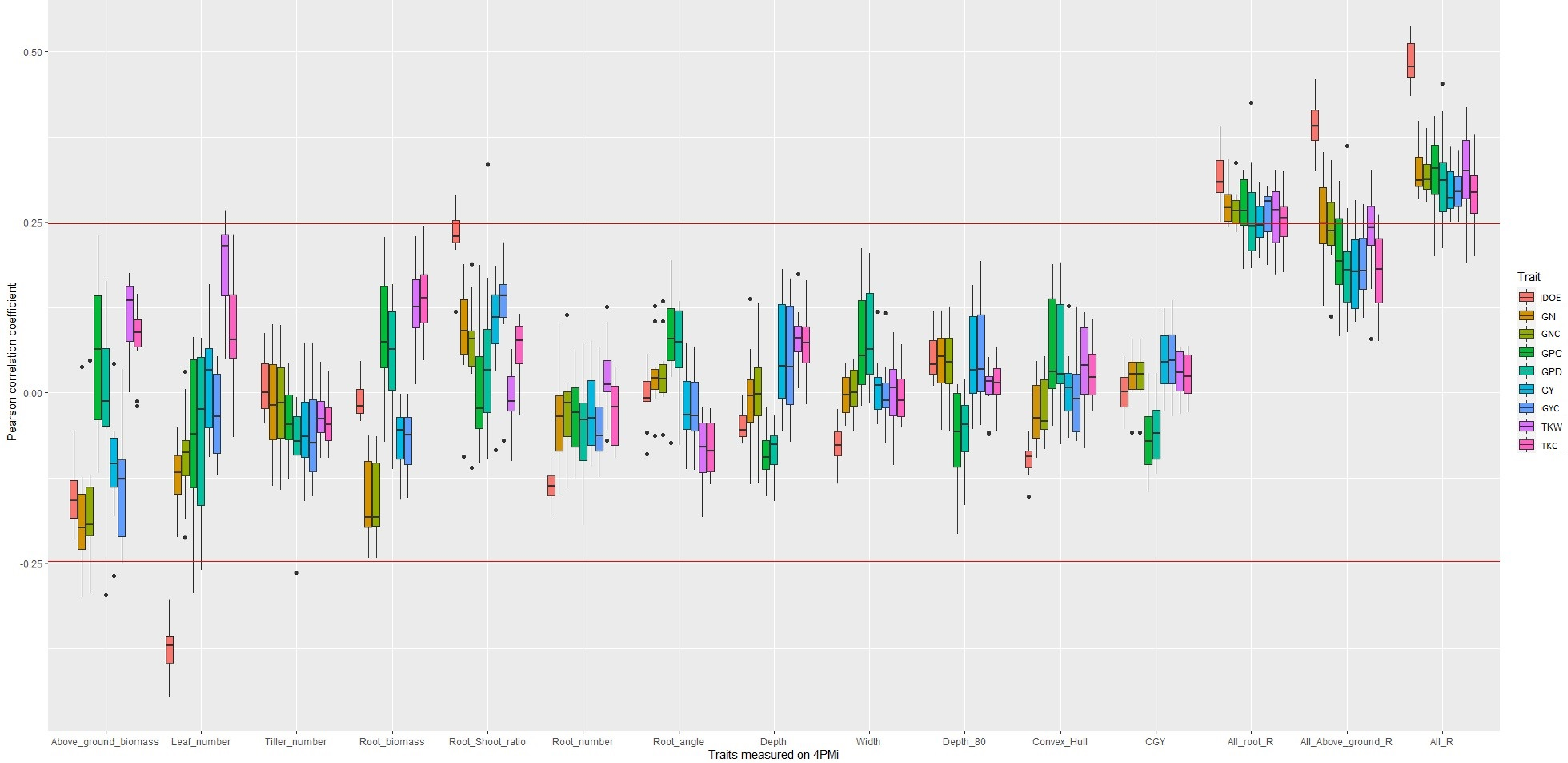
0.50
0.25
0.00
-0.25
Pearson correlation coefficient
Above_ground_biomass
Leaf_number
Tiller_number
Root_biomass
Root_Shoot_ratio
Root_number
Root_angle
Depth
Width
Depth_80
Convex_Hull
CGY
All_root_R
All_Above_ground_R
All_R
Traits measured on 4PMi
Trait
DOE
GN
GNC
GPC
GPD
GY
GYC
TKW
TKC

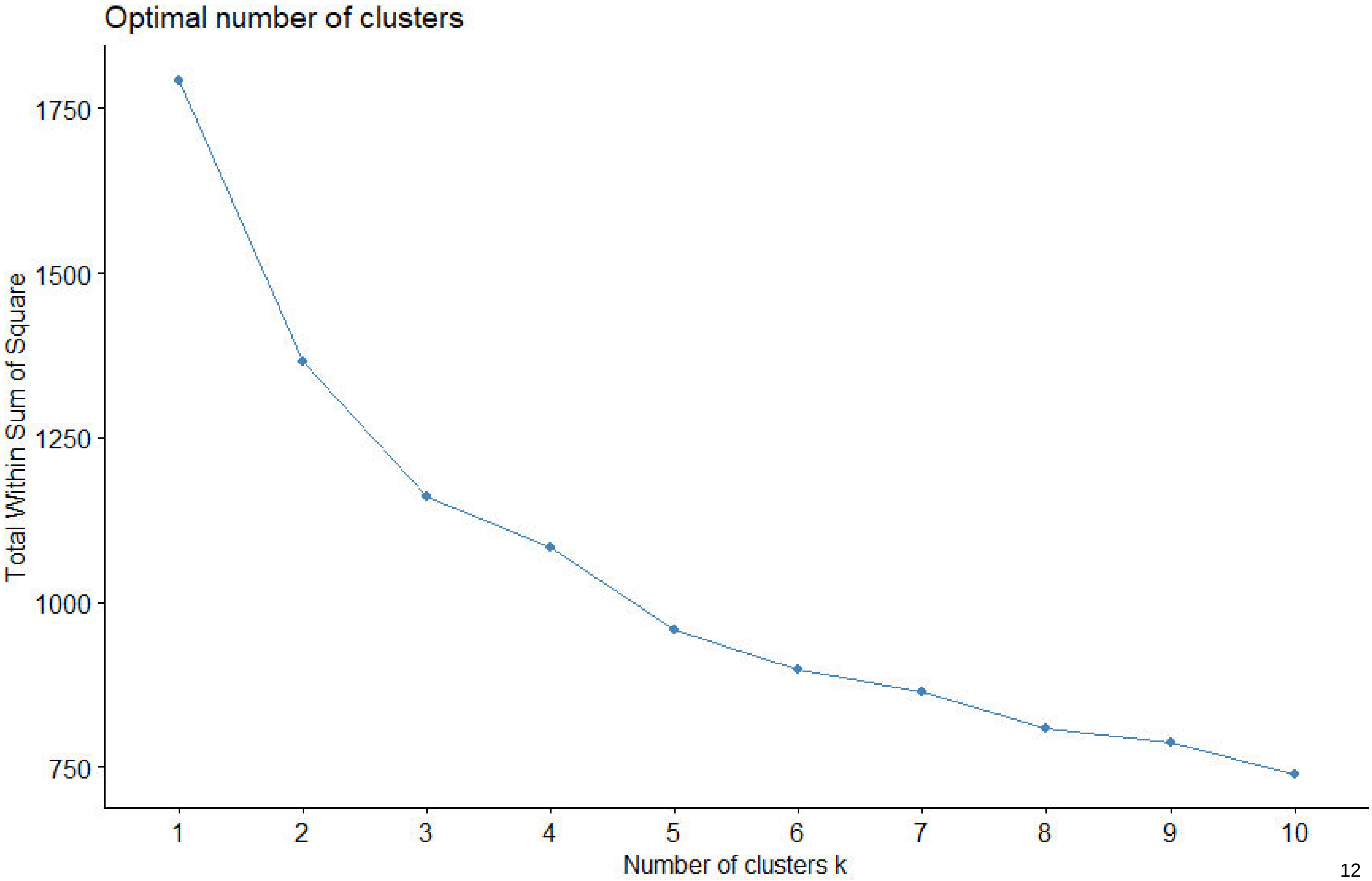
Optimal number of clusters
Total Within Sum of Square
1750
1500
1250
1000
750
1
2
3
4
5
6
7
8
9
10
Number of clusters k

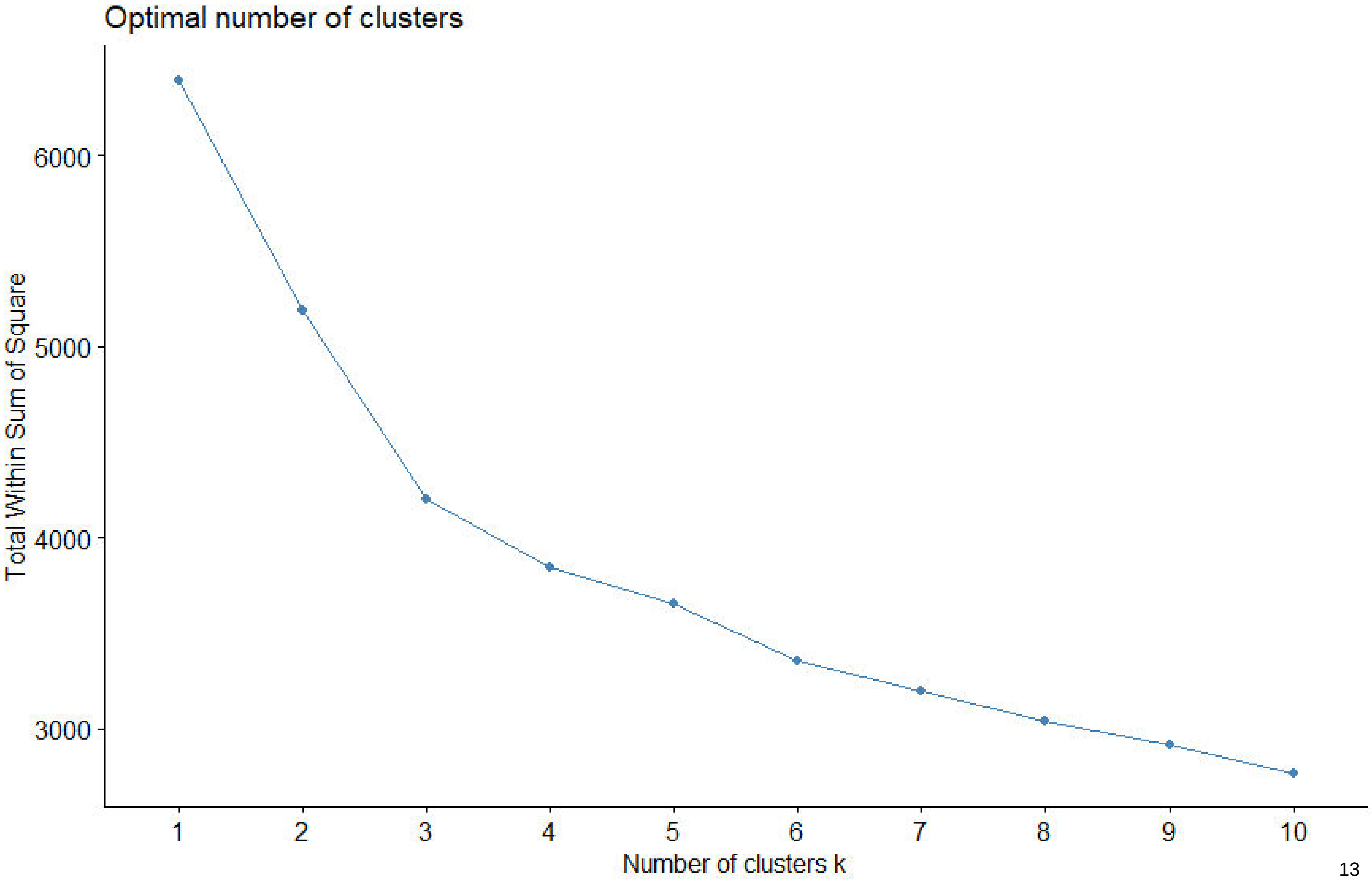
Optimal number of clusters
Total Within Sum of Square
6000
5000
4000
3000
1
2
3
4
5
6
7
8
9
10
Number of clusters k

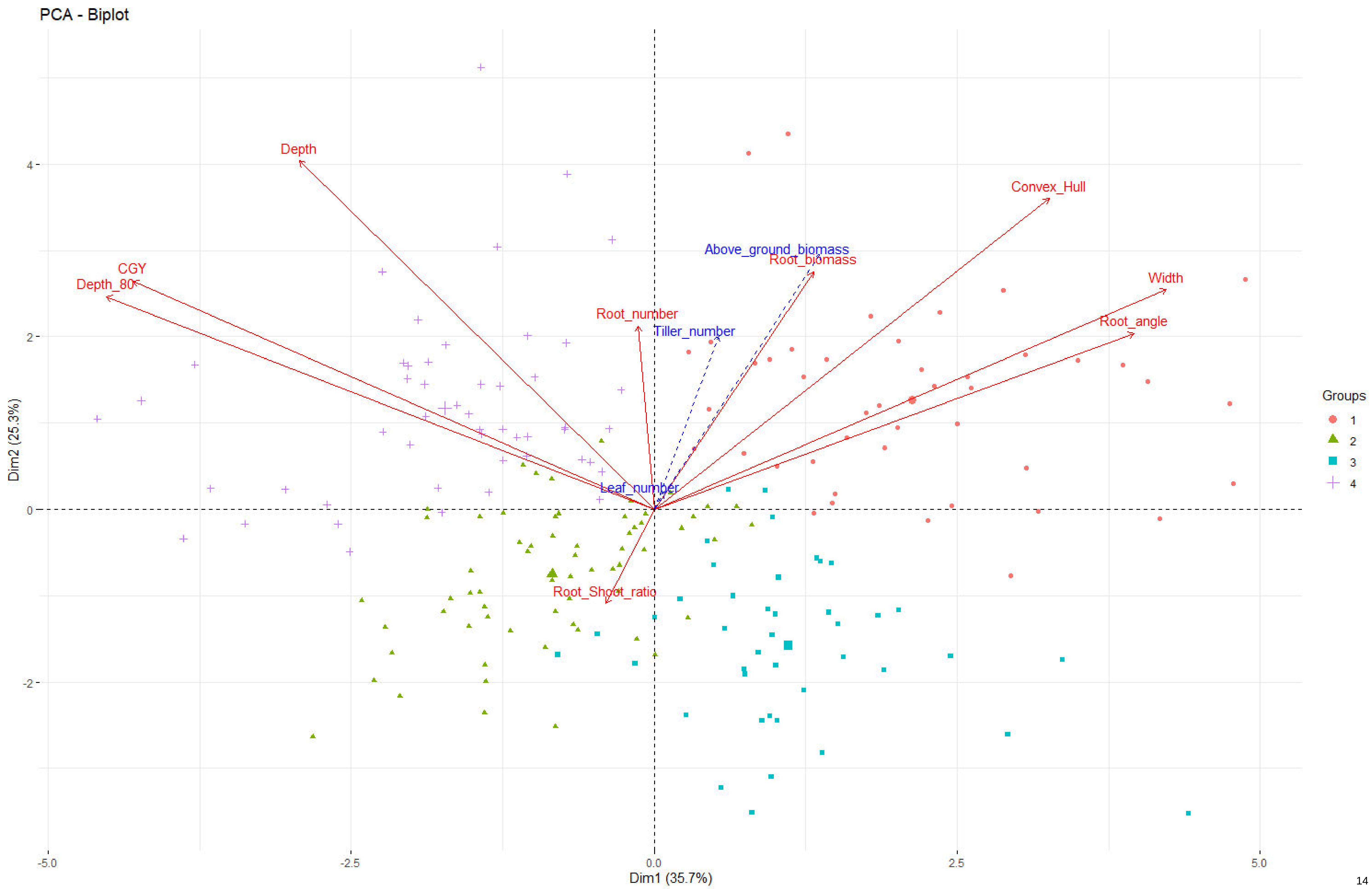
PCA - Biplot
Depth
CGY
Depth_80
Convex_Hull
Above_ground_biomass
Root_biomass
Width
Root_angle
Root_number
Tiller_number
Leaf_number
Root_Shoot_ratio
Groups
1
2
3
4
Dim1 (35.7%)
Dim2 (25.3%)
-5.0
-2.5
0.0
2.5
5.0
4
2
0
-2

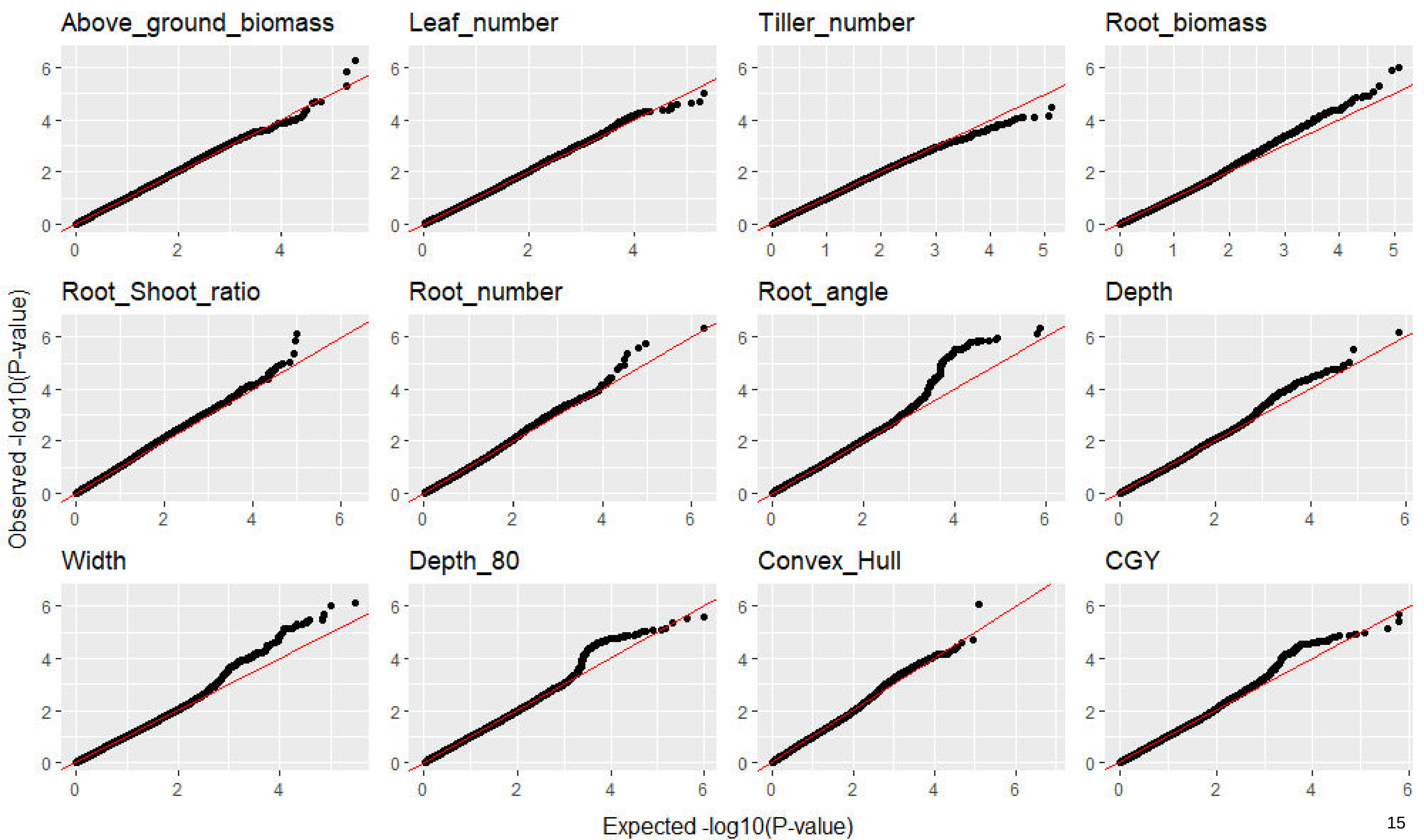
Above_ground_biomass
Leaf_number
Tiller_number
Root_biomass
Root_Shoot_ratio
Root_number
Root_angle
Depth
Width
Depth_80
Convex_Hull
CGY
Observed -log10(P-value)
Expected -log10(P-value)

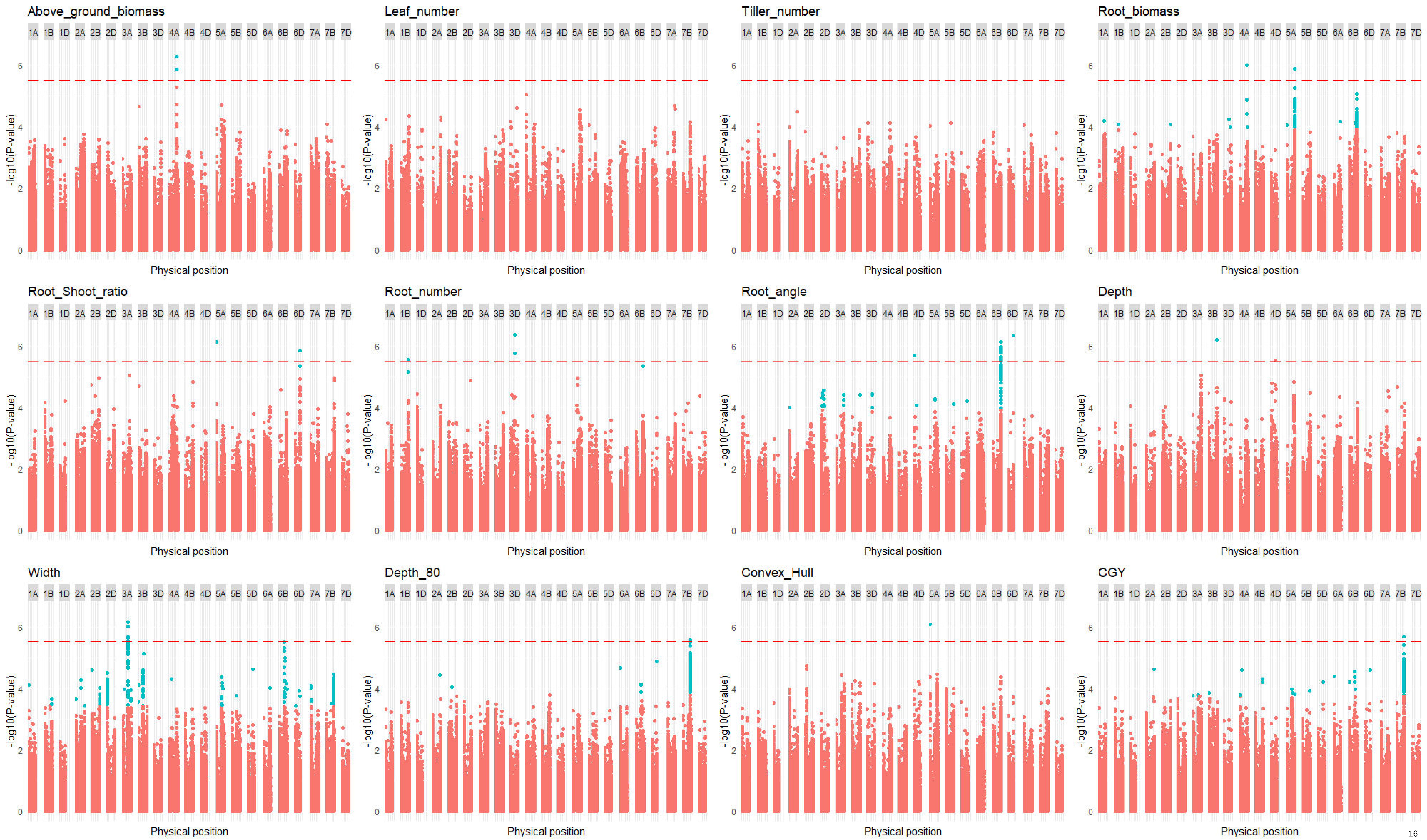
Above_ground_biomass
Leaf_number
Tiller_number
Root_biomass
Root_Shoot_ratio
Root_number
Root_angle
Depth
Width
Depth_80
Convex_Hull
CGY
1A 1B 1D 2A 2B 2D 3A 3B 3D 4A 4B 4D 5A 5B 5D 6A 6B 6D 7A 7B 7D
-log10(P-value)
Physical position

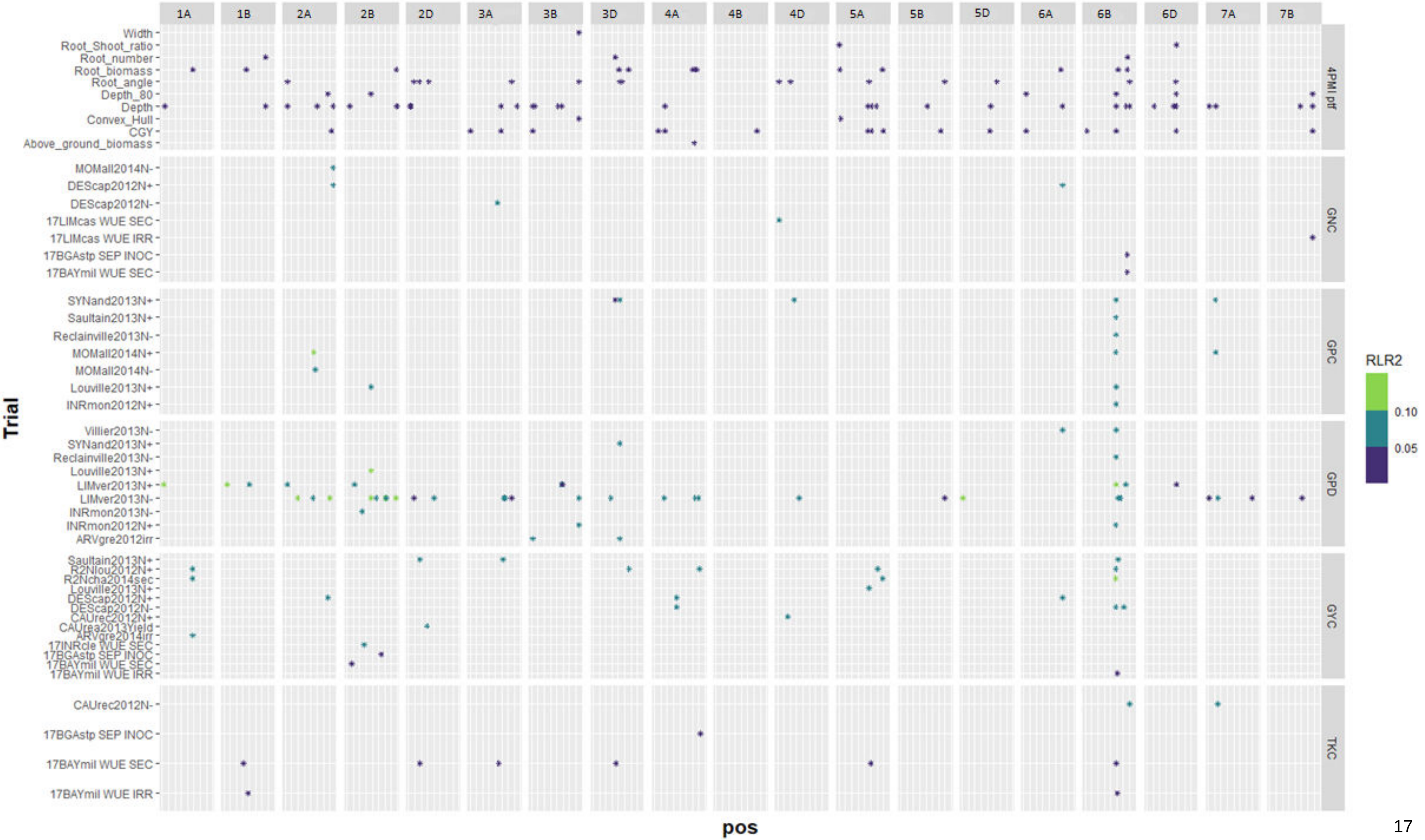

1A
1B
2A
2B
2D
3A
3B
3D
4A
4B
4D
5A
5B
5D
6A
6B
6D
7A
7B
Width
Root_Shoot_ratio
Root_number
Root_biomass
Root_angle
Depth_80
Depth
Convex_Hull
CGY
Above_ground_biomass
4PMI ptf
MOMall2014N-
DEScap2012N+
DEScap2012N-
17LIMcas WUE SEC
17LIMcas WUE IRR
17BGAstp SEP INOC
17BAYmil WUE SEC
GNC
SYNand2013N+
Saultain2013N+
Reclainville2013N-
MOMall2014N+
MOMall2014N-
Louville2013N+
INRmon2012N+
GPC
Villier2013N-
SYNand2013N+
Reclainville2013N-
Louville2013N+
LIMver2013N+
LIMver2013N-
INRmon2013N-
INRmon2012N+
ARVgre2012irr
GPD
Saultain2013N+
R2Nlou2012N+
R2Ncha2014sec
Louville2013N+
DEScap2012N+
DEScap2012N-
CAUrec2012N+
CAUrea2013Yield
ARVgre2014irr
17INRcle WUE SEC
17BGAstp SEP INOC
17BAYmil WUE SEC
17BAYmil WUE IRR
GYC
CAUrec2012N-
17BGAstp SEP INOC
17BAYmil WUE SEC
17BAYmil WUE IRR
TKC
Trial
pos
RLR2
0.10
0.05

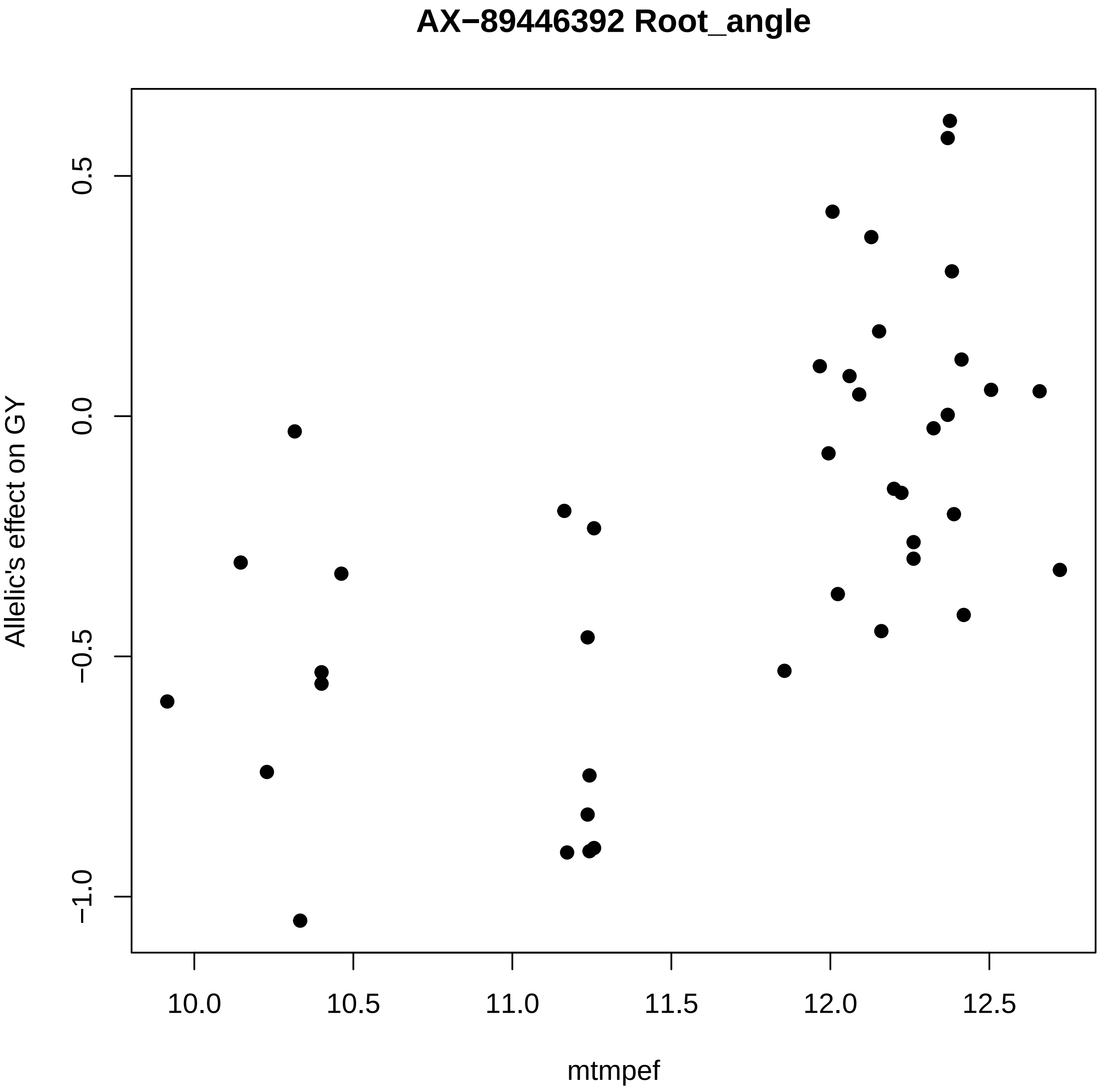
AX−89446392 Root_angle
Allelic's effect on GY
mtmpef
0.5
0.0
−0.5
−1.0
10.0
10.5
11.0
11.5
12.0
12.5

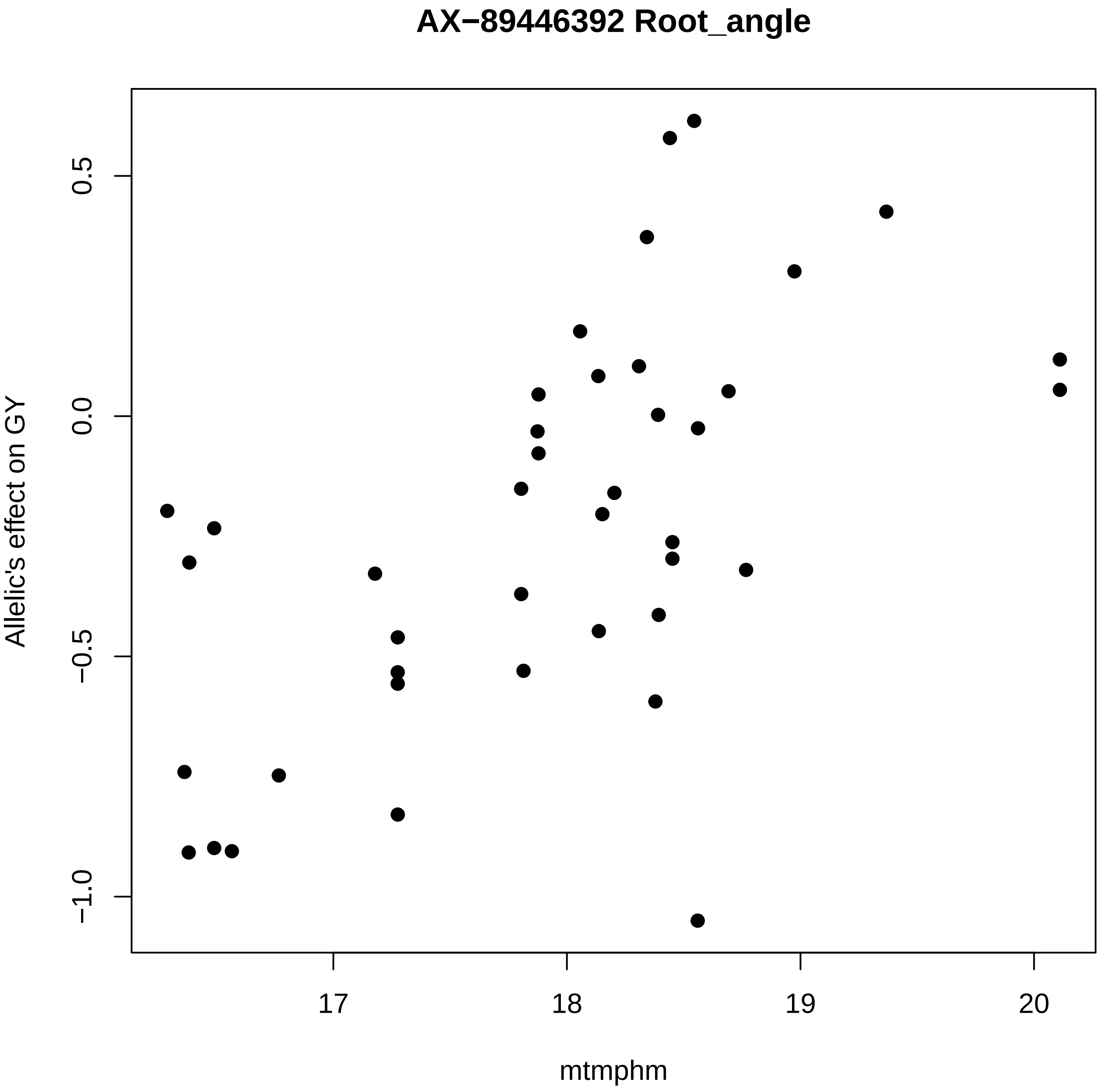

AX−89446392 Root_angle
Allelic's effect on GY
0.5
0.0
−0.5
−1.0
17
18
19
20
mtmphm

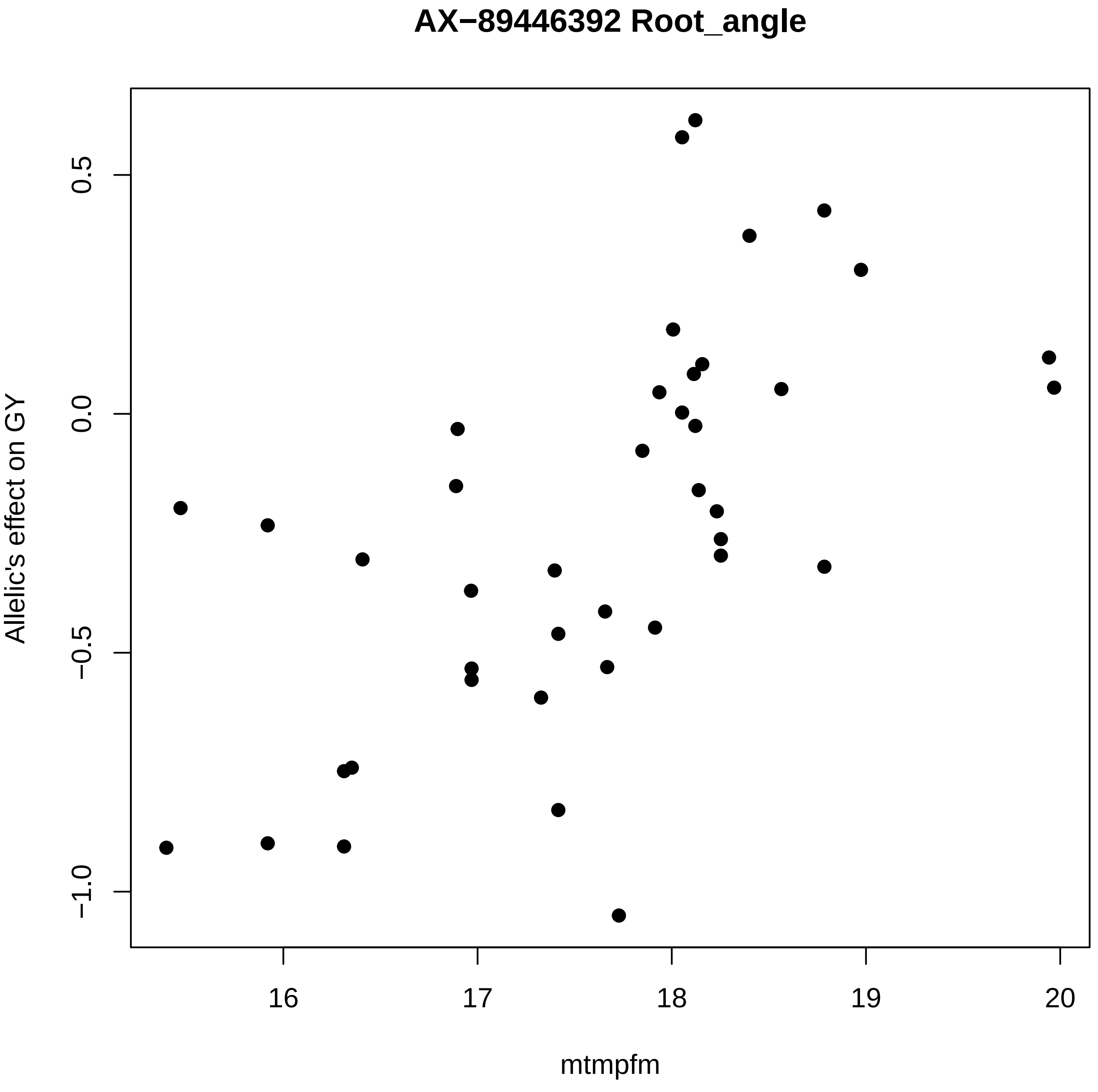
AX−89446392 Root_angle
Allelic's effect on GY
0.5
0.0
−0.5
−1.0
16
17
18
19
20
mtmpfm

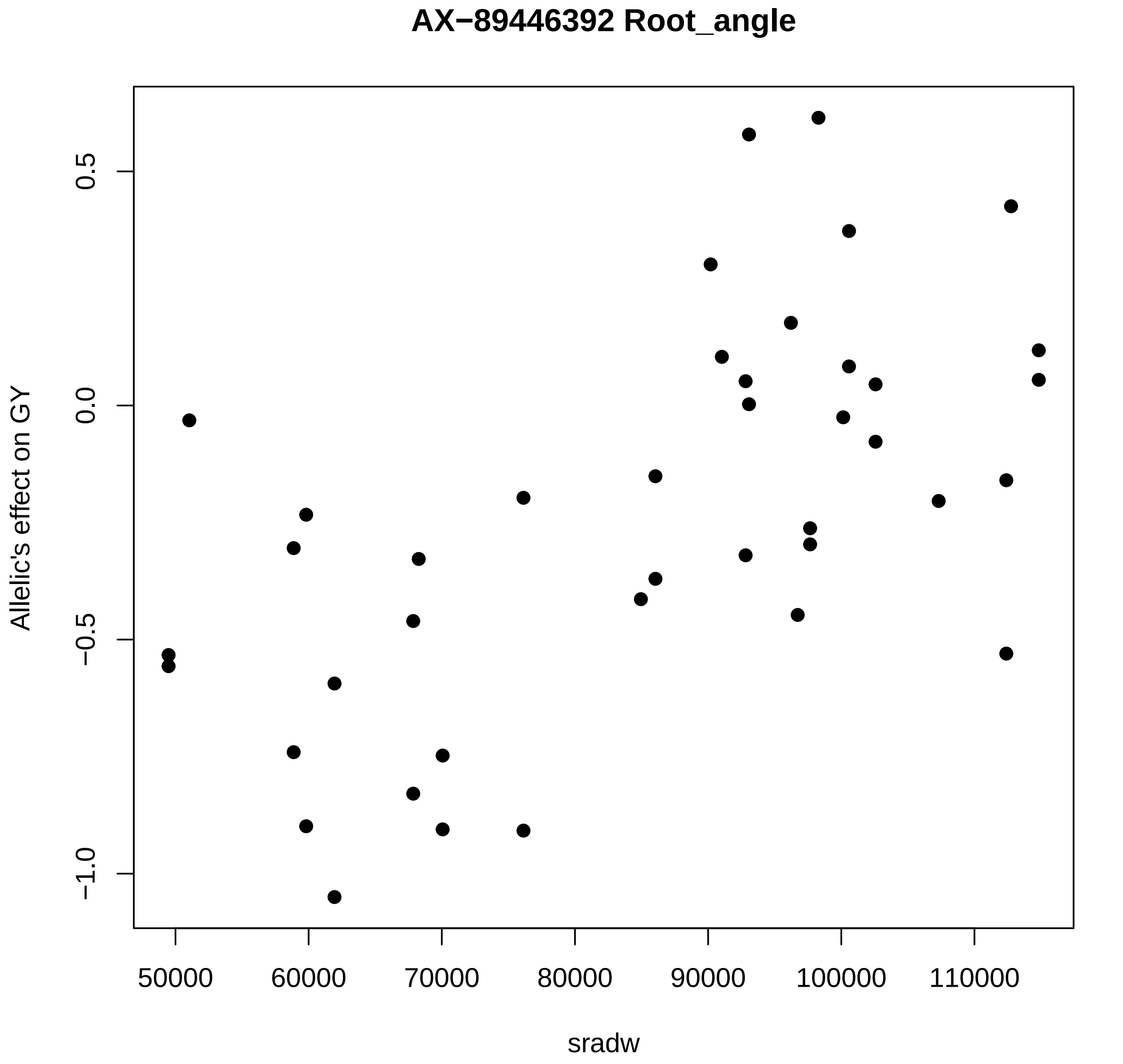
AX−89446392 Root_angle
0.5
0.0
−0.5
−1.0
Allelic's effect on GY
50000
60000
70000
80000
90000
100000
110000
sradw

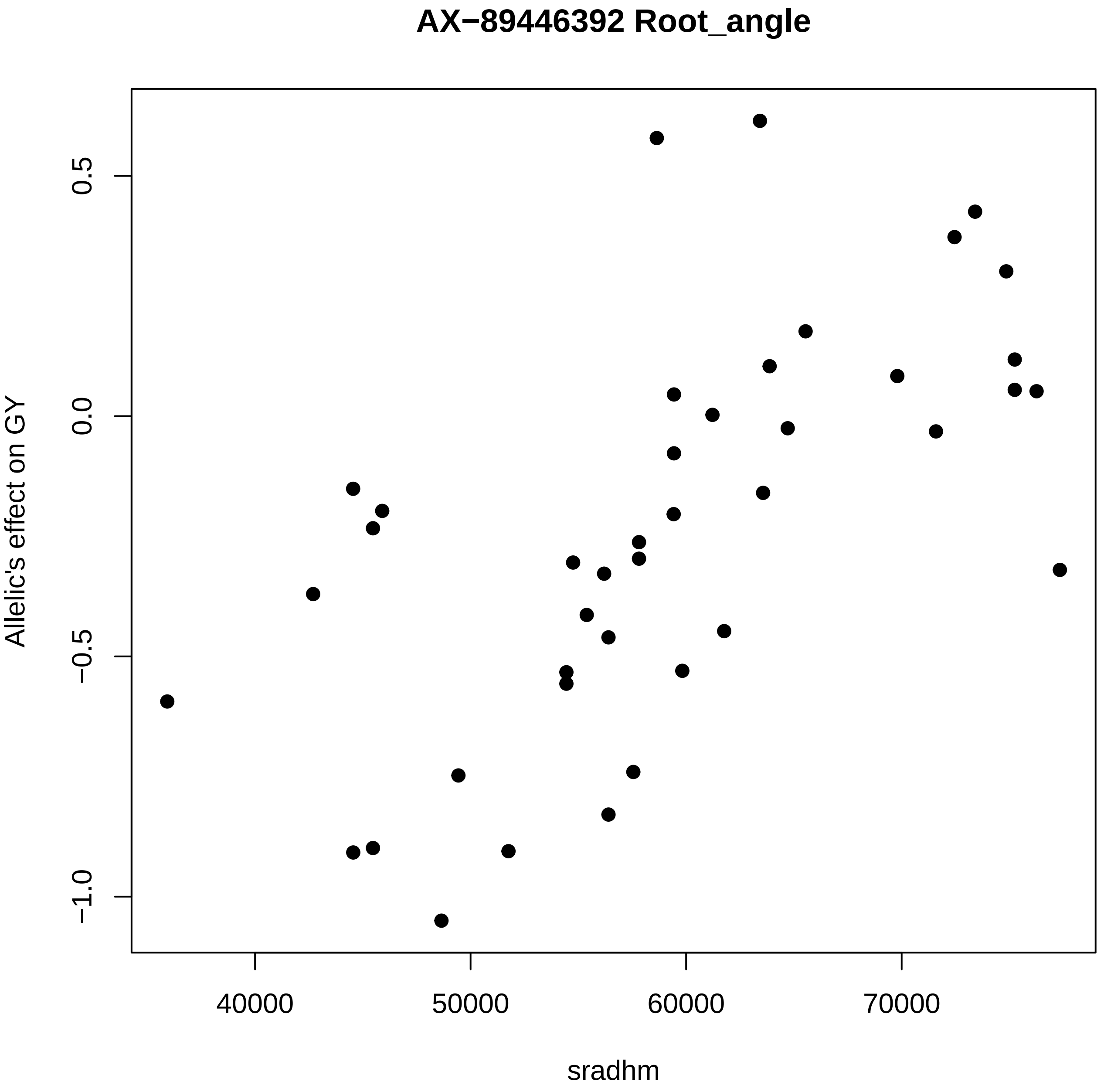
AX−89446392 Root_angle
0.5
0.0
−0.5
−1.0
Allelic's effect on GY
40000
50000
60000
70000
sradhm

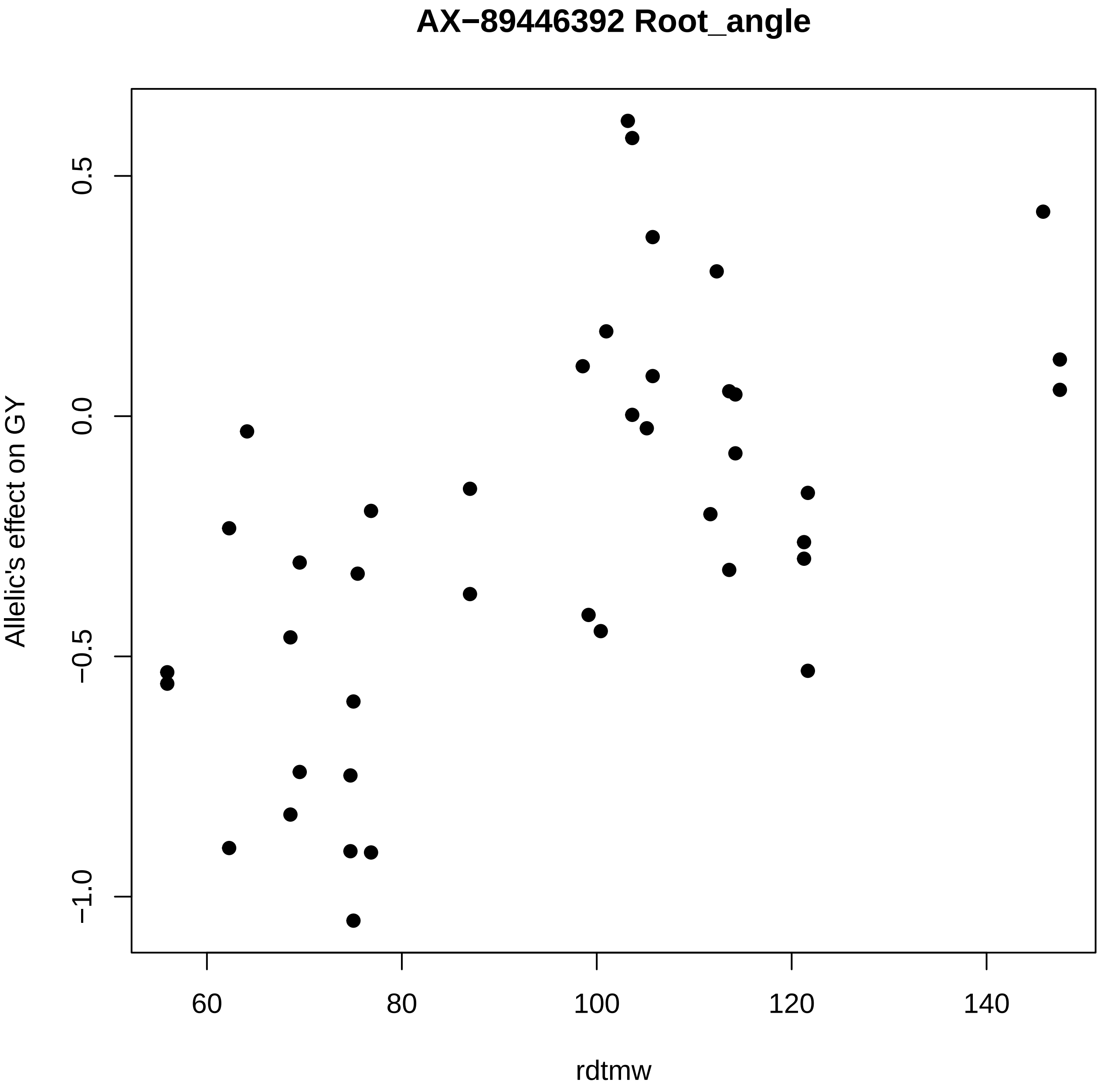
AX−89446392 Root_angle
Allelic's effect on GY
rdtmw
0.5
0.0
−0.5
−1.0
60
80
100
120
140

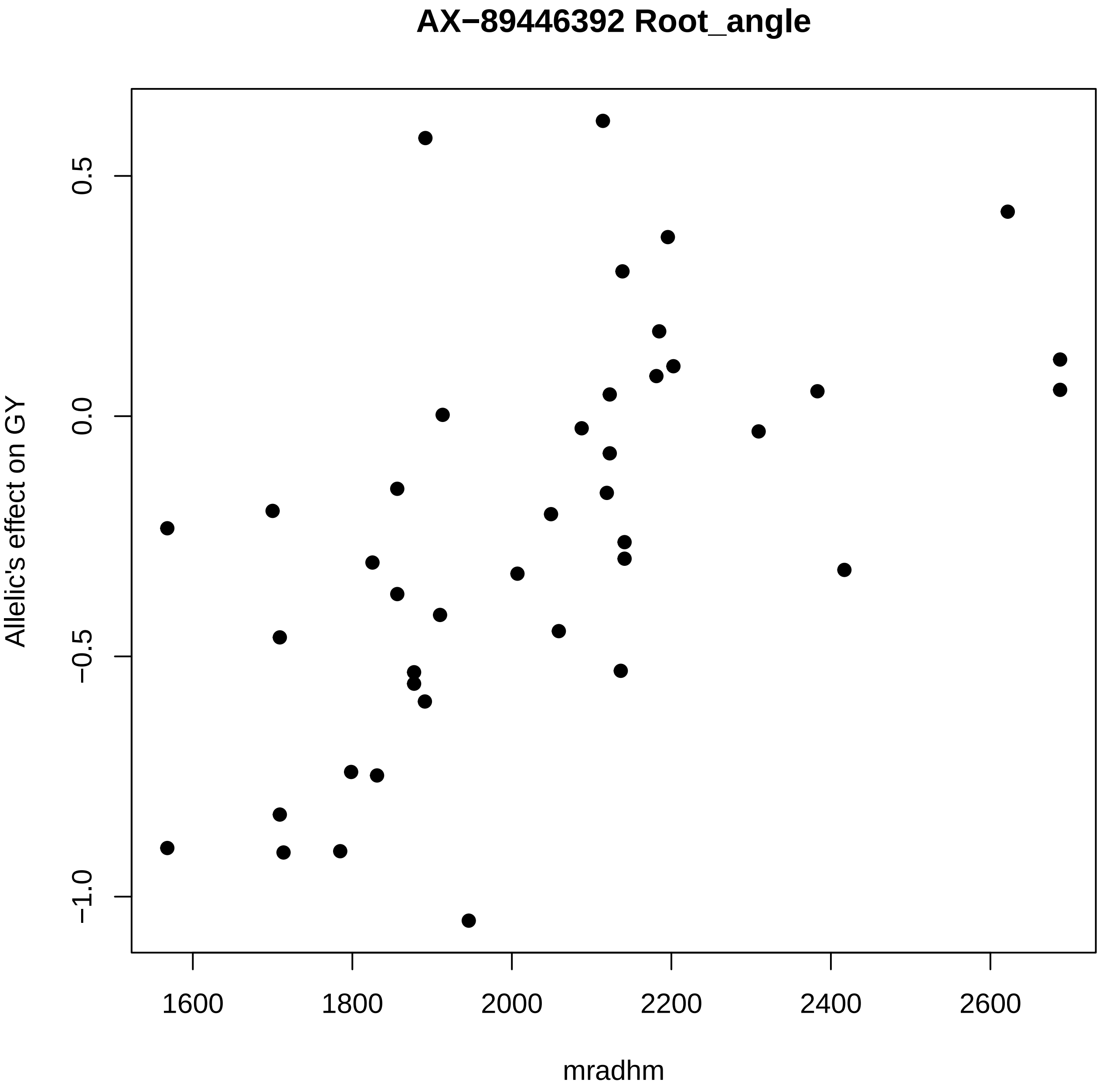
AX−89446392 Root_angle
Allelic's effect on GY
mradhm
0.5
0.0
−0.5
−1.0
1600
1800
2000
2200
2400
2600

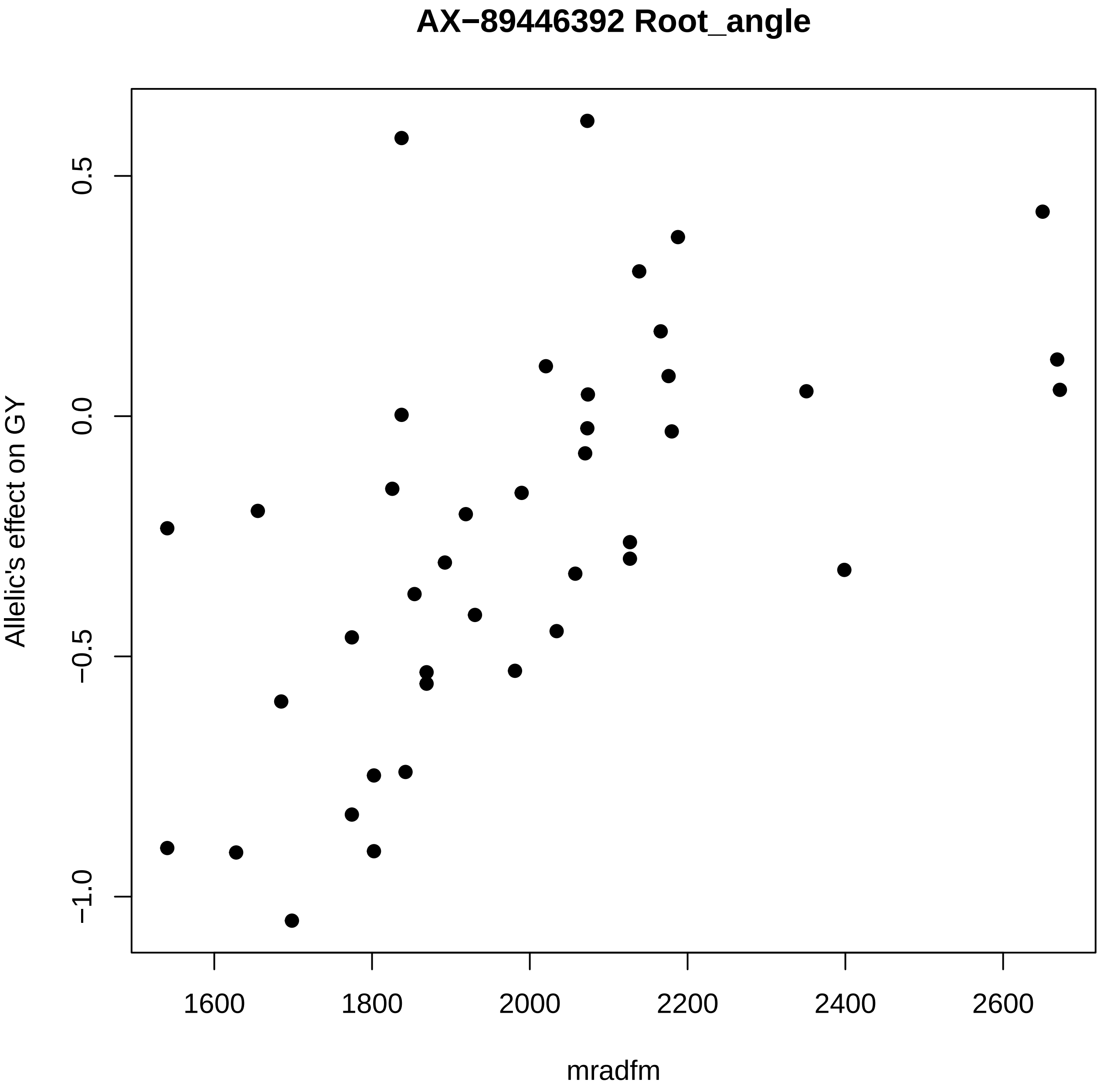

AX−89446392 Root_angle
0.5
0.0
−0.5
−1.0
Allelic's effect on GY
1600
1800
2000
2200
2400
2600
mradfm

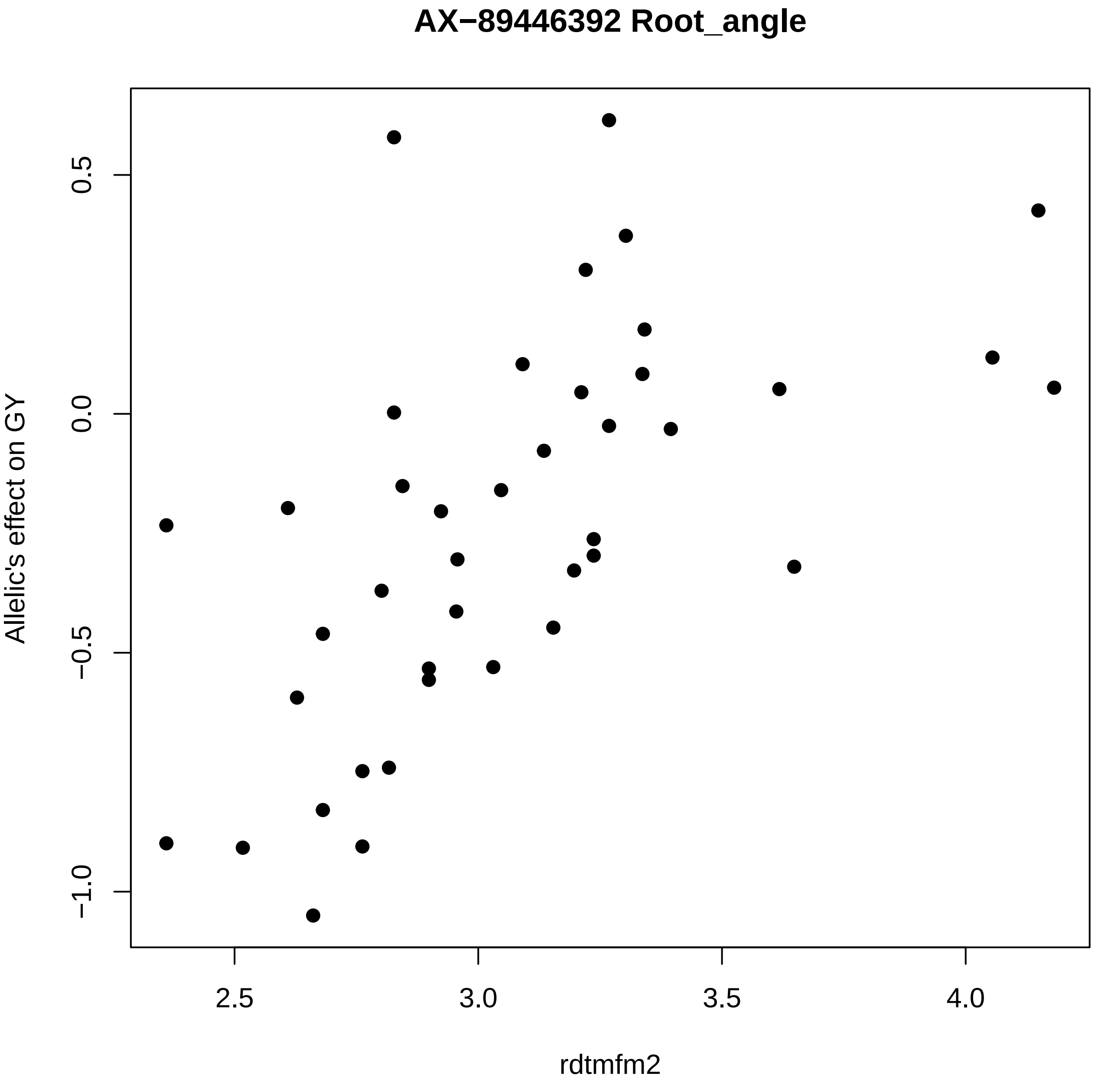

AX−89446392 Root_angle
0.5
0.0
−0.5
−1.0
Allelic's effect on GY
2.5
3.0
3.5
4.0
rdtmfm2

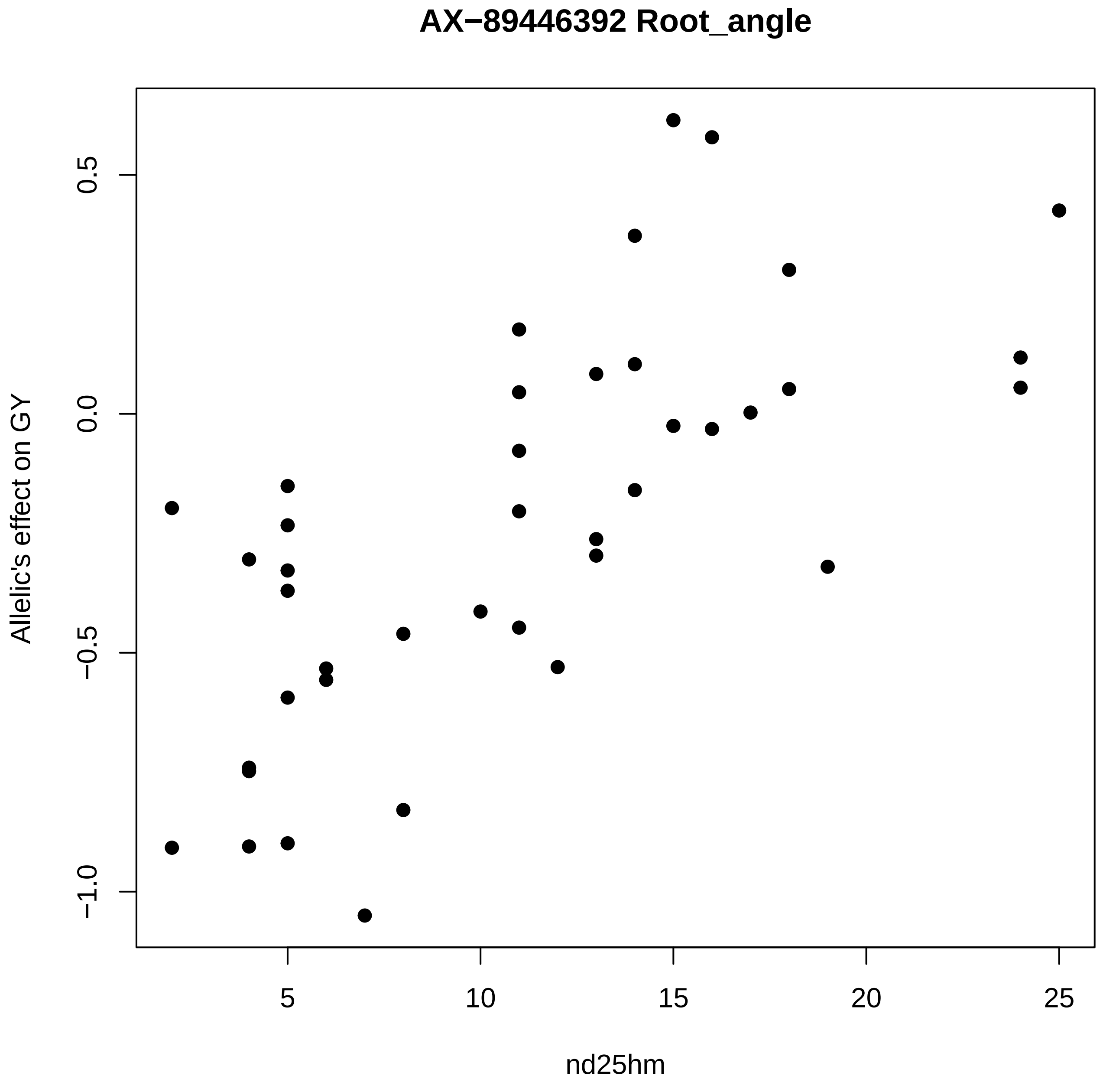
AX−89446392 Root_angle
Allelic's effect on GY
nd25hm
0.5
0.0
−0.5
−1.0
5
10
15
20
25

AX−89446392 Root_angle

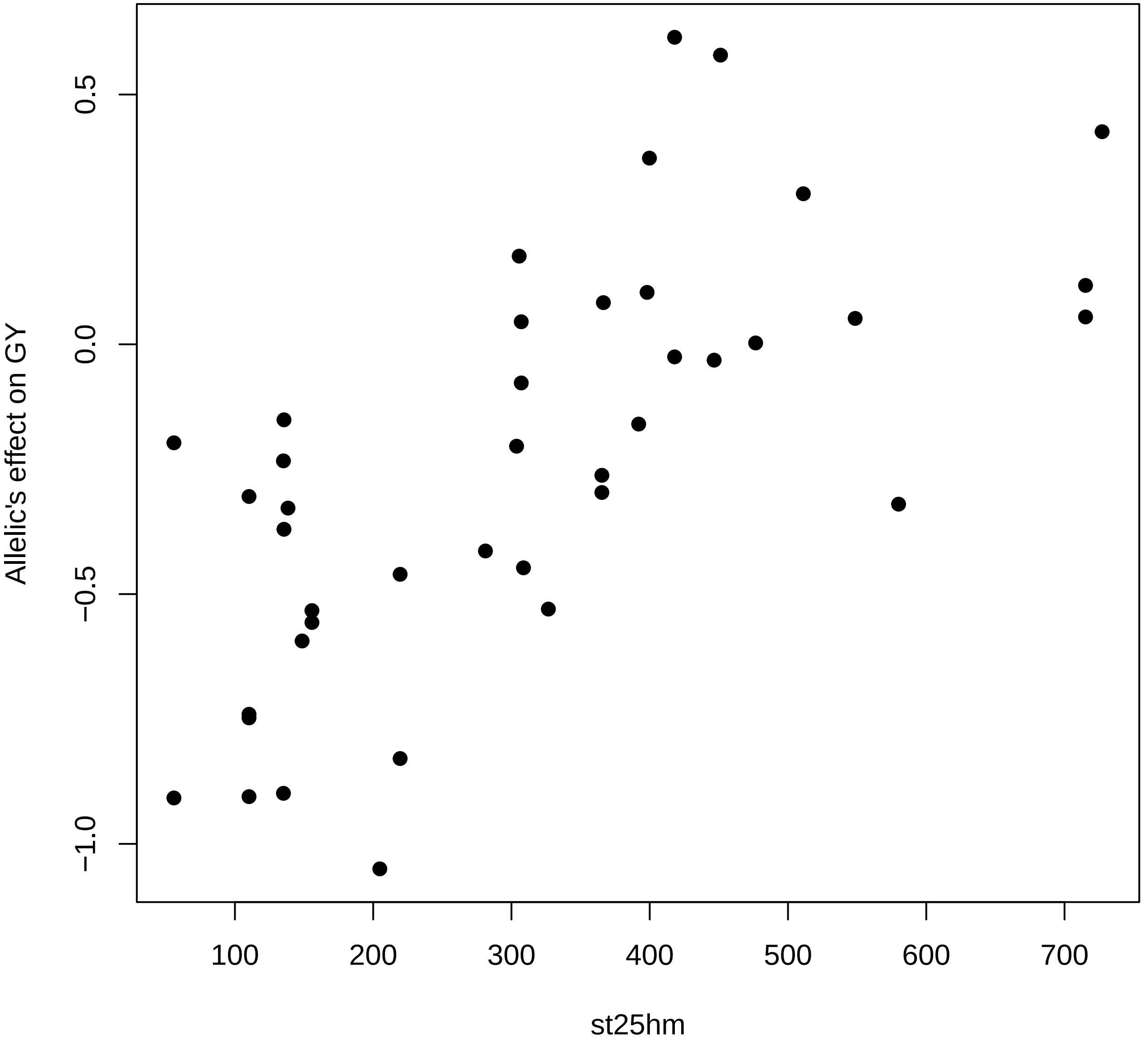

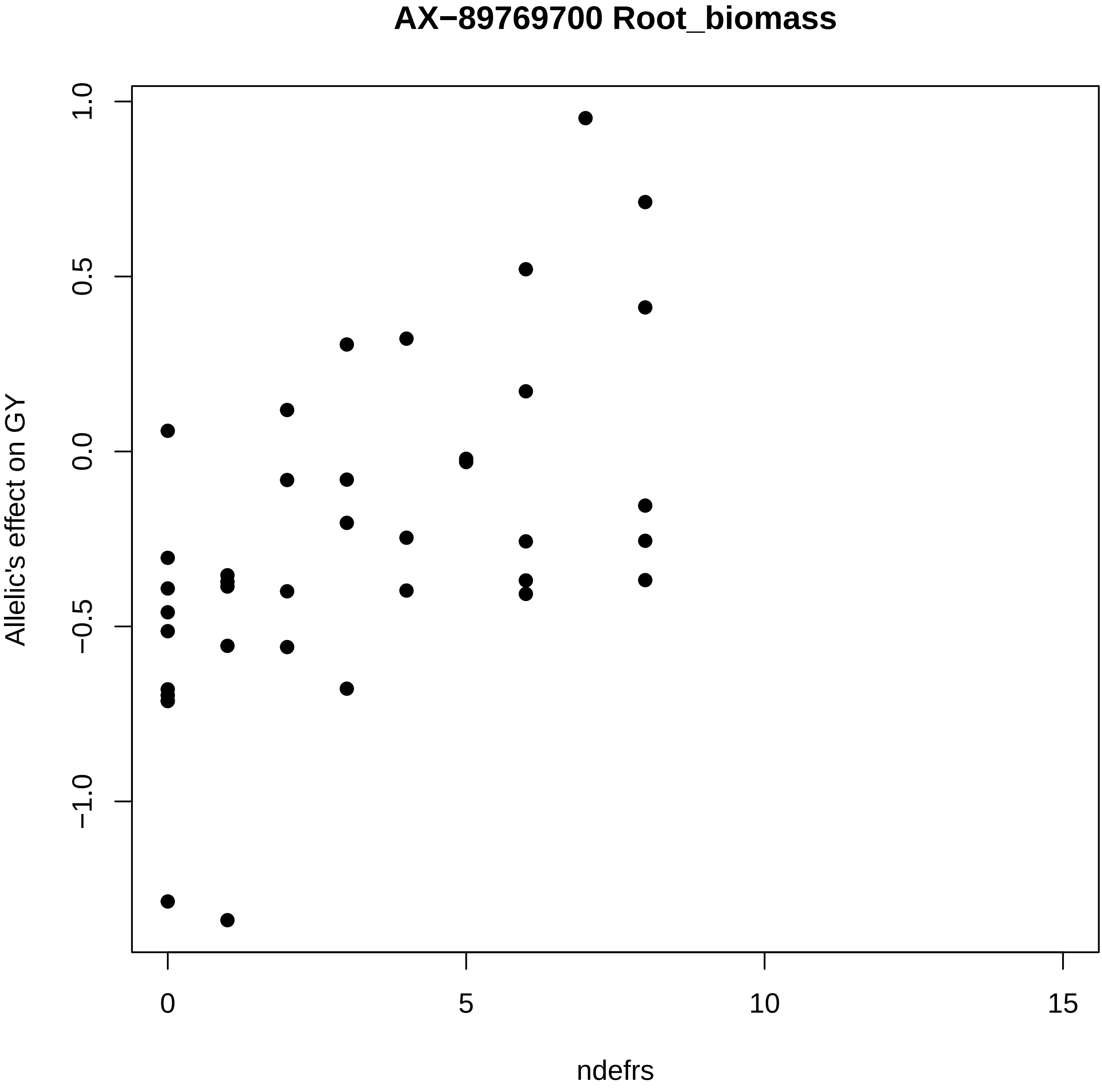

AX−89769700 Root_biomass
Allelic's effect on GY
1.0
0.5
0.0
−0.5
−1.0
0
5
10
15
ndefrs

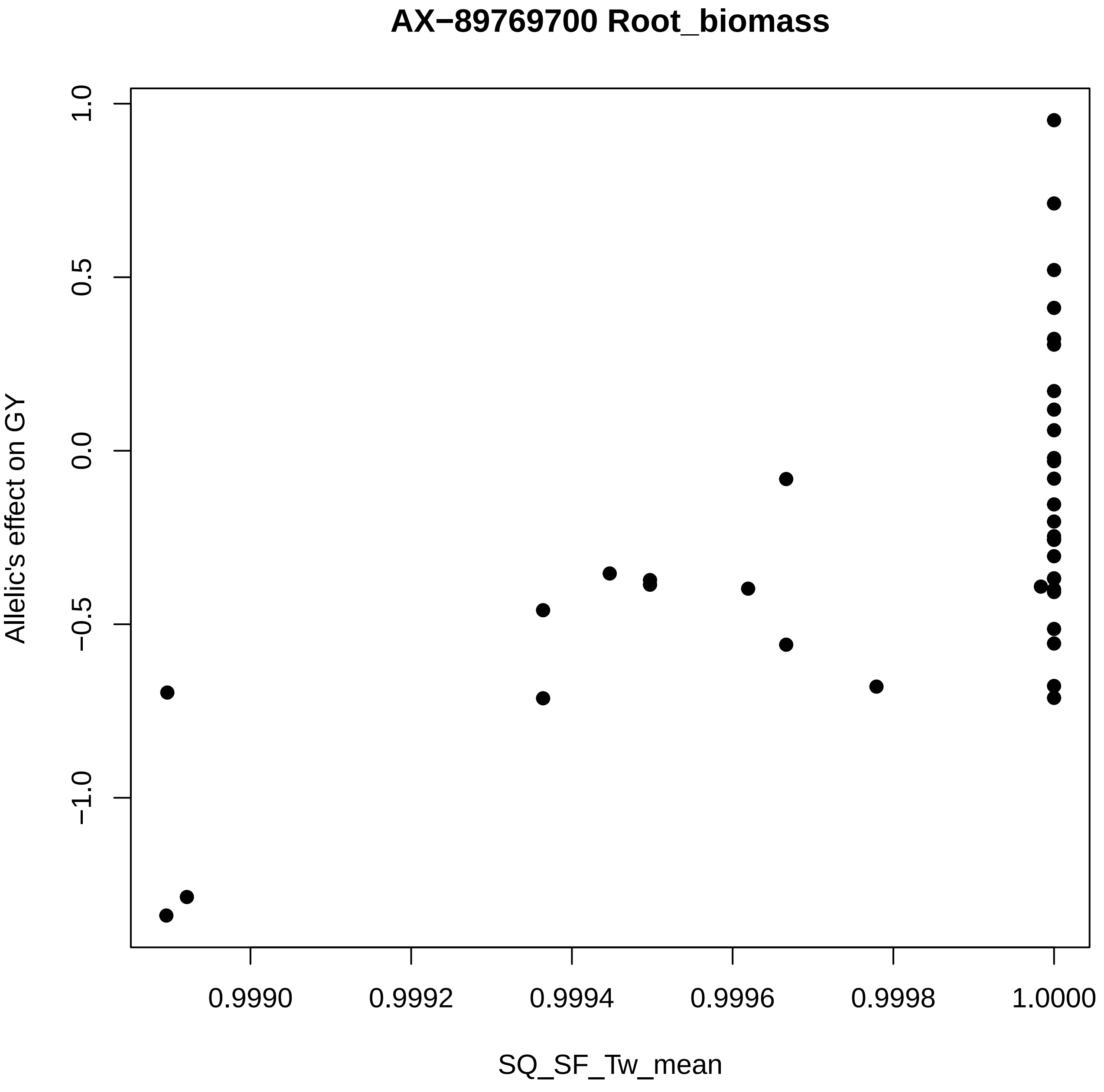

AX−89769700 Root_biomass
Allelic's effect on GY
1.0
0.5
0.0
−0.5
−1.0
0.9990
0.9992
0.9994
0.9996
0.9998
1.0000
SQ_SF_Tw_mean

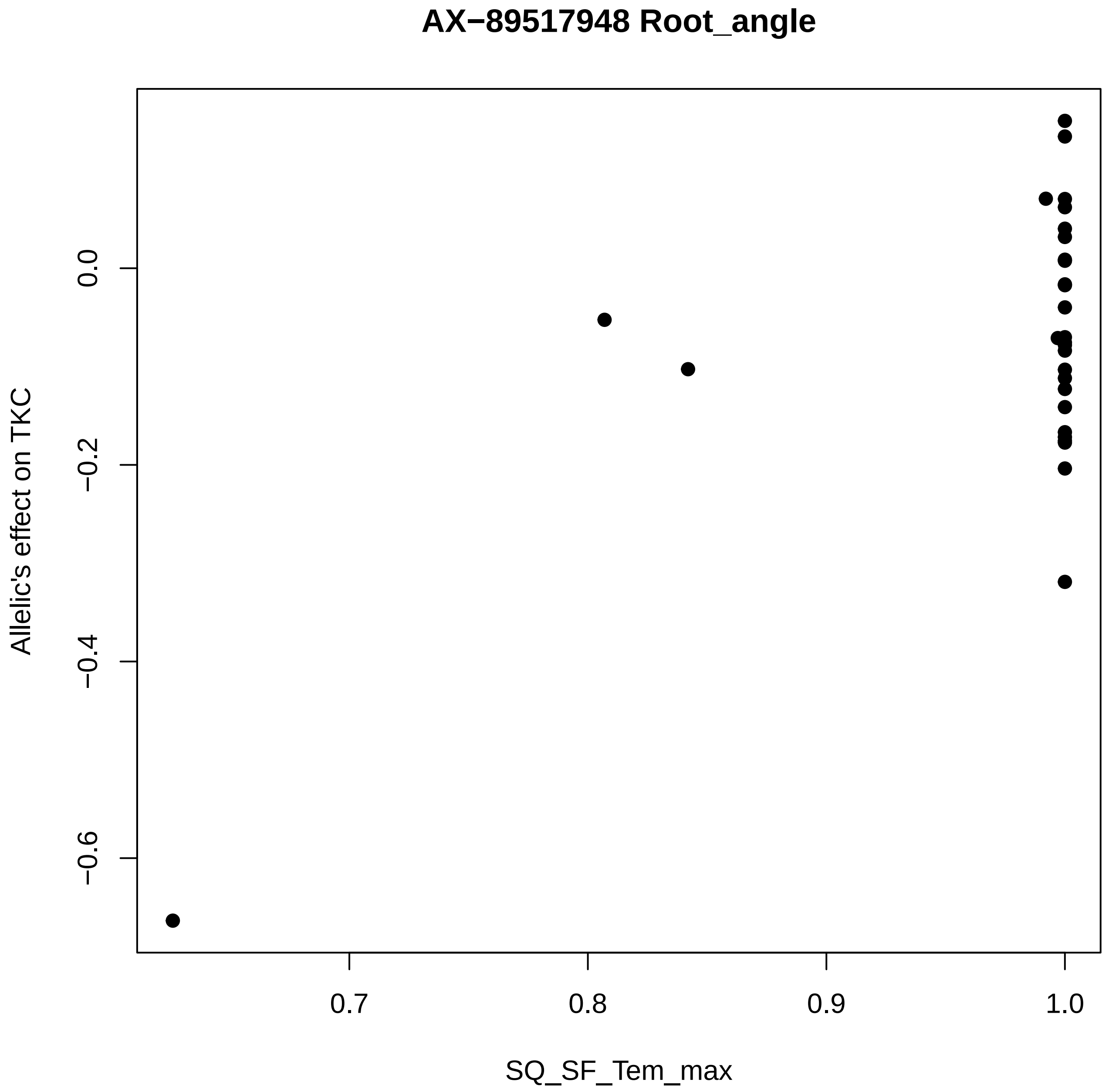

AX−89517948 Root_angle
Allelic's effect on TKC
0.0
−0.2
−0.4
−0.6
0.7
0.8
0.9
1.0
SQ_SF_Tem_max

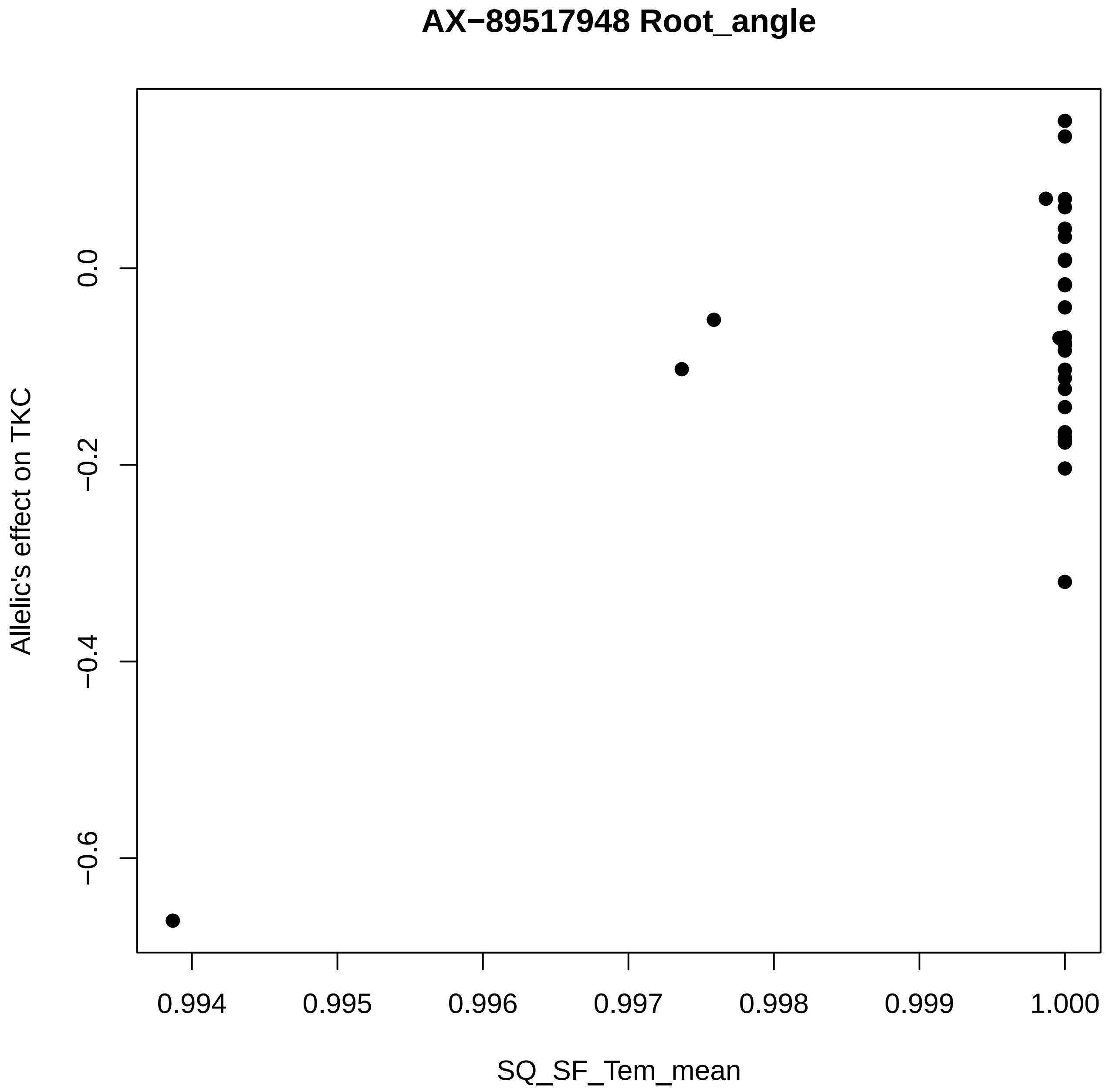
AX−89517948 Root_angle
Allelic's effect on TKC
0.0
−0.2
−0.4
−0.6
0.994
0.995
0.996
0.997
0.998
0.999
1.000
SQ_SF_Tem_mean

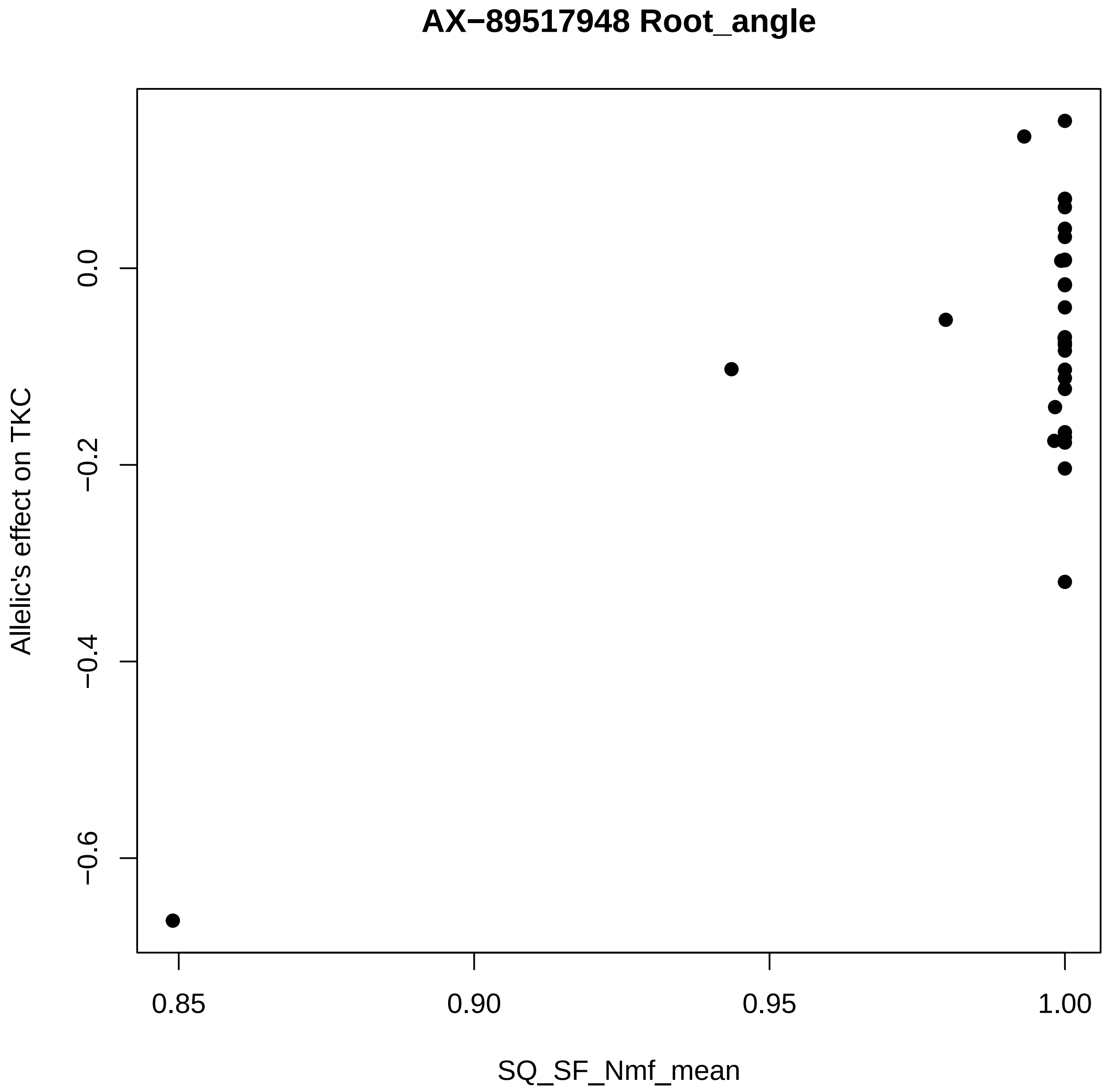
AX−89517948 Root_angle
Allelic's effect on TKC
0.0
−0.2
−0.4
−0.6
0.85
0.90
0.95
1.00
SQ_SF_Nmf_mean

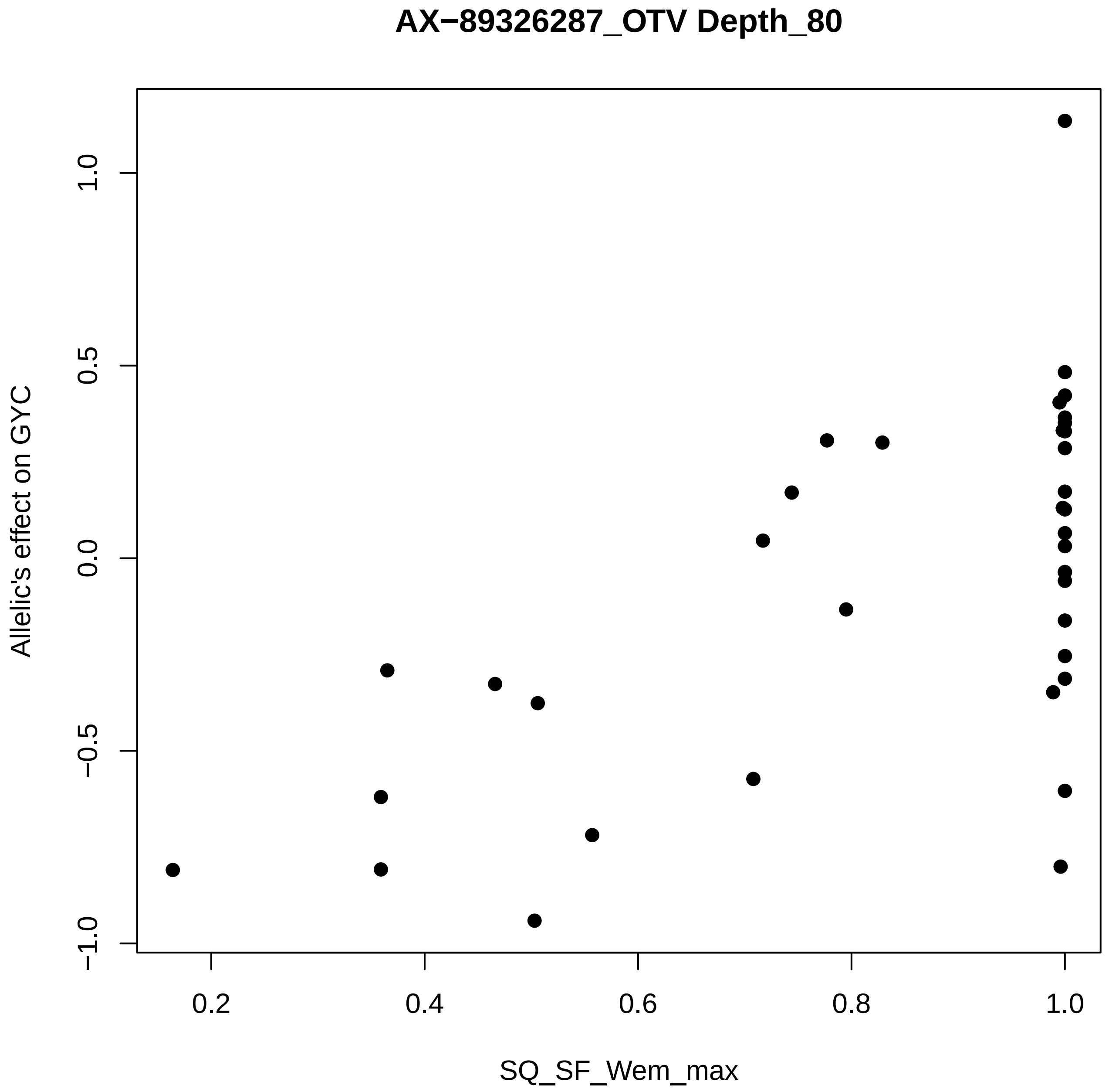

AX−89326287_OTV Depth_80
Allelic's effect on GYC
SQ_SF_Wem_max
1.0
0.5
0.0
−0.5
−1.0
0.2
0.4
0.6
0.8
1.0

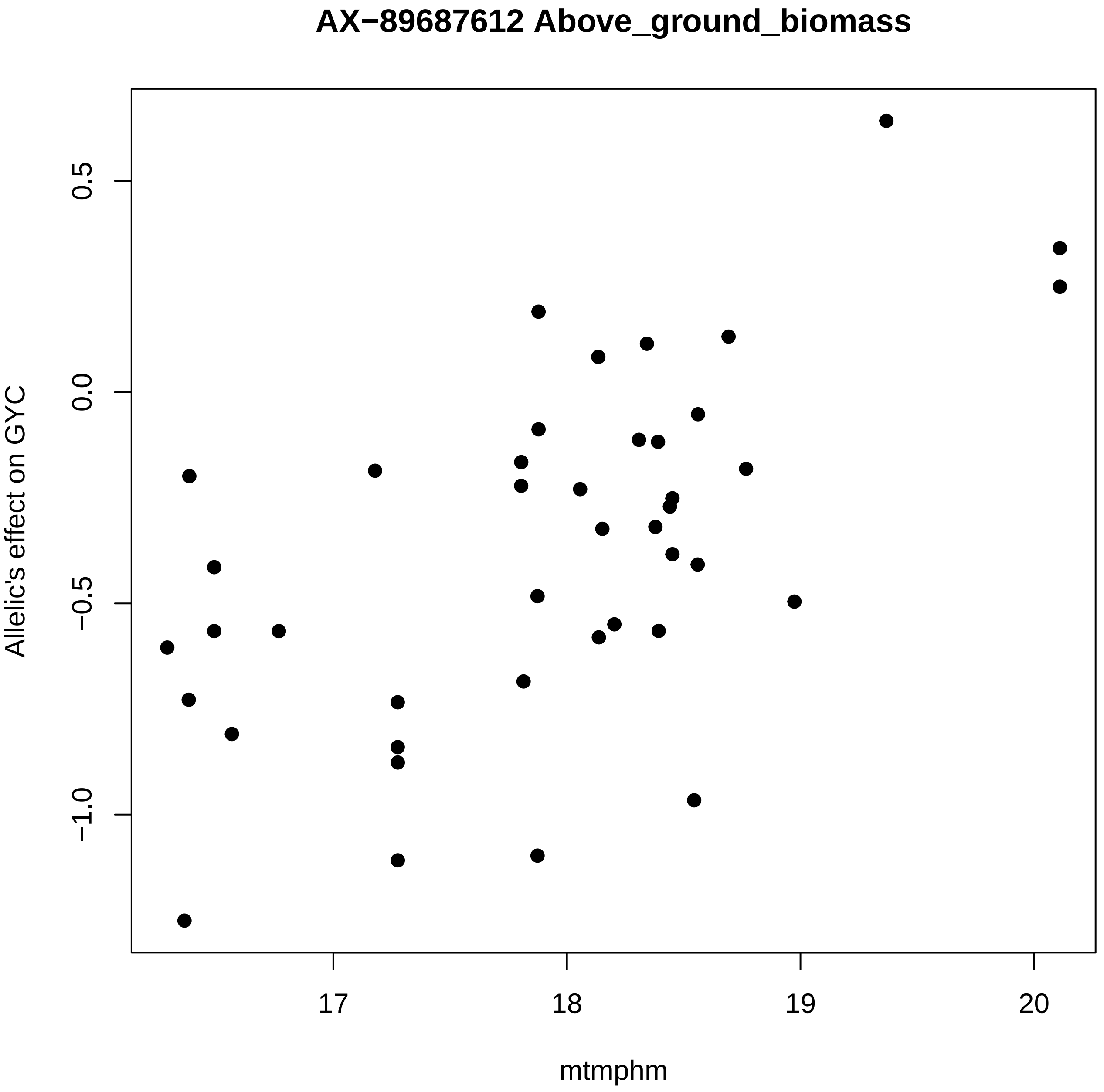

AX−89687612 Above_ground_biomass
Allelic's effect on GYC
0.5
0.0
−0.5
−1.0
17
18
19
20
mtmphm

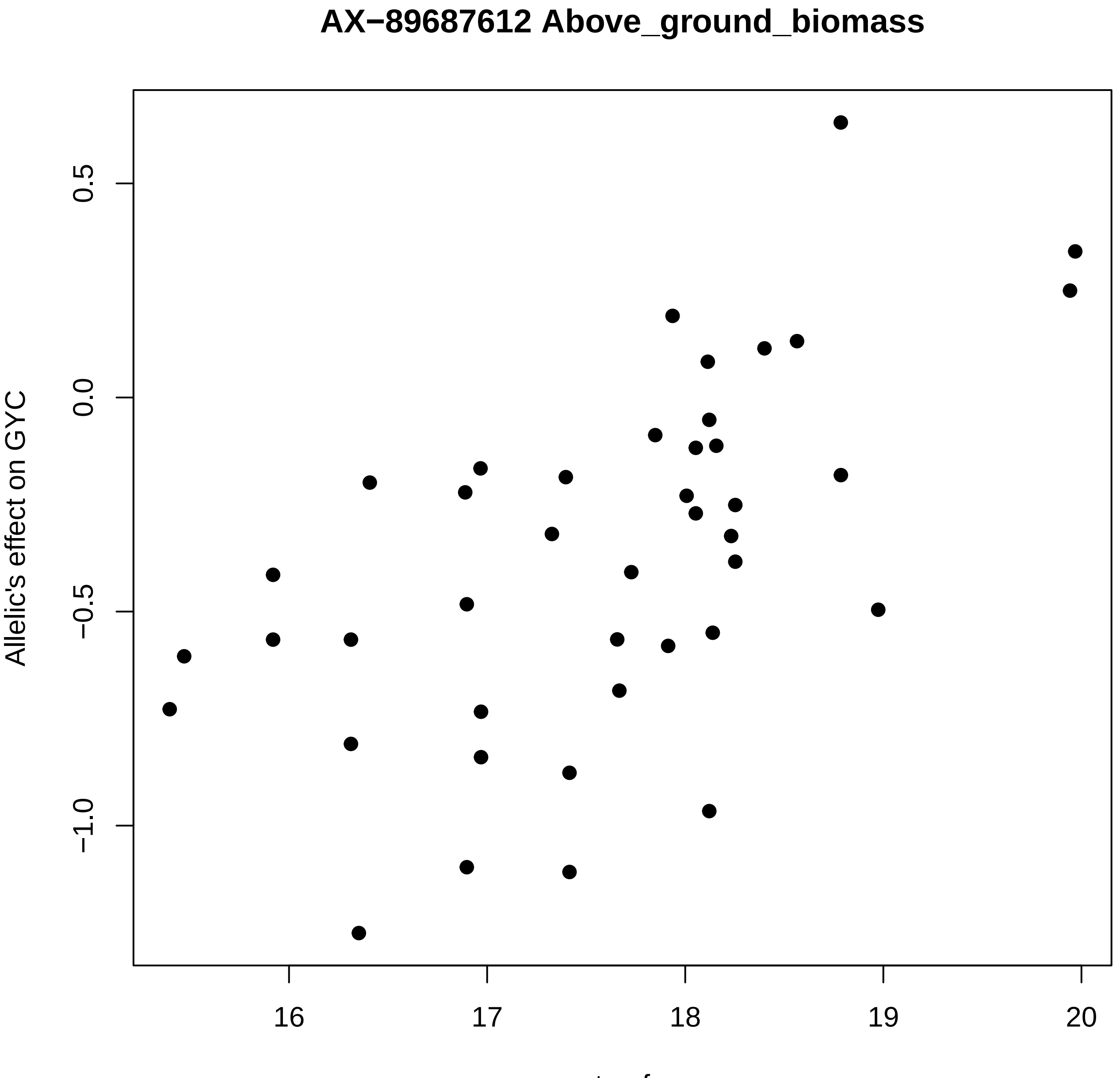

AX−89687612 Above_ground_biomass
Allelic's effect on GYC
0.5
0.0
−0.5
−1.0
16
17
18
19
20
mtmpfm

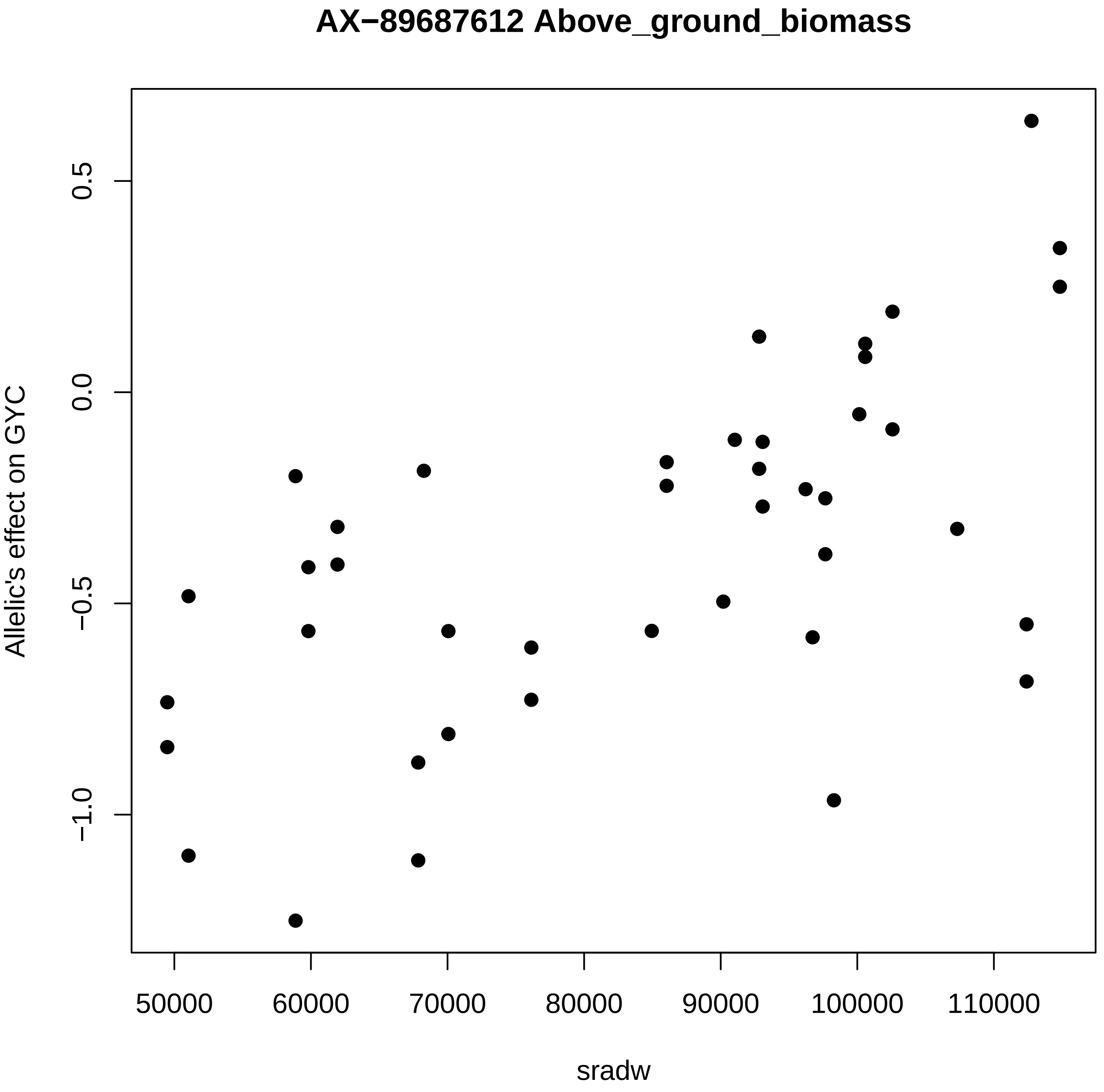
AX−89687612 Above_ground_biomass
Allelic's effect on GYC
0.5
0.0
−0.5
−1.0
50000
60000
70000
80000
90000
100000
110000
sradw

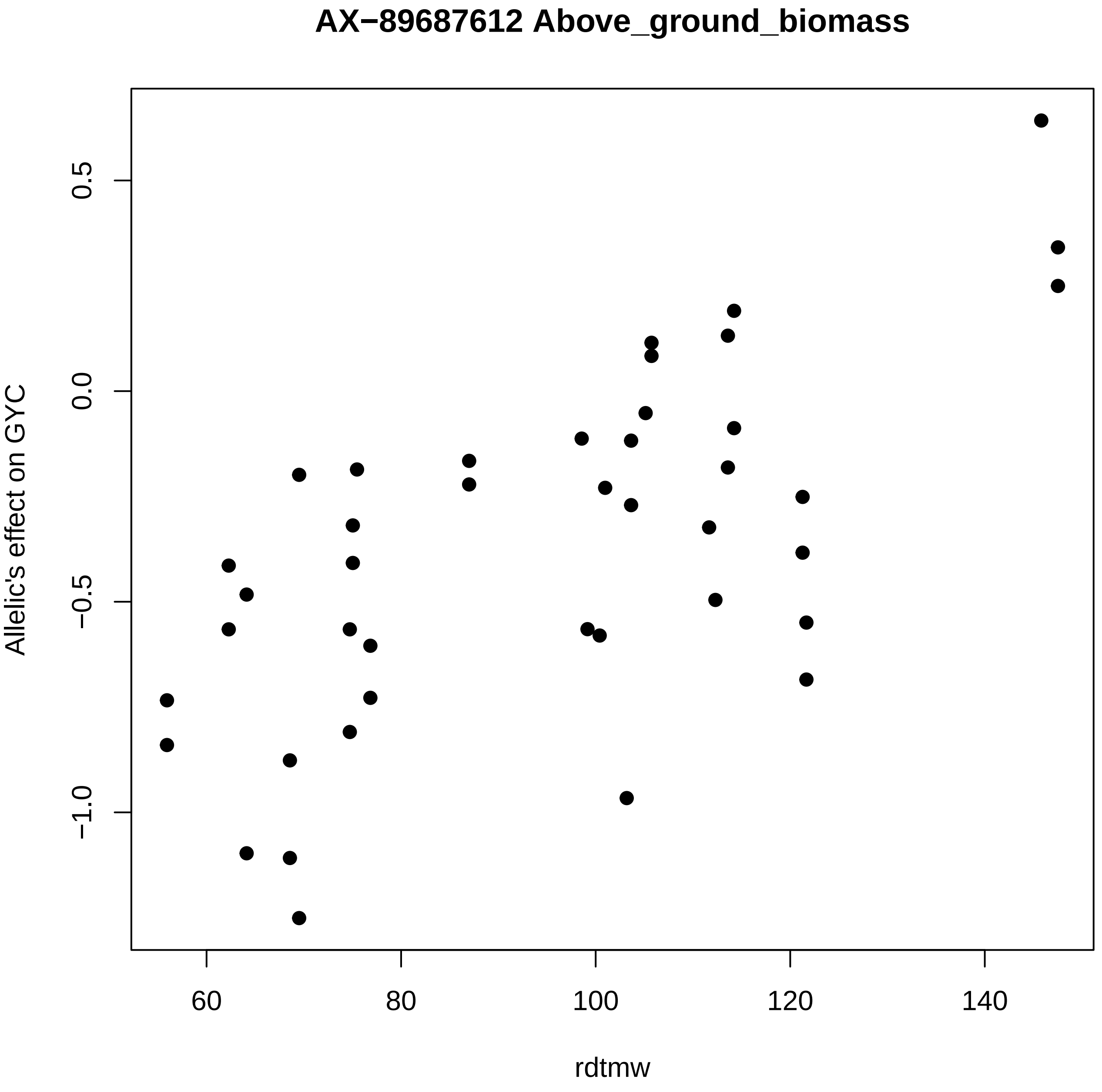

AX−89687612 Above_ground_biomass
Allelic's effect on GYC
0.5
0.0
−0.5
−1.0
60
80
100
120
140
rdtmw

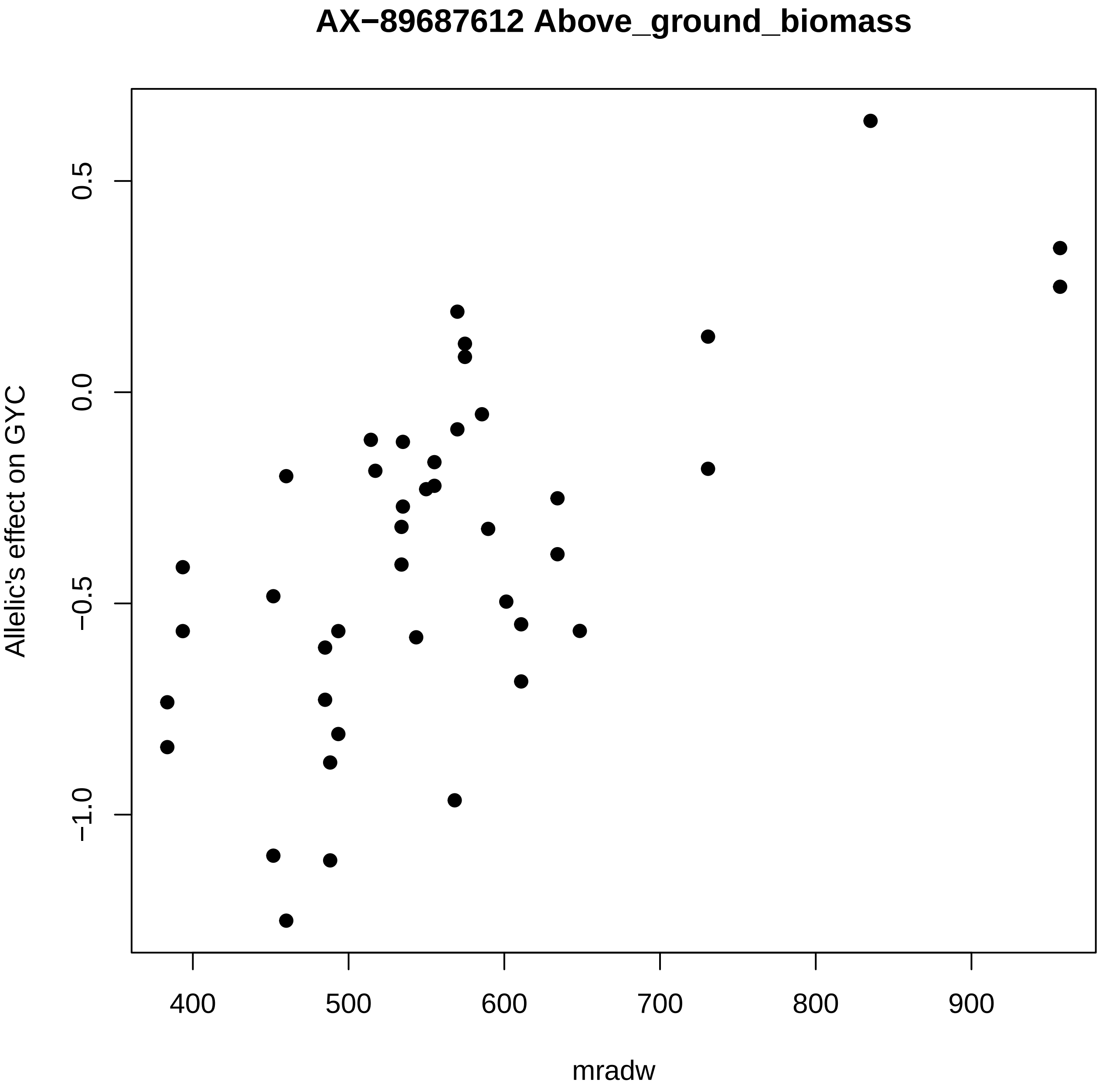

AX−89687612 Above_ground_biomass
Allelic's effect on GYC
mradw
0.5
0.0
−0.5
−1.0
400
500
600
700
800
900

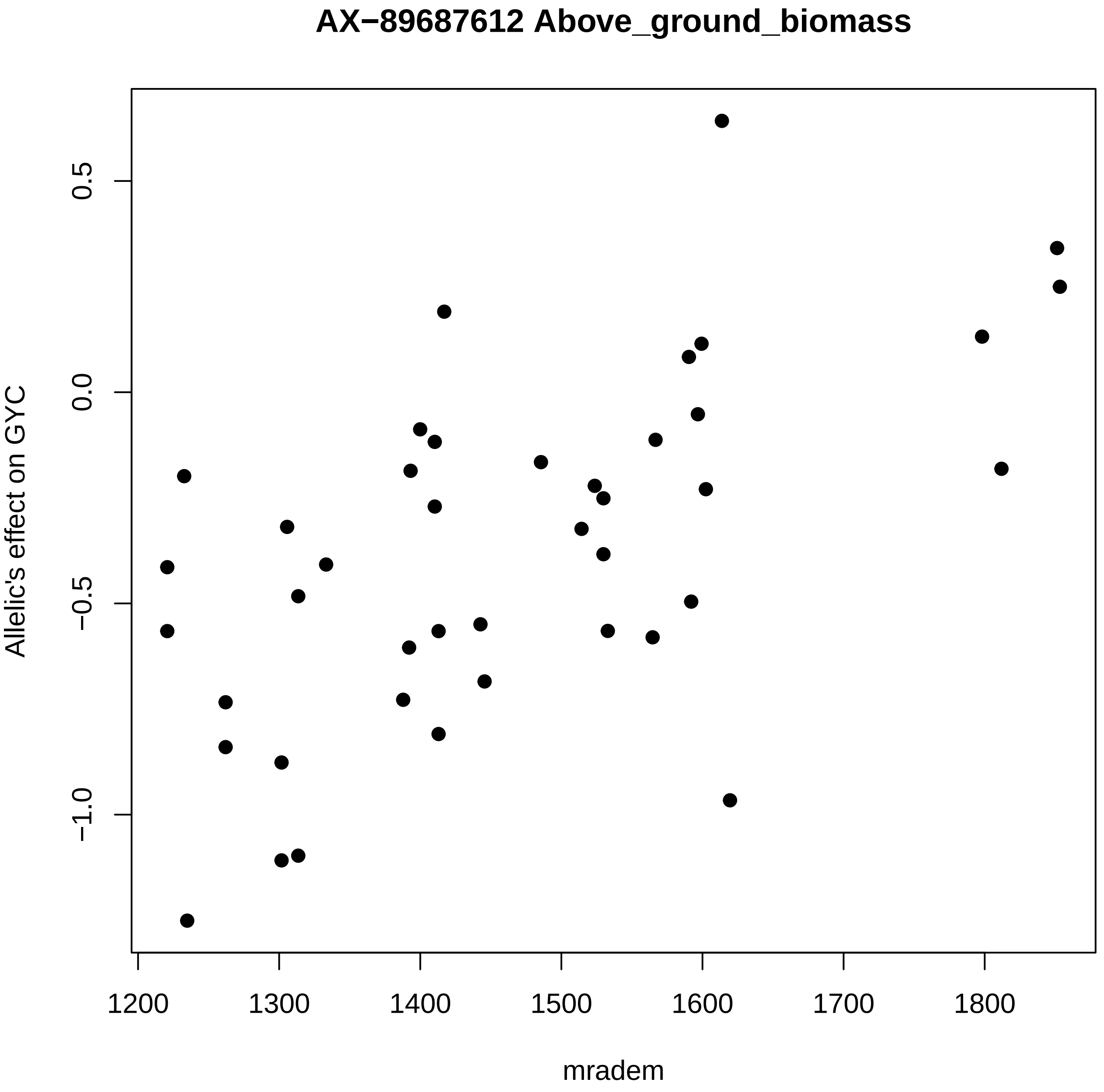
AX−89687612 Above_ground_biomass
Allelic's effect on GYC
mradem
0.5
0.0
−0.5
−1.0
1200
1300
1400
1500
1600
1700
1800

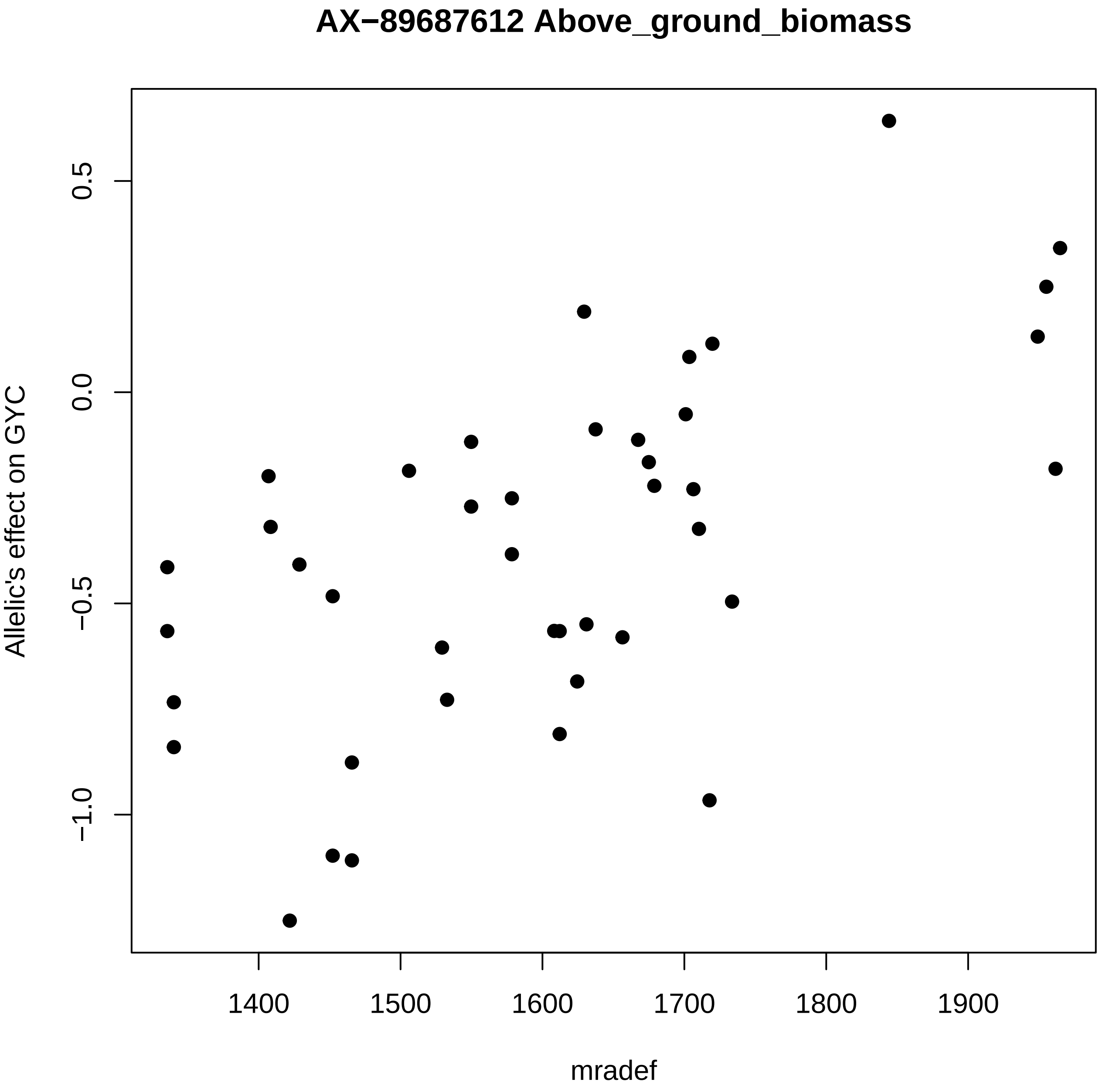
AX−89687612 Above_ground_biomass
Allelic's effect on GYC
mradef
0.5
0.0
−0.5
−1.0
1400
1500
1600
1700
1800
1900

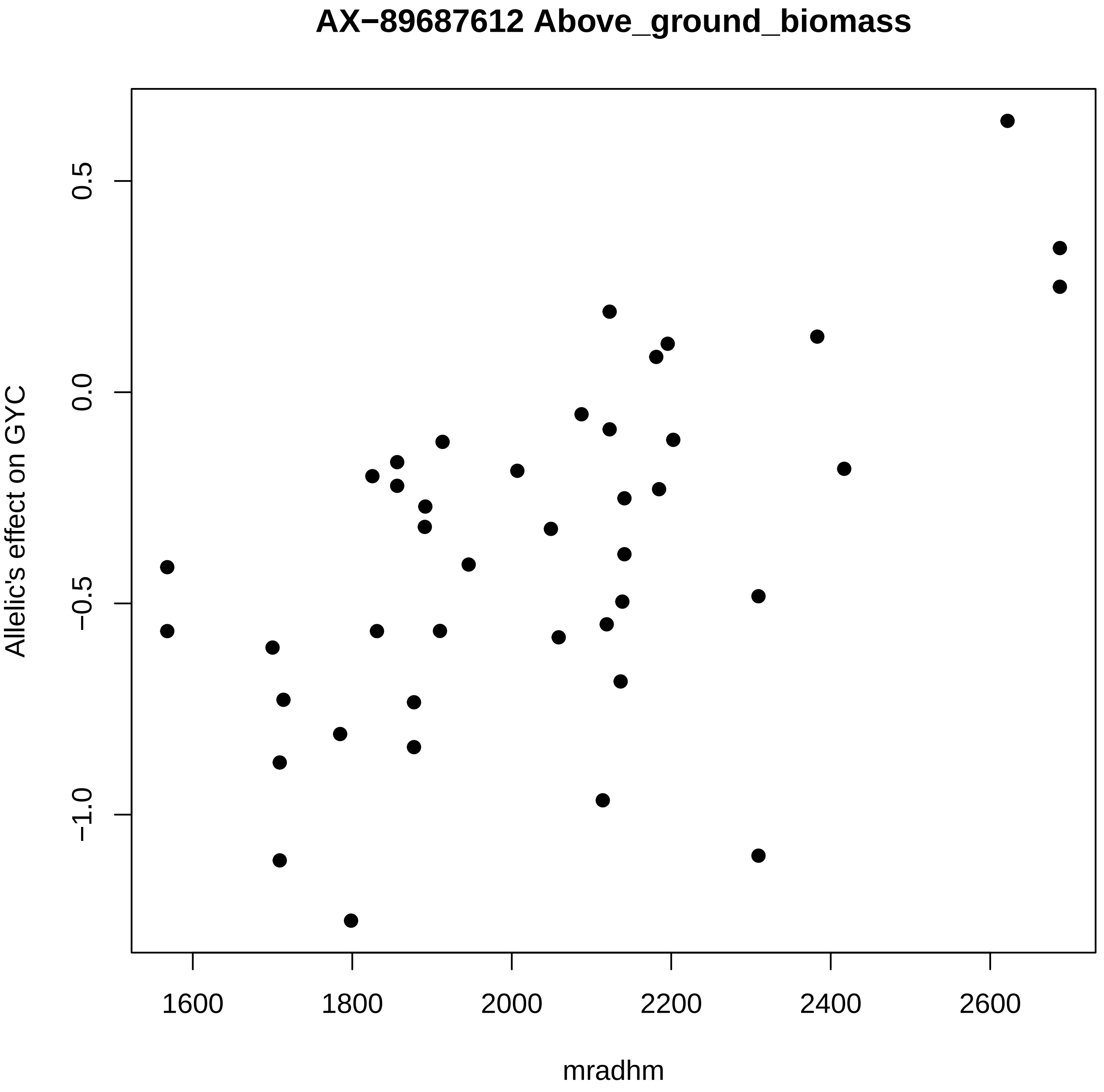

AX−89687612 Above_ground_biomass
Allelic's effect on GYC
mradhm
0.5
0.0
−0.5
−1.0
1600
1800
2000
2200
2400
2600

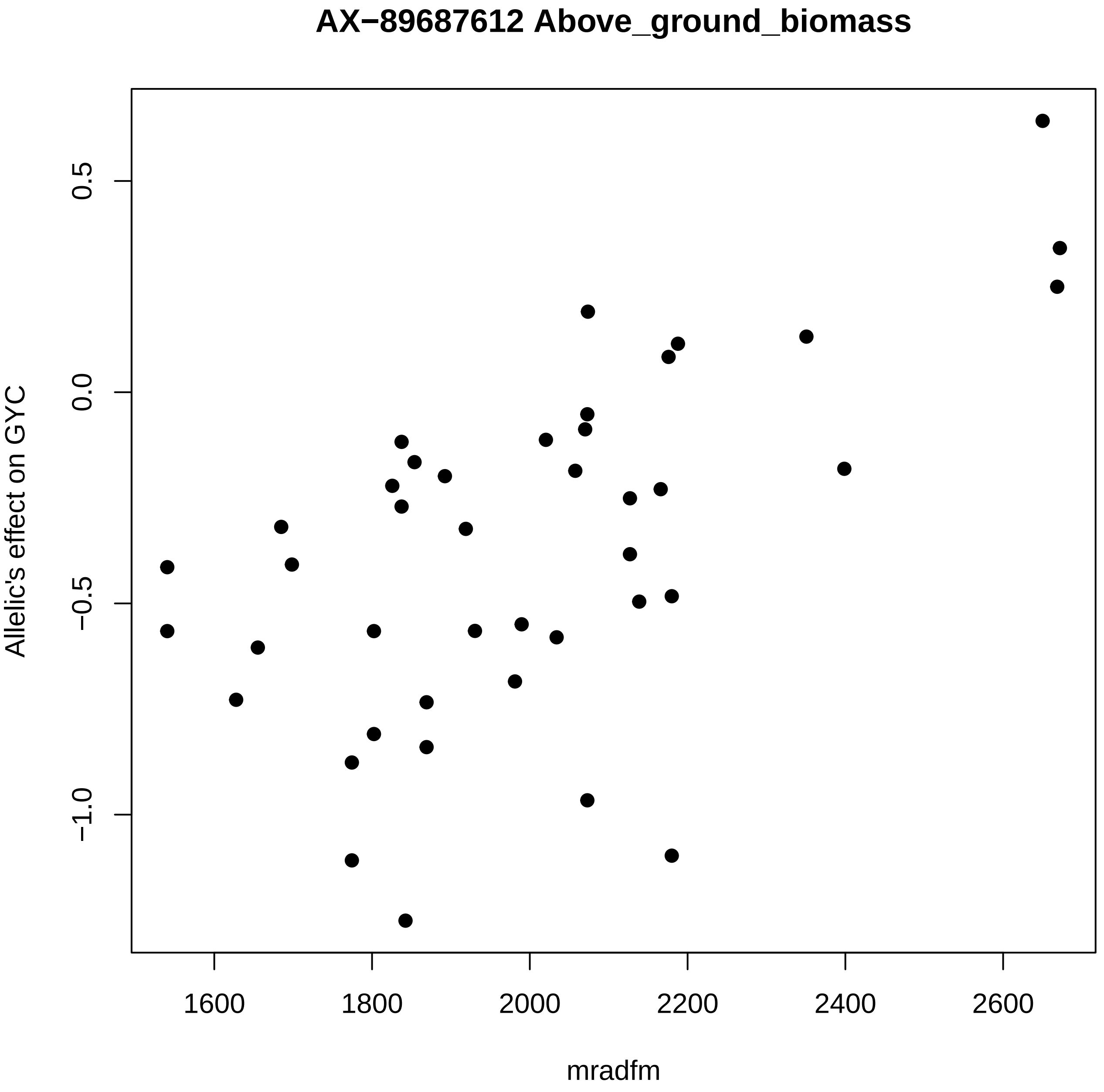

AX−89687612 Above_ground_biomass
Allelic's effect on GYC
mradfm
0.5
0.0
−0.5
−1.0
1600
1800
2000
2200
2400
2600

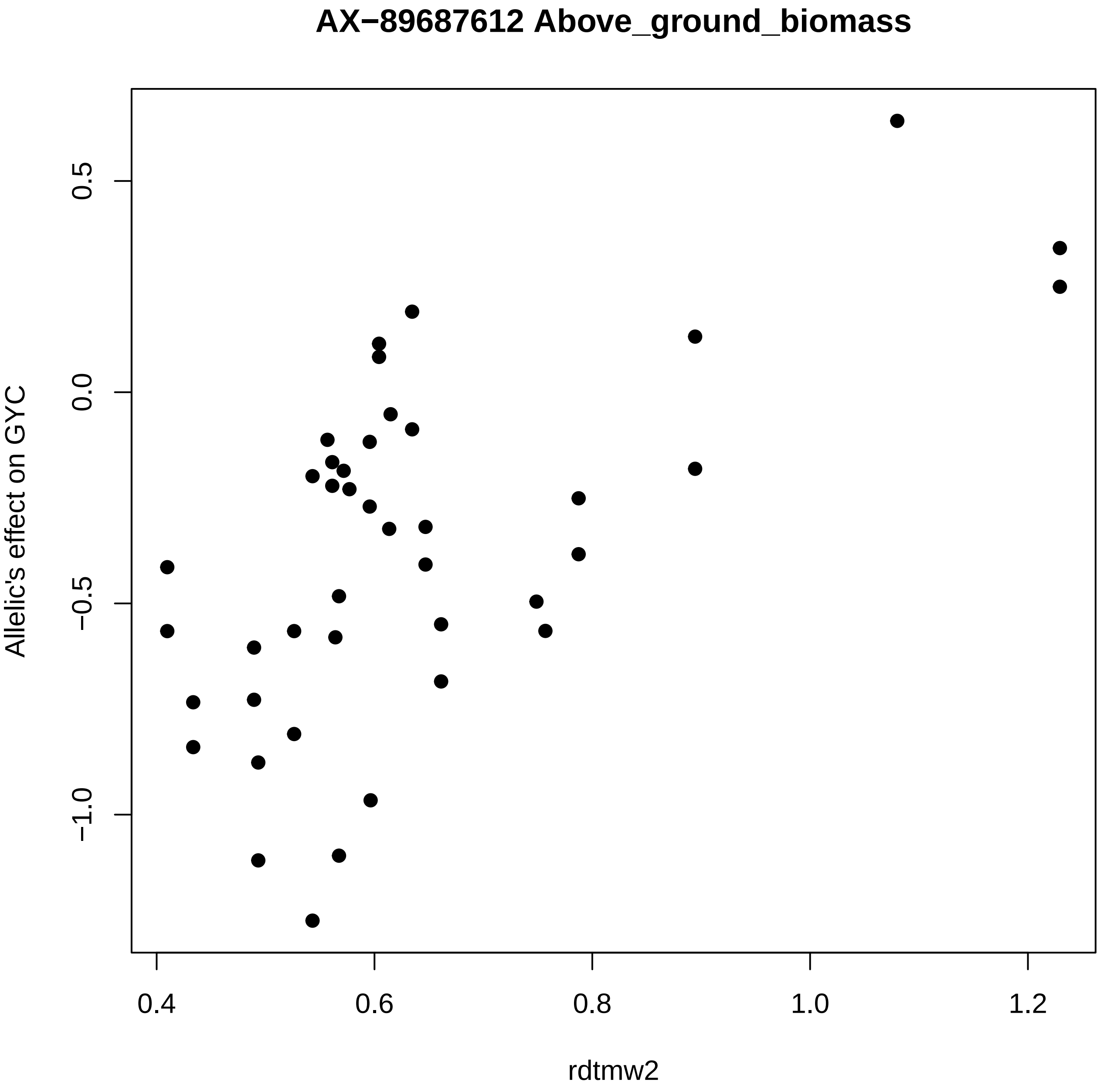
AX−89687612 Above_ground_biomass
Allelic's effect on GYC
rdtmw2
0.5
0.0
−0.5
−1.0
0.4
0.6
0.8
1.0
1.2

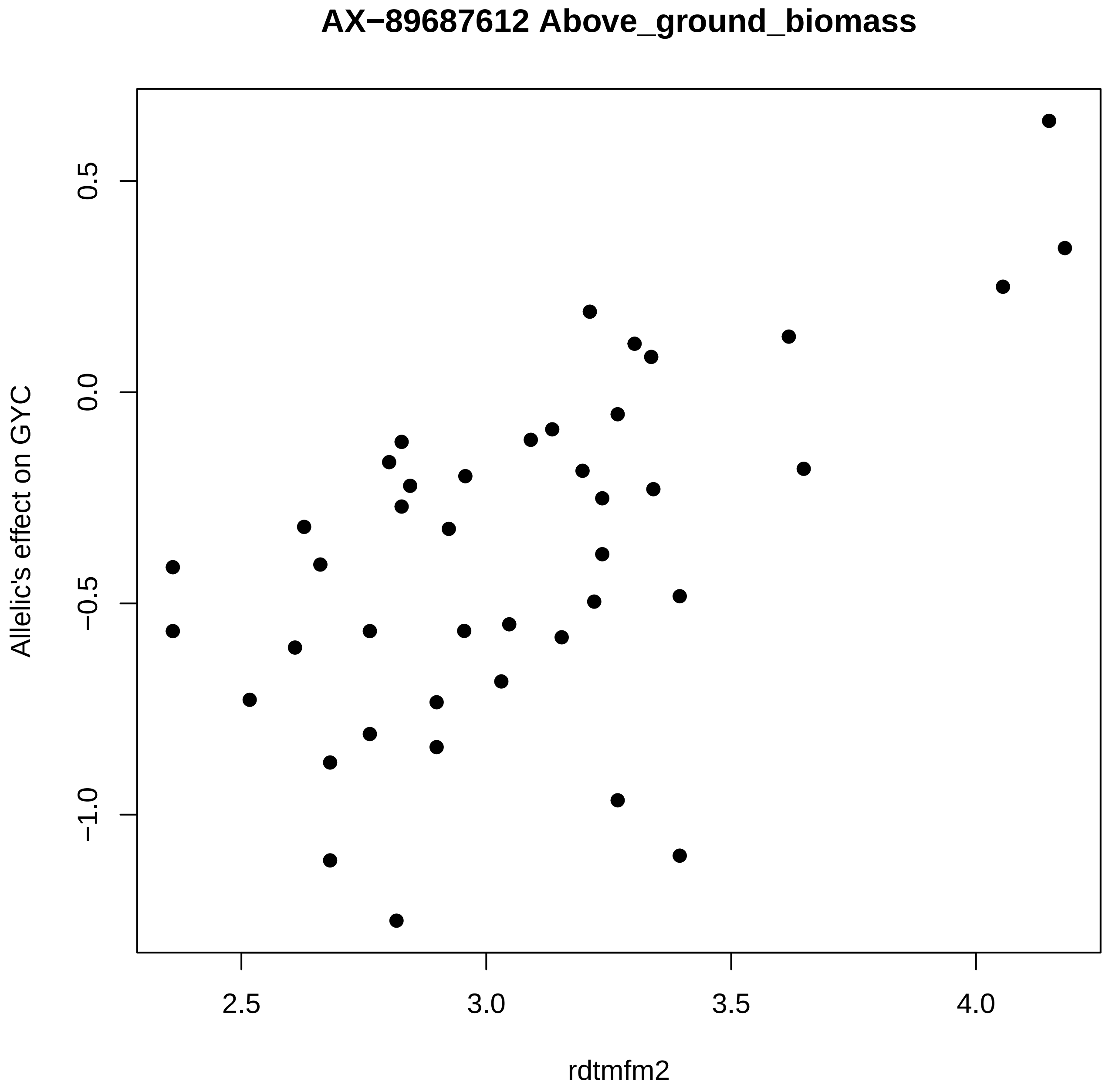

AX−89687612 Above_ground_biomass
0.5
0.0
−0.5
−1.0
Allelic's effect on GYC
2.5
3.0
3.5
4.0
rdtmfm2

AX−89687612 Root_biomass

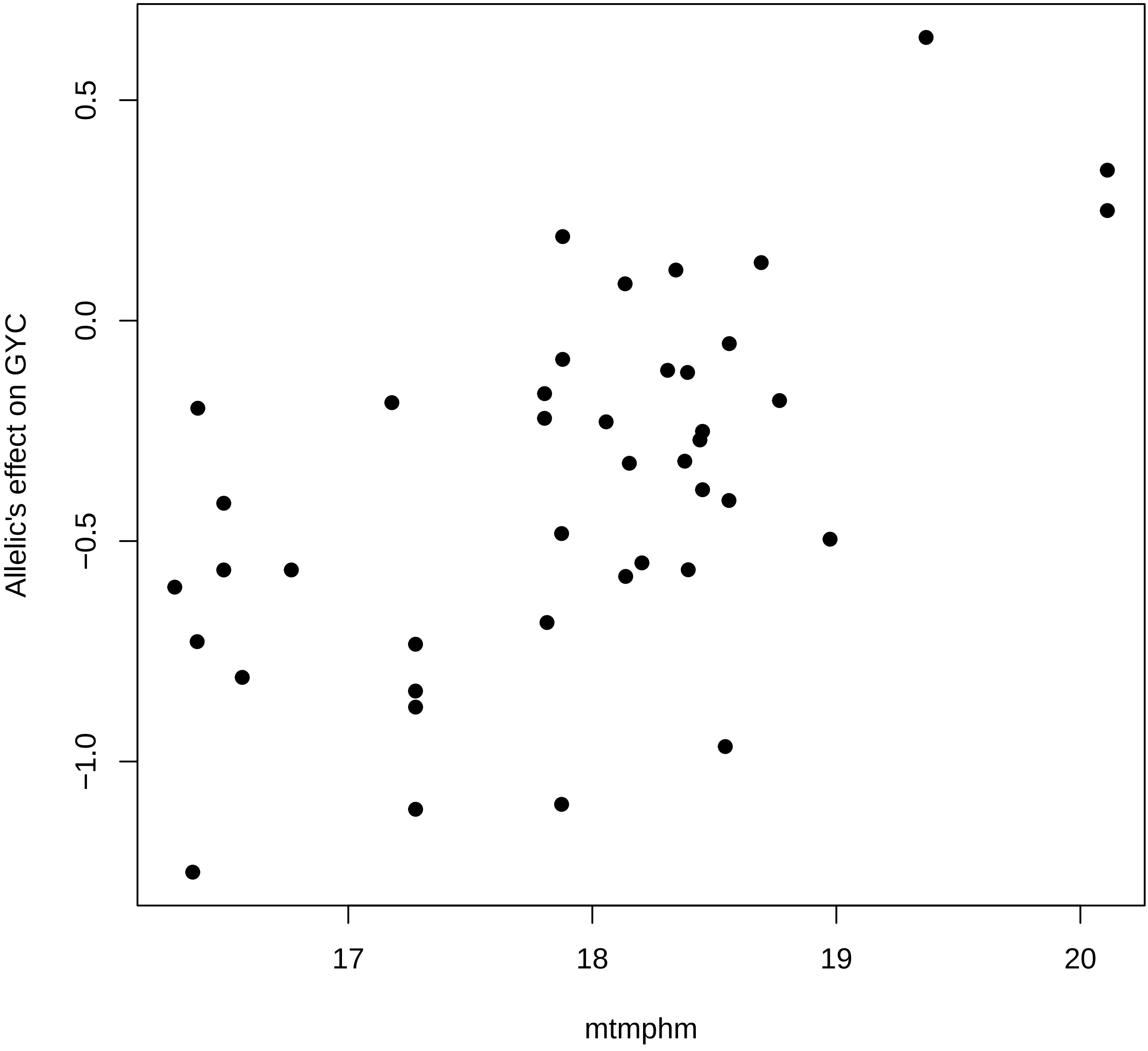

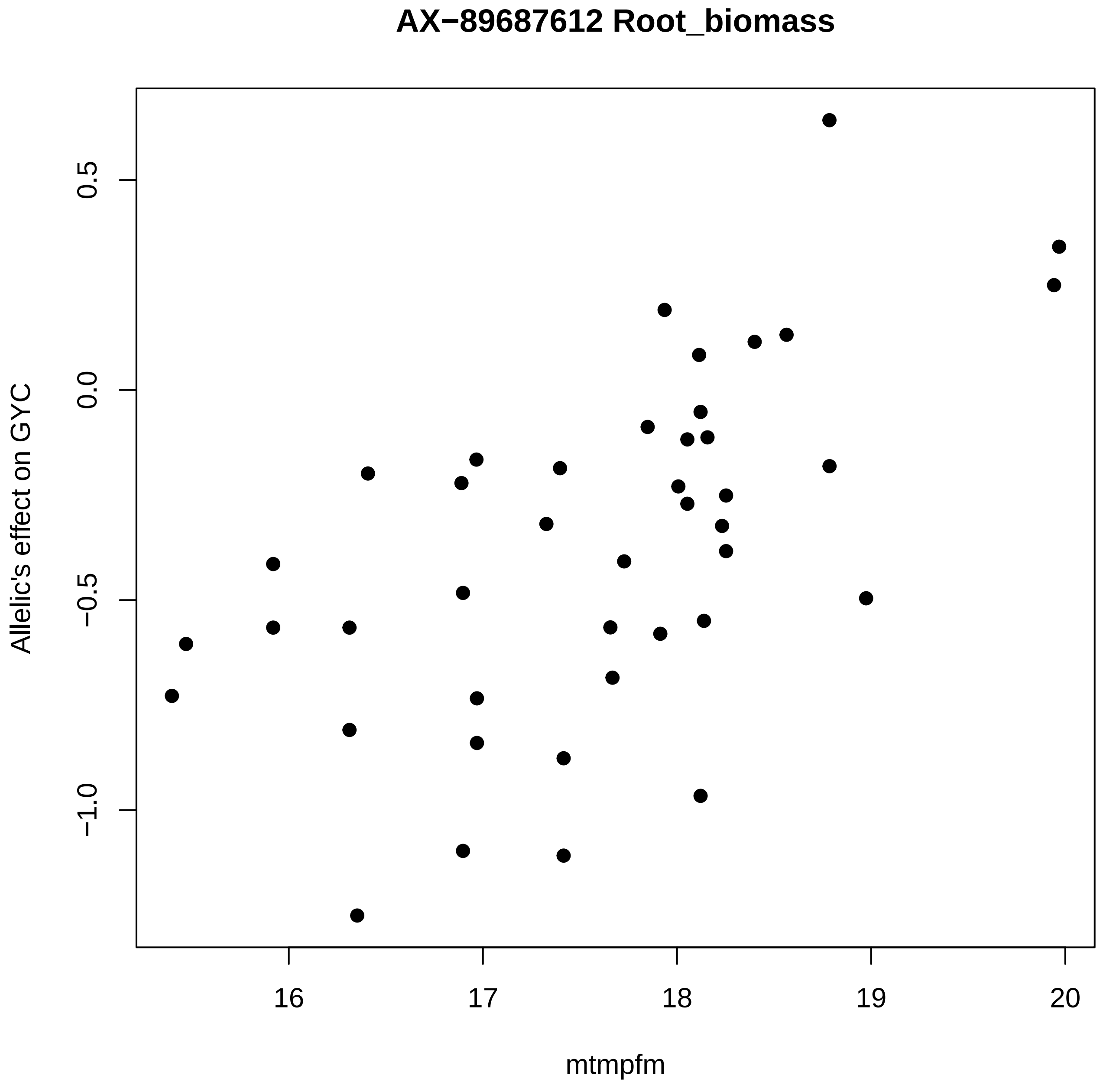

AX−89687612 Root_biomass
Allelic's effect on GYC
0.5
0.0
−0.5
−1.0
16
17
18
19
20
mtmpfm

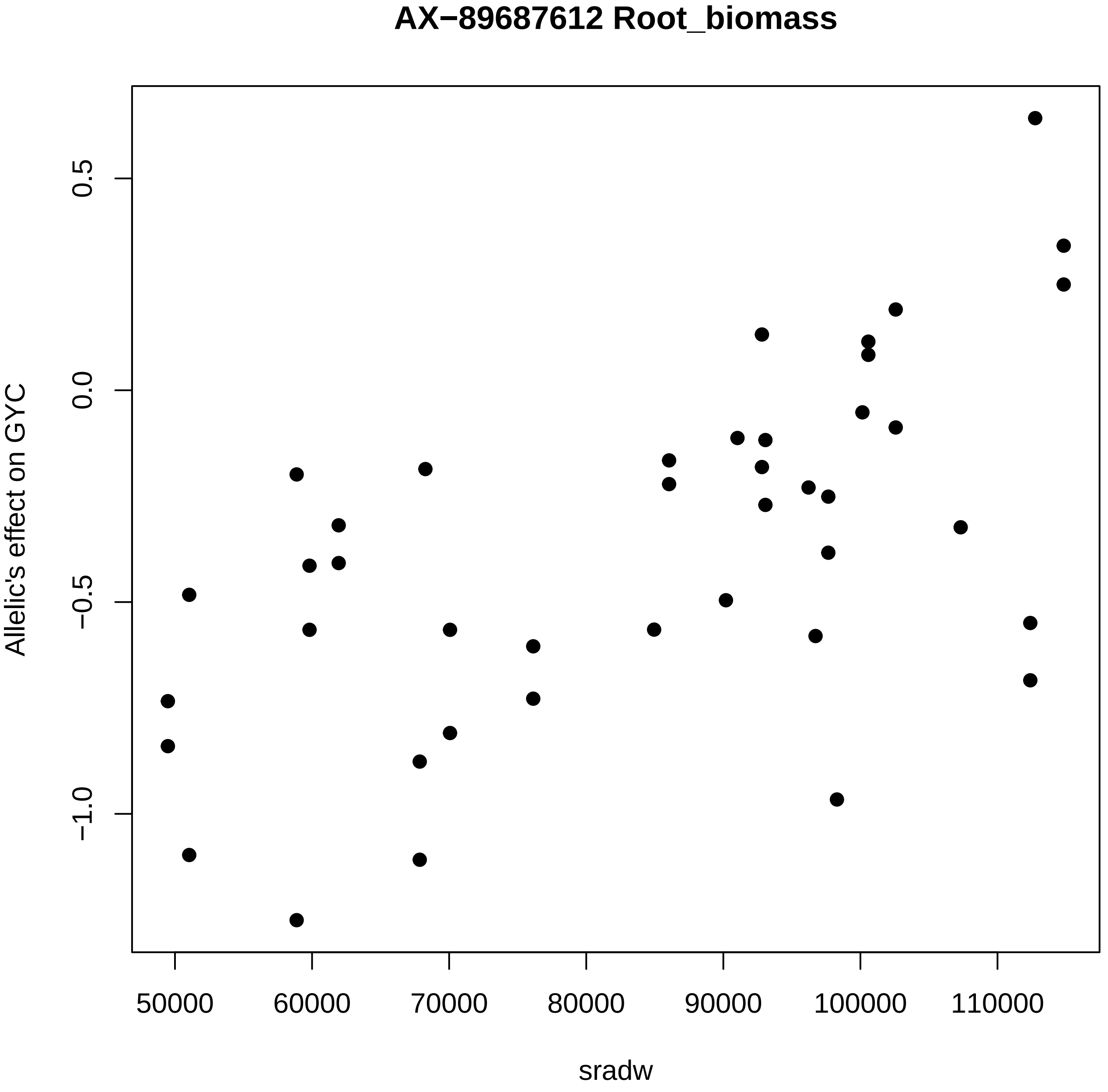
AX−89687612 Root_biomass
Allelic's effect on GYC
0.5
0.0
−0.5
−1.0
50000
60000
70000
80000
90000
100000
110000
sradw

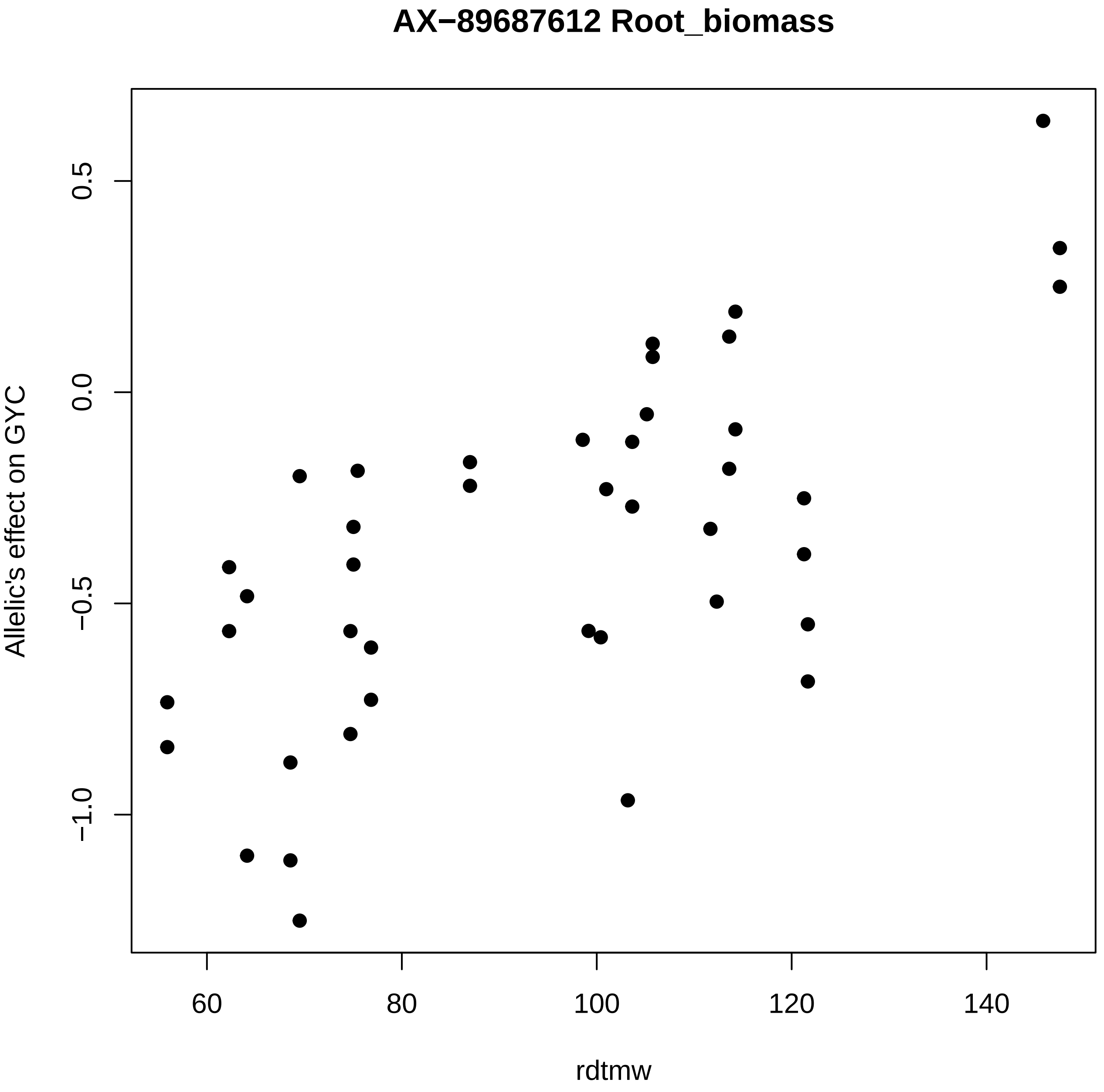
AX−89687612 Root_biomass
Allelic's effect on GYC
0.5
0.0
−0.5
−1.0
60
80
100
120
140
rdtmw

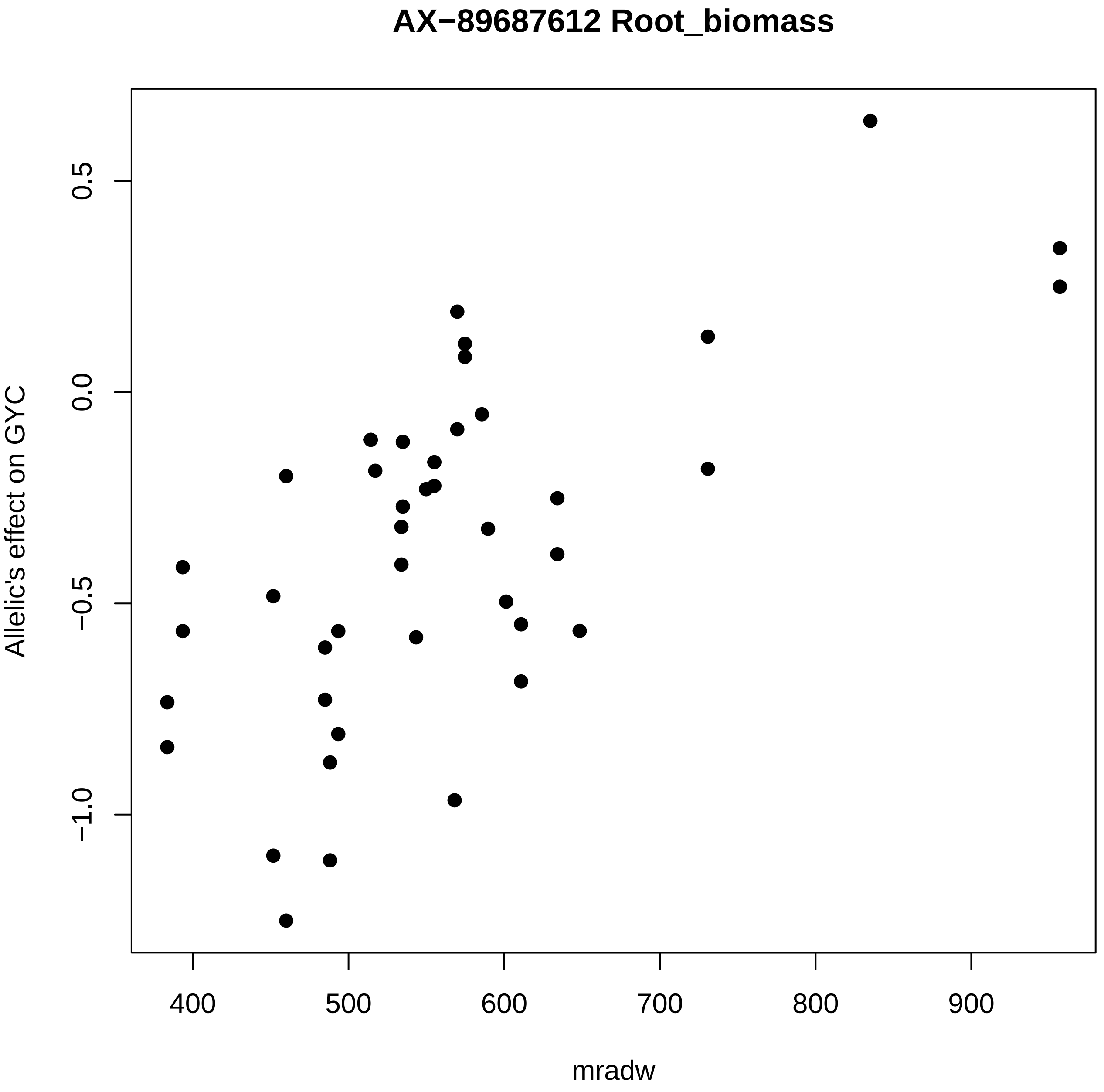
AX−89687612 Root_biomass
Allelic's effect on GYC
0.5
0.0
−0.5
−1.0
400
500
600
700
800
900
mradw

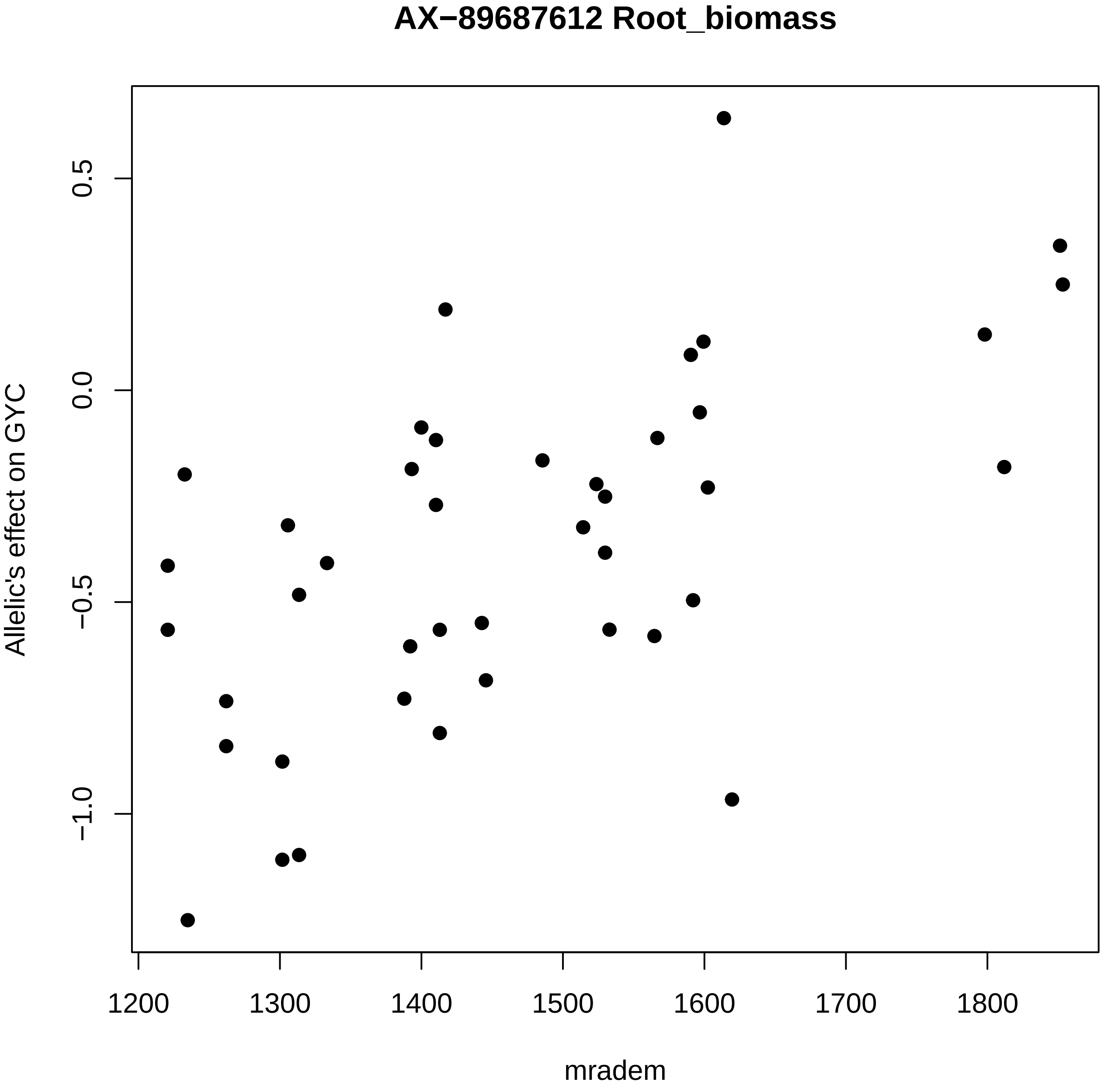
AX−89687612 Root_biomass
Allelic's effect on GYC
mradem
0.5
0.0
−0.5
−1.0
1200
1300
1400
1500
1600
1700
1800

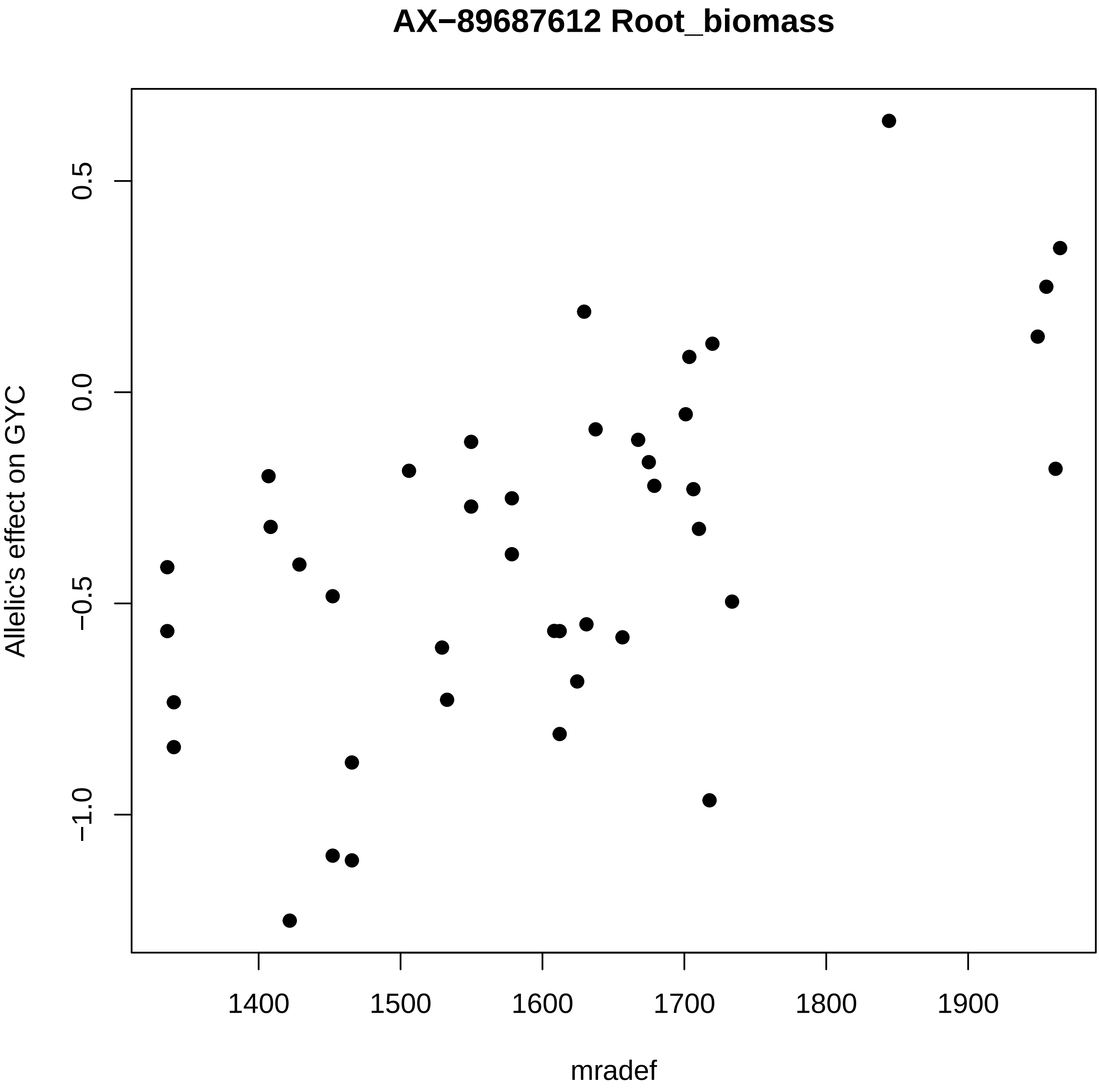

AX−89687612 Root_biomass
0.5
0.0
−0.5
−1.0
Allelic's effect on GYC
1400
1500
1600
1700
1800
1900
mradef

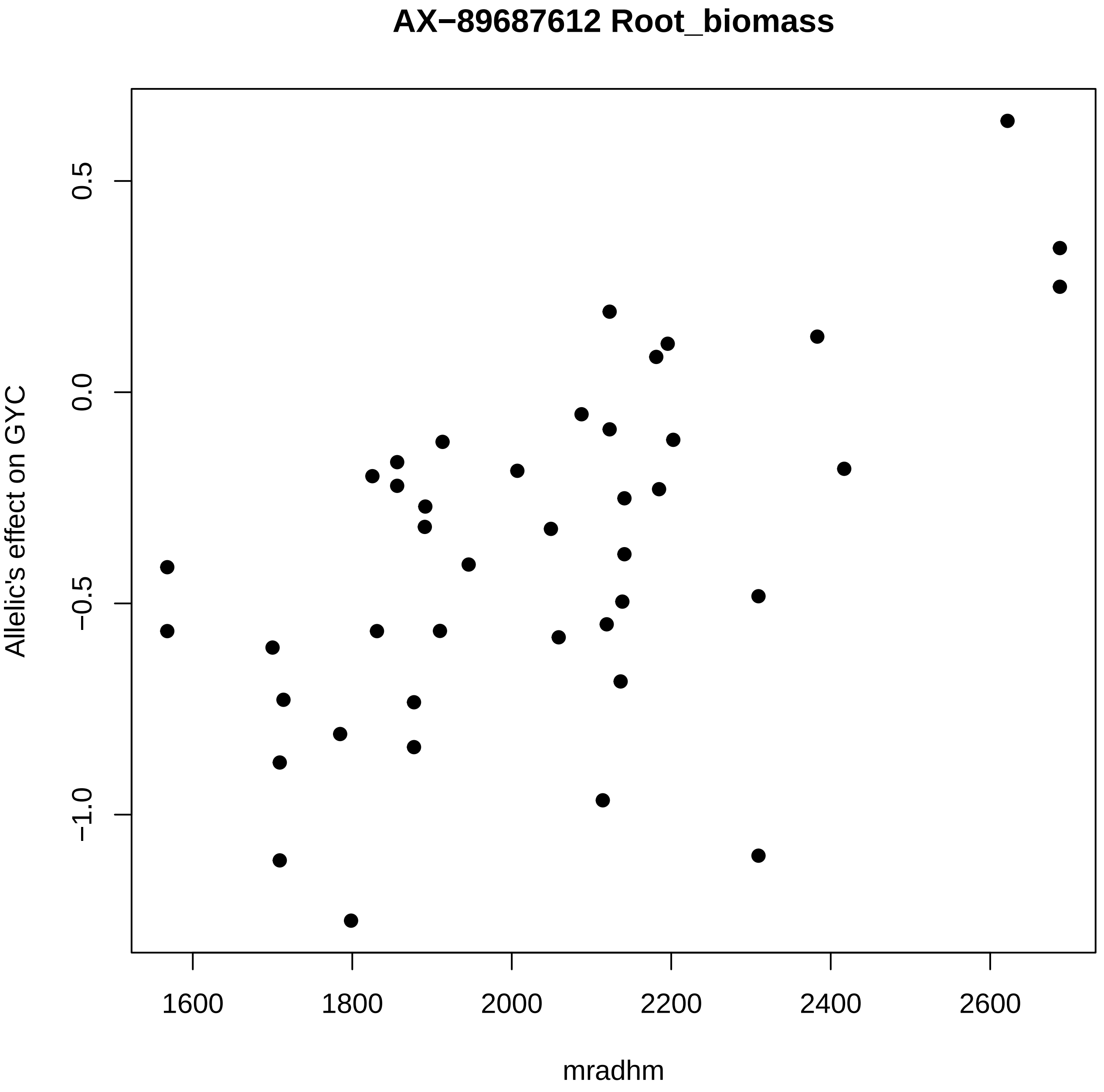

AX−89687612 Root_biomass
Allelic's effect on GYC
0.5
0.0
−0.5
−1.0
1600
1800
2000
2200
2400
2600
mradhm

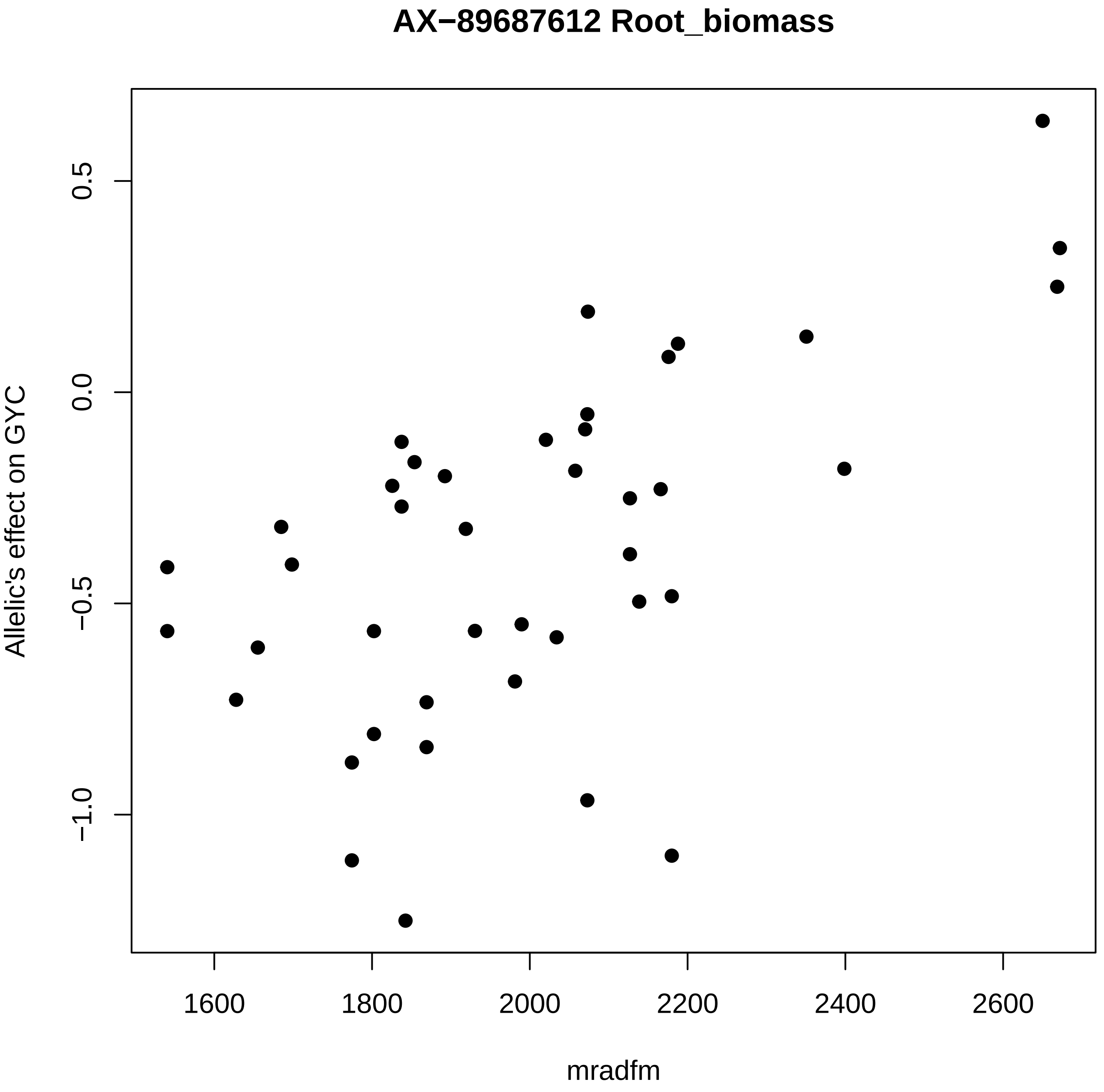
AX−89687612 Root_biomass
Allelic's effect on GYC
0.5
0.0
−0.5
−1.0
1600
1800
2000
2200
2400
2600
mradfm

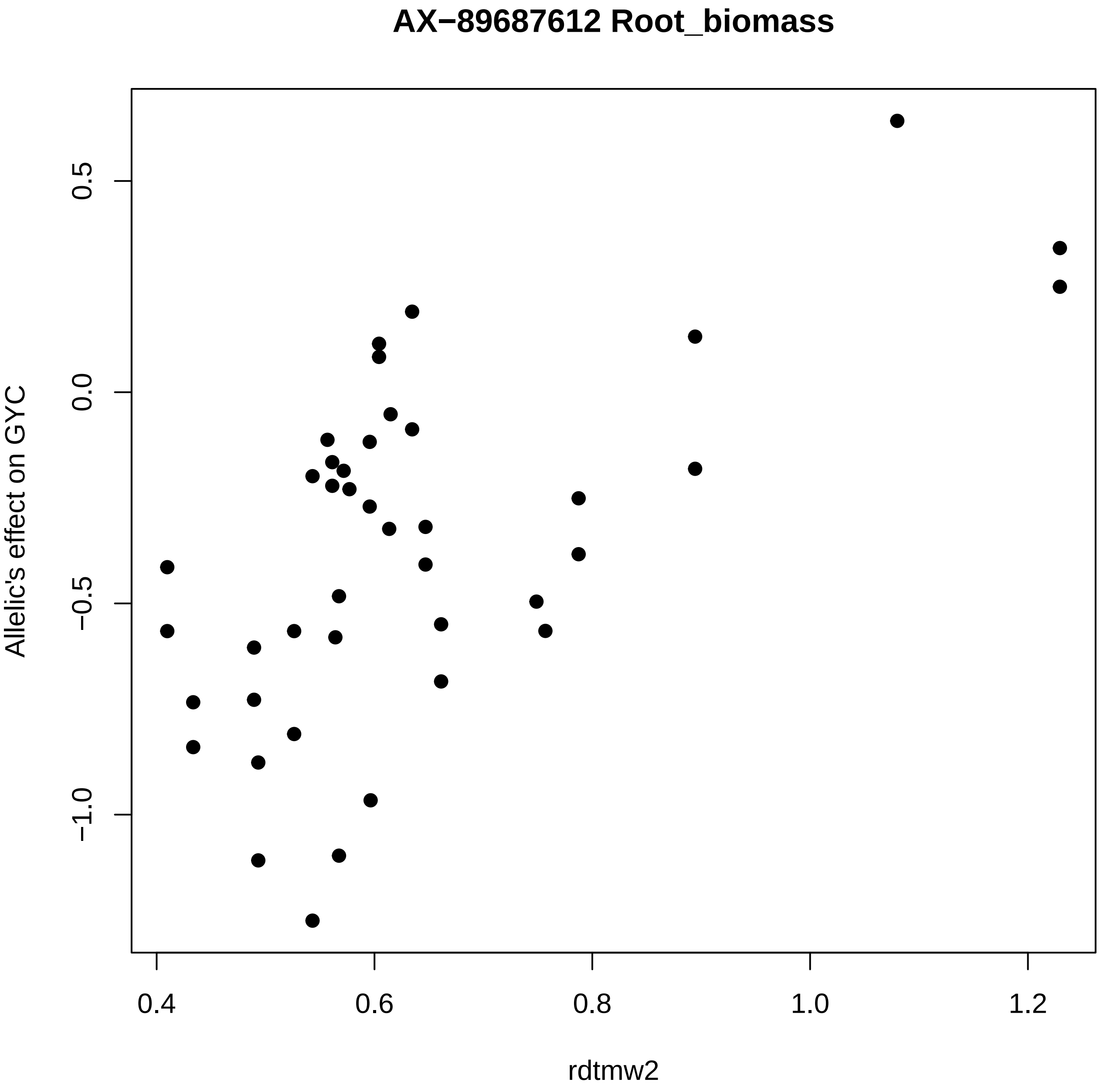

AX−89687612 Root_biomass
0.5
0.0
−0.5
−1.0
Allelic's effect on GYC
0.4
0.6
0.8
1.0
1.2
rdtmw2

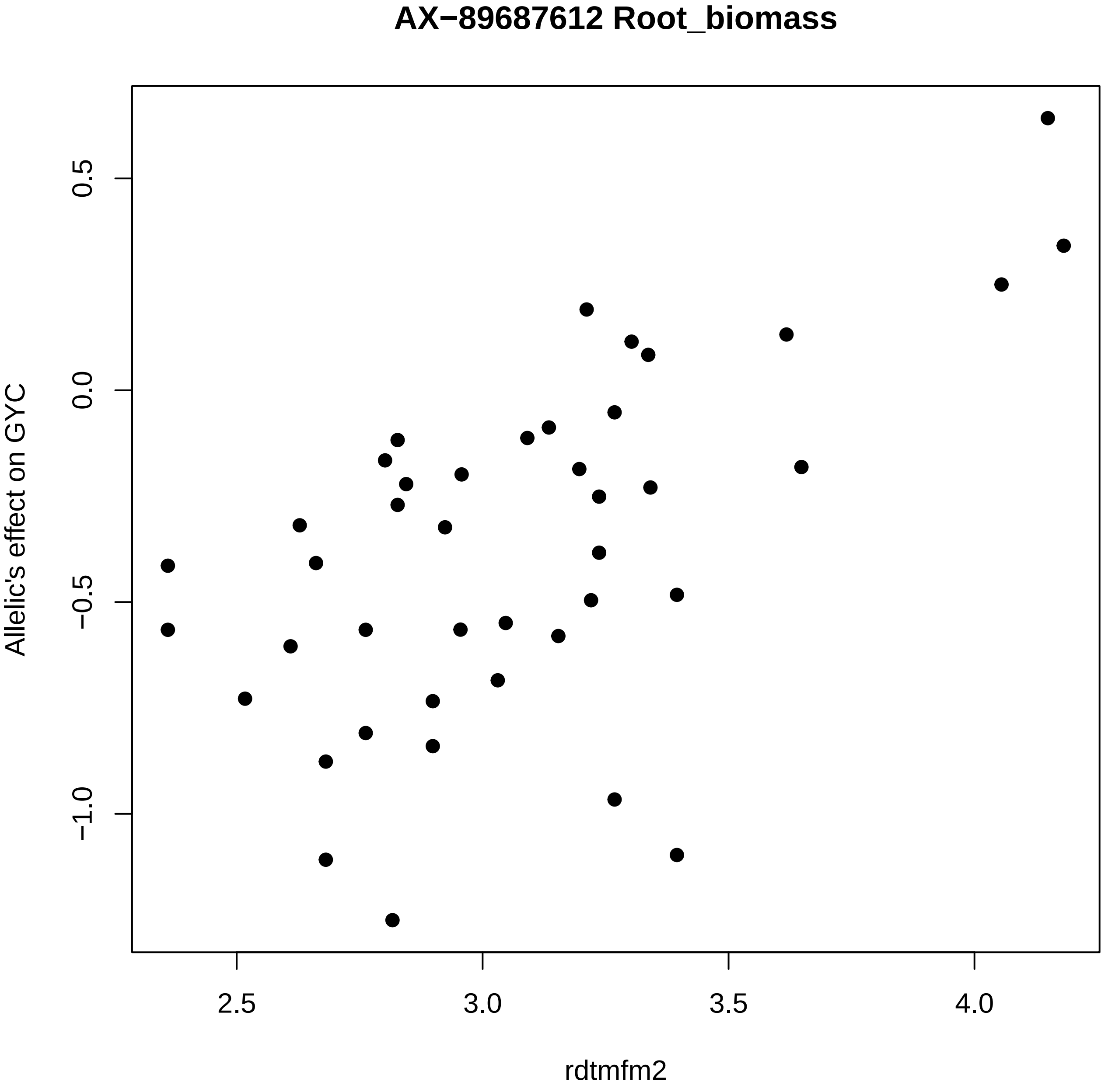
AX−89687612 Root_biomass
Allelic's effect on GYC
0.5
0.0
−0.5
−1.0
2.5
3.0
3.5
4.0
rdtmfm2

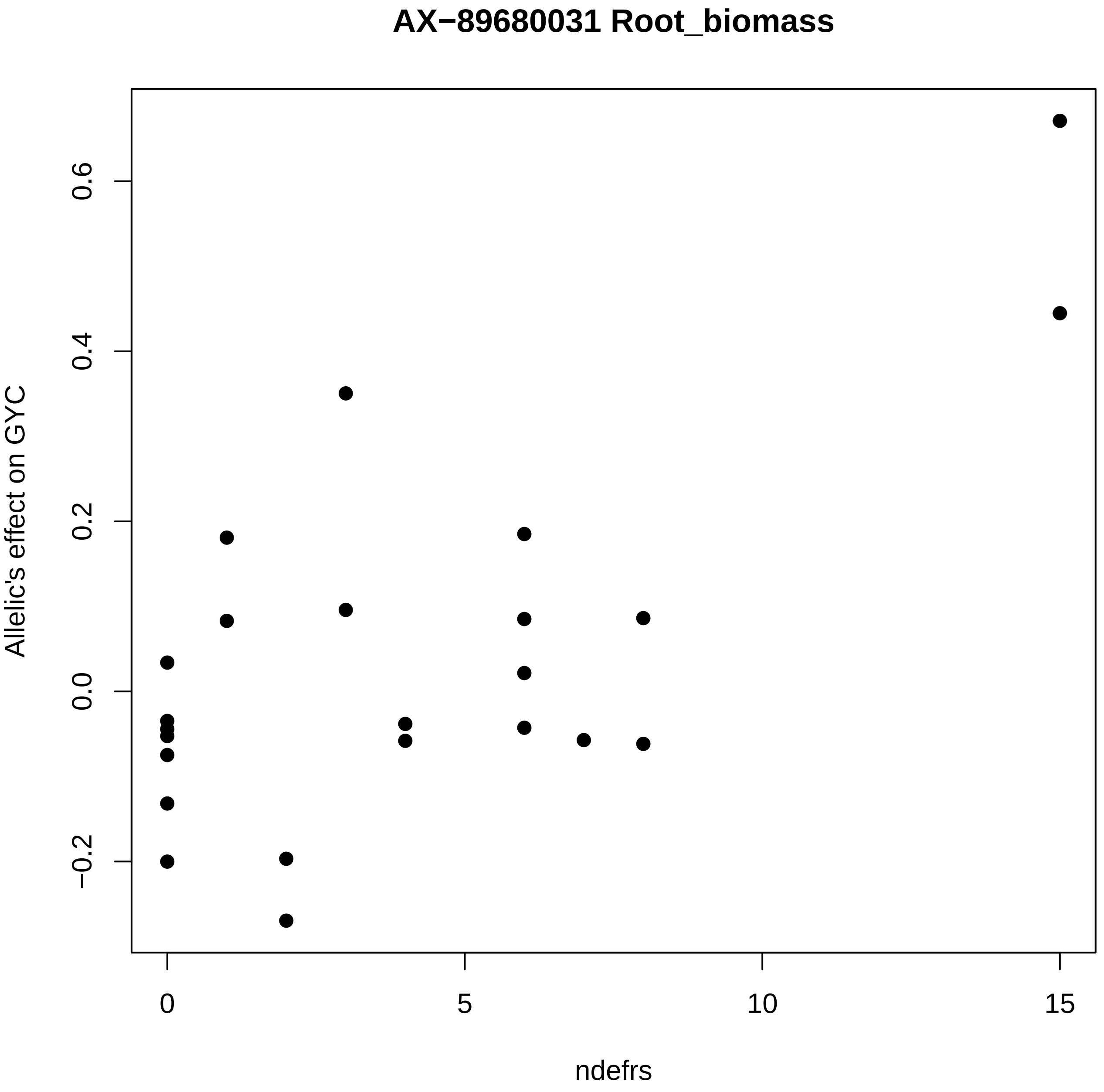

AX−89680031 Root_biomass
Allelic's effect on GYC
ndefrs
0.6
0.4
0.2
0.0
−0.2
0
5
10
15

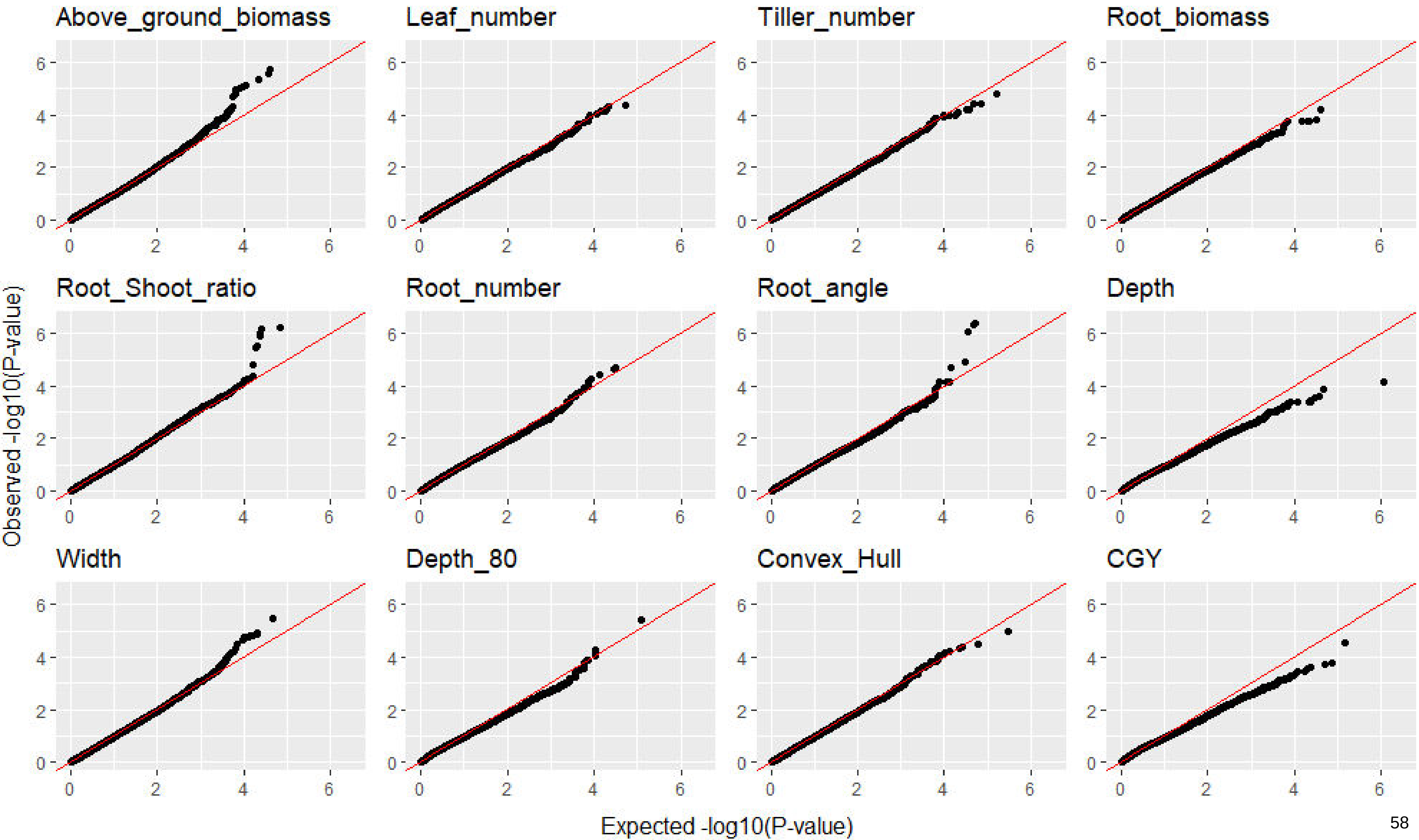
Above_ground_biomass
Leaf_number
Tiller_number
Root_biomass
Root_Shoot_ratio
Root_number
Root_angle
Depth
Width
Depth_80
Convex_Hull
CGY
Observed -log10(P-value)
Expected -log10(P-value)

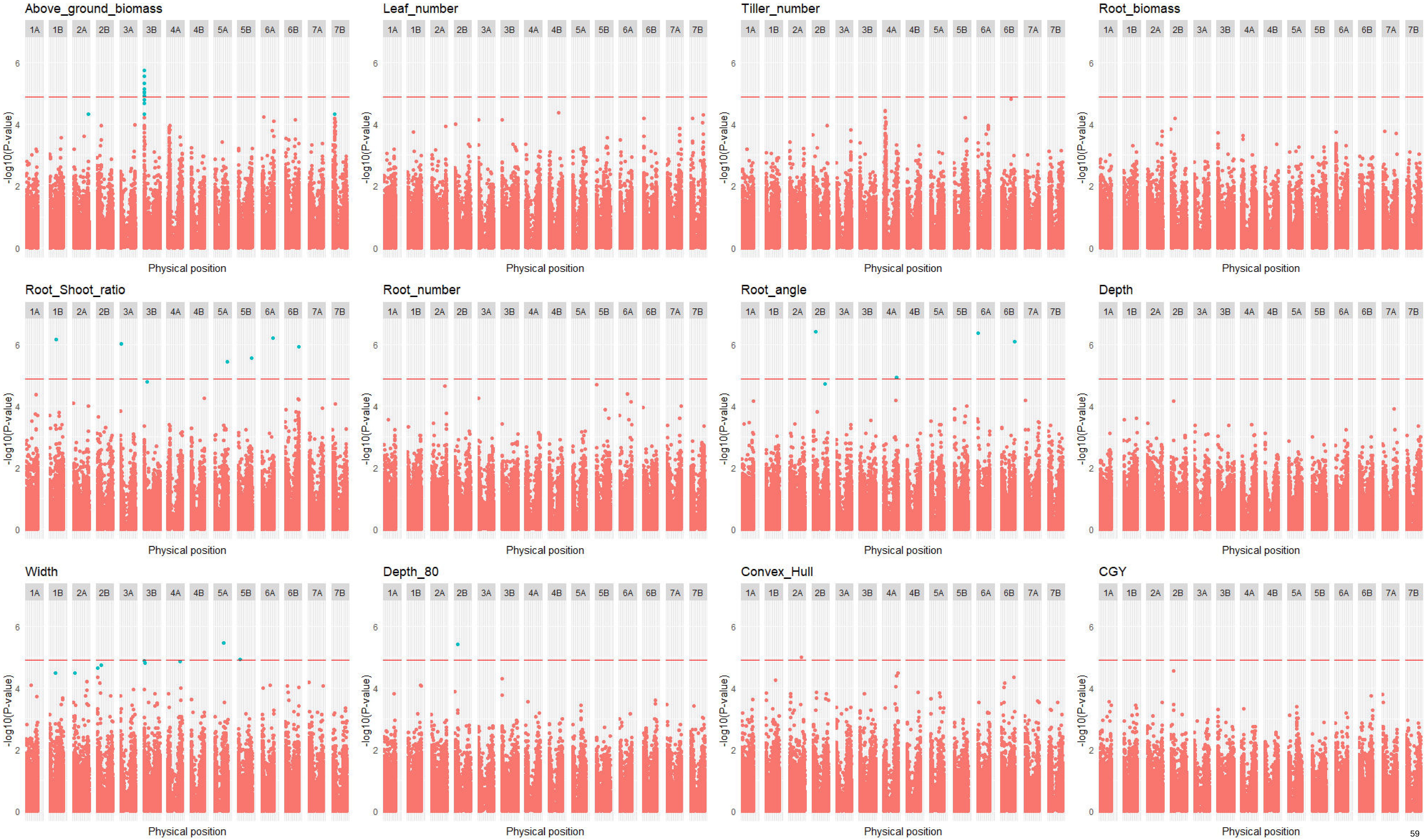
Above_ground_biomass
Leaf_number
Tiller_number
Root_biomass
Root_Shoot_ratio
Root_number
Root_angle
Depth
Width
Depth_80
Convex_Hull
CGY
1A 1B 2A 2B 3A 3B 4A 4B 5A 5B 6A 6B 7A 7B
-log10(P-value)
Physical position

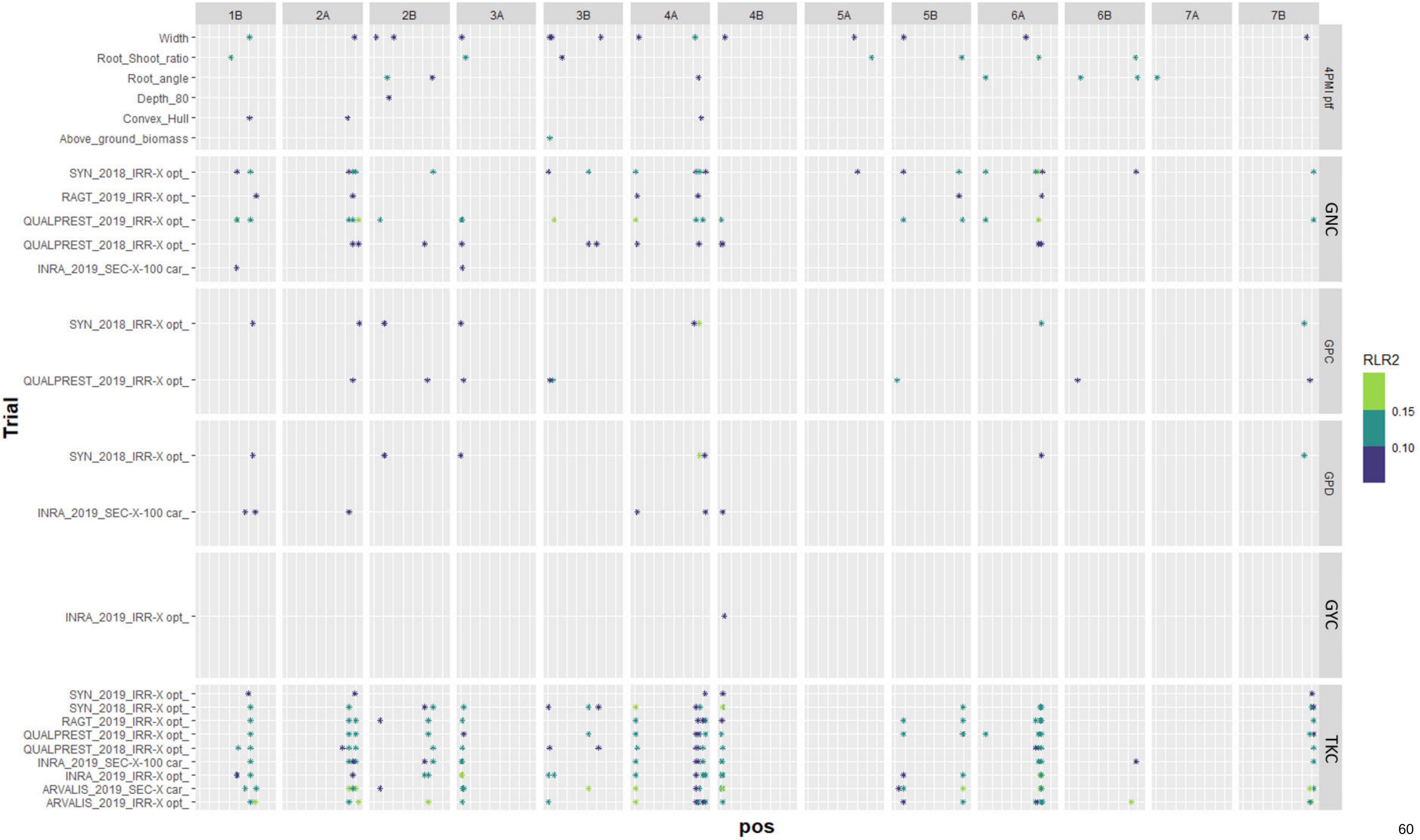
1B
2A
2B
3A
3B
4A
4B
5A
5B
6A
6B
7A
7B
Width
Root_Shoot_ratio
Root_angle
Depth_80
Convex_Hull
Above_ground_biomass
4PMI ptf
SYN_2018_IRR-X opt_
RAGT_2019_IRR-X opt_
QUALPREST_2019_IRR-X opt_
QUALPREST_2018_IRR-X opt_
INRA_2019_SEC-X-100 car_
GNC
SYN_2018_IRR-X opt_
QUALPREST_2019_IRR-X opt_
GPC
SYN_2018_IRR-X opt_
INRA_2019_SEC-X-100 car_
GPD
INRA_2019_IRR-X opt_
GYC
SYN_2019_IRR-X opt_
SYN_2018_IRR-X opt_
RAGT_2019_IRR-X opt_
QUALPREST_2019_IRR-X opt_
QUALPREST_2018_IRR-X opt_
INRA_2019_SEC-X-100 car_
INRA_2019_IRR-X opt_
ARVALIS_2019_SEC-X car_
ARVALIS_2019_IRR-X opt_
TKC
Trial
pos
RLR2
0.15
0.10

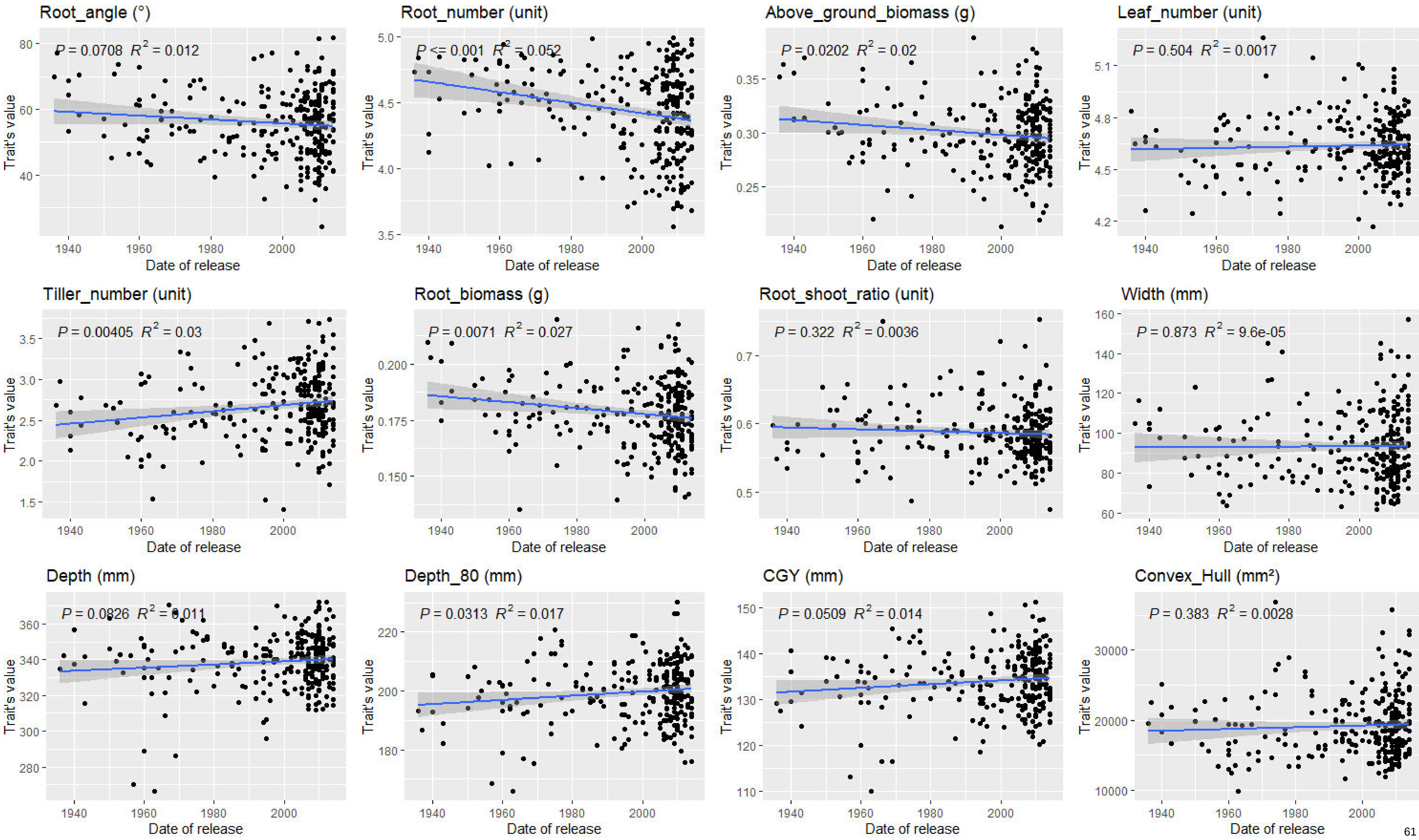
Root_angle (°)
P = 0.0708 R² = 0.012
Root_number (unit)
P <= 0.001 R² = 0.052
Above_ground_biomass (g)
P = 0.0202 R² = 0.02
Leaf_number (unit)
P = 0.504 R² = 0.0017
Tiller_number (unit)
P = 0.00405 R² = 0.03
Root_biomass (g)
P = 0.0071 R² = 0.027
Root_shoot_ratio (unit)
P = 0.322 R² = 0.0036
Width (mm)
P = 0.873 R² = 9.6e-05
Depth (mm)
P = 0.0826 R² = 0.011
Depth_80 (mm)
P = 0.0313 R² = 0.017
CGY (mm)
P = 0.0509 R² = 0.014
Convex_Hull (mm²)
P = 0.383 R² = 0.0028
Trait's value
Date of release

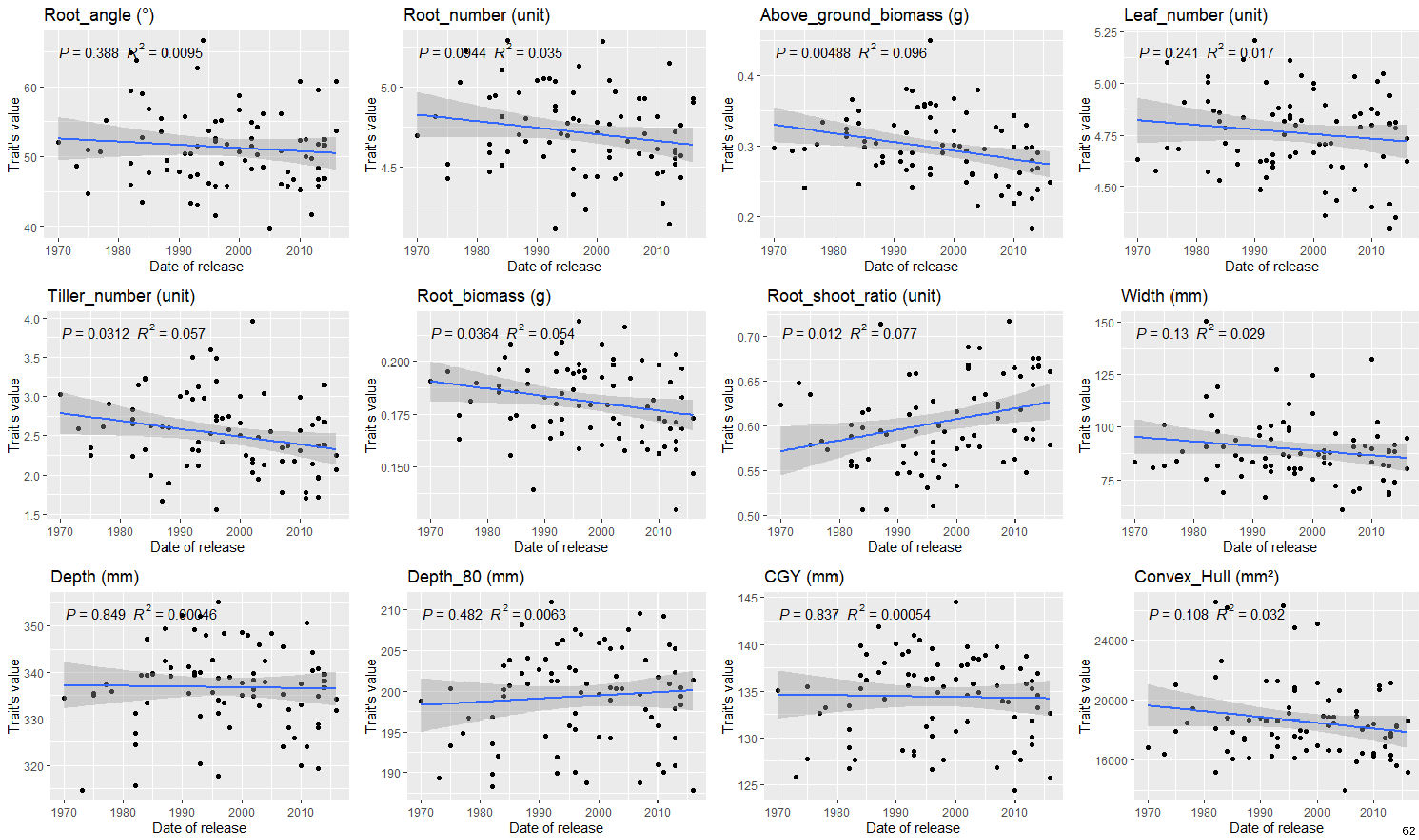
Root_angle (°)
P = 0.388 R² = 0.0095
Root_number (unit)
P = 0.0944 R² = 0.035
Above_ground_biomass (g)
P = 0.00488 R² = 0.096
Leaf_number (unit)
P = 0.241 R² = 0.017
Tiller_number (unit)
P = 0.0312 R² = 0.057
Root_biomass (g)
P = 0.0364 R² = 0.054
Root_shoot_ratio (unit)
P = 0.012 R² = 0.077
Width (mm)
P = 0.13 R² = 0.029
Depth (mm)
P = 0.849 R² = 0.00046
Depth_80 (mm)
P = 0.482 R² = 0.0063
CGY (mm)
P = 0.837 R² = 0.00054
Convex_Hull (mm²)
P = 0.108 R² = 0.032
Trait's value
Date of release
